# Dual Semiconductor‐Photoredox/Halogen‐Bonding Organocatalyzed Cascade Sulfonylation/Cyclization of Alkynes and Alkenes with RSO_2_Cl

**DOI:** 10.1002/advs.202515993

**Published:** 2025-11-25

**Authors:** Yao‐Hui Wang, Lin‐Heng He, Yu‐Yu Tan, Zi‐Tong Zhang, Yao‐Dan Xu, Rou Ding, Jun Jiang, Wei‐Min He

**Affiliations:** ^1^ School of Chemistry and Chemical Engineering University of South China Hengyang 421001 China

**Keywords:** dual catalysischloride radical, halogen bond, photoredox catalysis, semiconductor, sulfonylation

## Abstract

The dual semiconductor‐photoredox/halogen‐bonding organocatalyzed cascade sulfonylation/cyclization of (hetero)arene‐tethered alkynes and alkenes with RSO_2_Cl is first developed. This method proceeds efficiently under mild reaction conditions, delivering a variety of sulfonylated fused‐(hetero)arenes with good functional group compatibility. Mechanistic investigations reveals that XantPhos interacts with RSO_2_Cl to form a halogen‐bonding complex, which facilitates S─Cl bond cleavage under blue light irradiation. Specifically, the Sr_3_N_2_ semiconductor catalyst promotes heterolytic S─Cl bond cleavage through a SET process, generating a sulfonyl radical and chloride ion. In contrast, the sulfonylated fused‐(hetero)arene facilitates homolytic S─Cl bond cleavage via an EnT pathway, proudcing a sulfonyl radical.

## Graphical abstract

The first dual semiconductor‐photoredox/halogen‐bonding catalyzed cascade sulfonylation/cyclization of arene‐tethered alkynes/alkenes with RSO_2_Cl is reported. Mechanistic studies show that the system facilitates S─Cl bond cleavage via two distinct pathways: the semiconductor enables heterolytic cleavage via SET, while the sulfonylated arene promotes homolytic cleavage through EnT.

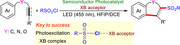

## 1 Introduction

**Scheme 1:**
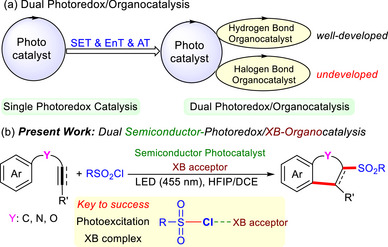
Dual Photoredox/Organocatalysis.

Developing catalytic systems for the efficient transformation of simple raw materials into value‐added chemicals constitutes a core objective in chemistry. Visible‐light photocatalysis has emerged as a powerful and versatile methodology, harnessing sustainable light energy to drive radical transformations under mild conditions.^[^ ^1^, ^2^, ^3^ ^]^ In traditional single photocatalyst systems, the excited photocatalyst activates one substrate via single electron transfer (SET)^[^ ^4^ ^]^ or energy transfer (EnT)^[^ ^5^ ^]^ processes to produce a radical or radical ion species, which can then form a bond with another unactivated substrate. However, this approach proves inadequate for challenging transformations involving low‐reactivity substrates. Recently, dual photoredox/organocatalysis,^[^ ^6^, ^7^, ^8^, ^9^ ^]^ featuring the concerted operation of distinct catalytic cycles involving both photoredox catalysts and organocatalysts, has emerged as a powerful strategy for forming new bonds with un‐activated substrates.^[^ ^10^, ^11^, ^12^, ^13^, ^14^, ^15^ ^]^ Halogen bonds (XB), defined as noncovalent interactions between the electrophilic region of a polarized halogen atom and a Lewis base, have found broad applications across inorganic, organic, and biological chemistry.^[^ ^16^ ^]^ Recently, XB have been extensively leveraged to activate carbon‐halogen bonds in organic halides, enabling their transformation involvement in organocatalysis.^[^ ^17^, ^18^, ^19^, ^20^, ^21^, ^22^, ^23^, ^24^ ^]^ In addition, XB complexes harvest photons with specific energy gaps through charge‐transfer absorption bands, triggering carbon‐halogen bond homolysis via SET to initiate radical reactions.^[^ ^25^, ^26^, ^27^, ^28^, ^29^ ^]^ However, compared to well‐established photoredox/hydrogen‐bonding (HB) organocatalytic systems,^[^ ^30^, ^31^, ^32^ ^]^ synergistic photoredox/XB organiccatalytic platforms remain significantly underexplored (**Scheme** **1**a).

Intrinsic semiconductor photocatalysts exhibit inherent benefits such as cost‐effectiveness and facile reusability. Nevertheless, limitations such as the low efficiency of photogenerated carrier separation and migration, and inefficient interfacial mass transfer generally result in the low photocatalytic performance of intrinsic semiconductor photocatalysts.^[^ ^33^, ^34^ ^]^ As a consequence, single‐component intrinsic semiconductor photocatalysts remain primarily limited to mediating radical reactions of highly reactive substrates due to their inherently low catalytic efficiency.^[^ ^35^, ^36^ ^]^

Sulfonyl chlorides (RSO_2_Cl) are widely employed for constructing various value‐added sulfone‐containing molecules due to their low cost and commercial availability. In recent years, homogeneous photocatalytic cascade sulfonylation/cyclization of (hetero)arene‐tethered alkynes and alkenes with RSO_2_Cl has emerged as an efficient and step‐economical strategy for synthesizing structurally diverse sulfonylated fused‐(hetero)arenes.^[^ ^37^, ^38^ ^]^ However, to the best of our knowledge, the semiconductor‐photocatalyzed cascade reactions with RSO_2_Cl remain unexplored. To address these challenges, we propose a synergistic dual catalytic system integrating semiconductor‐photoredox catalysis with XB organocatalysis, leveraging XB interactions to pre‐activate RSO_2_Cl and thereby facilitating semiconductor‐photocatalzyed transformation of RSO_2_Cl into valuable sulfone compounds. As part of our ongoing photocatalysis research,^[^ ^39^, ^40^, ^41^, ^42^ ^]^ we report a dual semiconductor‐photoredox/XB organic catalytic system for the cascade sulfonylation and cyclization of (hetero)arene‐tethered alkynes and alkenes of RSO_2_Cl. Using Sr_3_N_2_ as the semiconductor photocatalyst and a phosphine‐based XB acceptor, this cascade reaction proceeds smoothly under mild conditions with blue‐light irradiation, efficiently producing structurally diverse sulfonylated fused‐(hetero)arenes (Scheme 1b). In this process, the phosphine facilitates S─Cl bond cleavage in RSO_2_Cl via XB activation.

## 2 Results and Discussion

To test our hypothesis, we selected the cascade reaction of 2‐(phenylethynyl)‐1,1‐biphenyl (**1a**) and 4‐toluenesulfonyl chloride (TsCl, **2a**) as the model reaction for condition optimization (**Table** **1**). Treatment of a mixture of **1a** and **2a** (2 equiv.) with g‐C_3_N_4_ (4 mg) as the semiconductor photocatalyst in HFIP/DCE at room temperature under nitrogen atmosphere with the irradiation of LED (455 nm, 10 W) for 24 h led to the formation of the desired product **3aa** in 19% GC yield (Table 1, entry 1). Screening of various semiconductor photocatalysts, such as CdS, SrO, Sr_3_N_2,_ and CaTiO_3_, revealed that Sr_3_N_2_ provides the best result for this transformation (entries 2–5). In all investigated semiconductor photocatalytic systems, employing XantPhos as an XB acceptor significantly enhanced cascade reaction efficiency (entries 6 – 10), with the combination of Sr_3_N_2_ and XantPhos delivering the optimal performance (entry 9). When other phosphorus (III) compounds were used as XB acceptors, the yield of **3aa** also substantially increased relative to systems without XB acceptors (entries 11–15). These results demonstrate that using phosphorus (III) compounds as XB acceptors can effectively improve the reaction efficiency. Substituting the LED light source with LEDs of different wavelengths (365, 390, and 420 nm) also reduced the yield of **3aa** (entries 16–18). In the absence of any semiconductor photocatalyst but with XantPhos as a XB acceptor, **3aa** was obtained in 20% GC yield (entry 19). No reaction took place without both the semiconductor catalyst and phosphorus (III) compound (entry 20). In addition, the cascade reaction proved inactive under either an air atmosphere or dark conditions (entries 21 and 22).

**Table 1:** Optimization of Conditions.^a)^

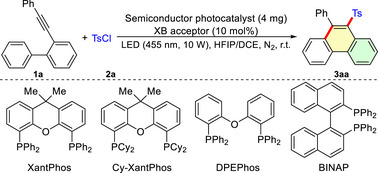				

Entry	Photocatalyst	XB acceptor	LED	Yield^b^ ^)^
1	g‐C_3_N_4_	w/o	455 nm	19%
2	CdS	w/o	455 nm	33%
3	SrO	w/o	455 nm	28%
4	Sr_3_N_2_	w/o	455 nm	35%
5	CaTiO_3_	w/o	455 nm	24%
6	g‐C_3_N_4_	XantPhos	455 nm	49%
7	CdS	XantPhos	455 nm	84%
8	SrO	XantPhos	455 nm	62%
**9**	**Sr_3_N_2_**	**XantPhos**	**455 nm**	**91%**
10	CaTiO_3_	XantPhos	455 nm	59%
11	Sr_3_N_2_	Cy‐XantPhos	455 nm	87%
12	Sr_3_N_2_	DPEPhos	455 nm	57%
13	Sr_3_N_2_	BINAP	455 nm	63%
14	Sr_3_N_2_	Ph_3_P	455 nm	31%
15	Sr_3_N_2_	Cy_3_P	455 nm	42%
16	Sr_3_N_2_	XantPhos	360 nm	31%
17	Sr_3_N_2_	XantPhos	380 nm	37%
18	Sr_3_N_2_	XantPhos	420 nm	51%
19	w/o	XantPhos	455 nm	20%
20	w/o	w/o	455 nm	N.R.
21^c^ ^)^	Sr_3_N_2_	XantPhos	455 nm	N.R.
22	Sr_3_N_2_	XantPhos	w/o	N.R.

^a)^ Conditions: **1a** (0.1 mmol), **2a** (0.2 mmol), semiconductor photocatalyst (4 mg), XB acceptor (0.01 mmol), HFIP (2 mL), DCE (1 mL), LED (10 W), N_2_, room temperature, 24 h;

^b)^ GC yields using dodecane as an internal reference. Conver.: conversion rate; w/o: without; N.R.: no reaction;

^c)^ Under air.

With the optimized reaction conditions established (Table 1, entry 9), the reaction scope was investigated for both RSO_2_Cl and unsaturated carbon‐carbon (**Table** **2**). Pleasingly, this photocatalytic system exhibited broad applicability across diverse RSO_2_Cl. Aryl sulfonyl chlorides bearing sterically hindered (**3ab** and **3ac**), electron‐neutral (**3ad** and **3ae**), electron‐donating (**3aa**, **3af** and **3ag**), or electron‐withdrawing substituents (**3ah** – **3al**) all furnished the desired products in good to excellent yields. The protocol tolerated a series of valuable functional groups on the phenyl ring including methoxyl, fluoro, chloro, bromo, cyano, and ester, offering potential for further late‐stage functionalization.3‐Thienylsulfonyl chloride provided the sulfonylated product **3am** in 71% yield. Furthermore, alkyl sulfonyl chlorides, including cyclopropanesulfonyl chloride cyclohexanesulfonyl chloride and 1‐butanesulfonyl chloride, were efficiently converted into the corresponding products **3an** – **3ap** in good yields. 2‐Alkynyl biaryl **1** featuring phenyl ring **I** substituted with sterically hindered (**3ba** and **3ca**), electron‐neutral (**3** **da**), electron‐donating (**3ea** and **3fa**), or electron‐withdrawing groups (**3ga** – **3ia**) reacted efficiently with **2a**, delivering the desired products in 82%‐93% yields. Thienyl‐substituted ethynyl biphenyl successfully participated in the cascade reaction to afford the corresponding product **3ja** in 69% yield. Similarly, substitutions on phenyl ring **II** of substrate 1, whether sterically hindered (**3ka** and **3la**), electron‐donating (**3ma** and **3na**), or electron‐withdrawing (**3oa** – **3qa**) had minimal impact on this transformation, delivering the target sulfonylated phenanthrenes in good to excellent yields. To further explore the potential of the dual catalytic strategy, this reaction was extended to other unsaturated C─C bonds. For instance, alkynyl compounds such as *N*‐phenylpropiolamide and phenyl 3‐phenylpropiolate proved effective, affording the desired sulfonylated quinolinone **3ra** and sulfonylated chromenone **3sq** in 68% and 64% yield, respectively. Notably, the reaction scope also extends to functionalized alkenes, such as *N*‐allyl benzamide and acryloylated 2‐phenyl benzoimidazoles, which furnish the corresponding heterocycles in moderate yields.

**Table 2:** Substrate Scope.^a)^

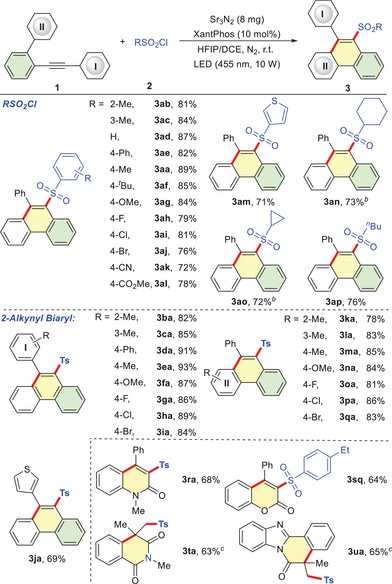

^a)^ **1** (0.2 mmol), **2** (0.4 mmol), Sr_3_N_2_ (8 mg), XantPhos (0.02 mml), N_2_, HFIP (2 mL), DCE (1 mL), LED (455 nm, 10 W), r.t;

^b)^ Cy_3_P was used.

^c)^ DABCO (0.4 mmol) was added.

**Scheme 2:**
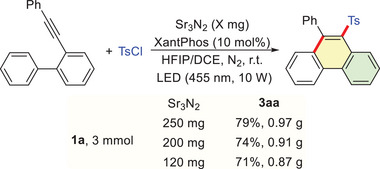
Gram‐scale Synthesis of **3aa**.

**Figure 1:**
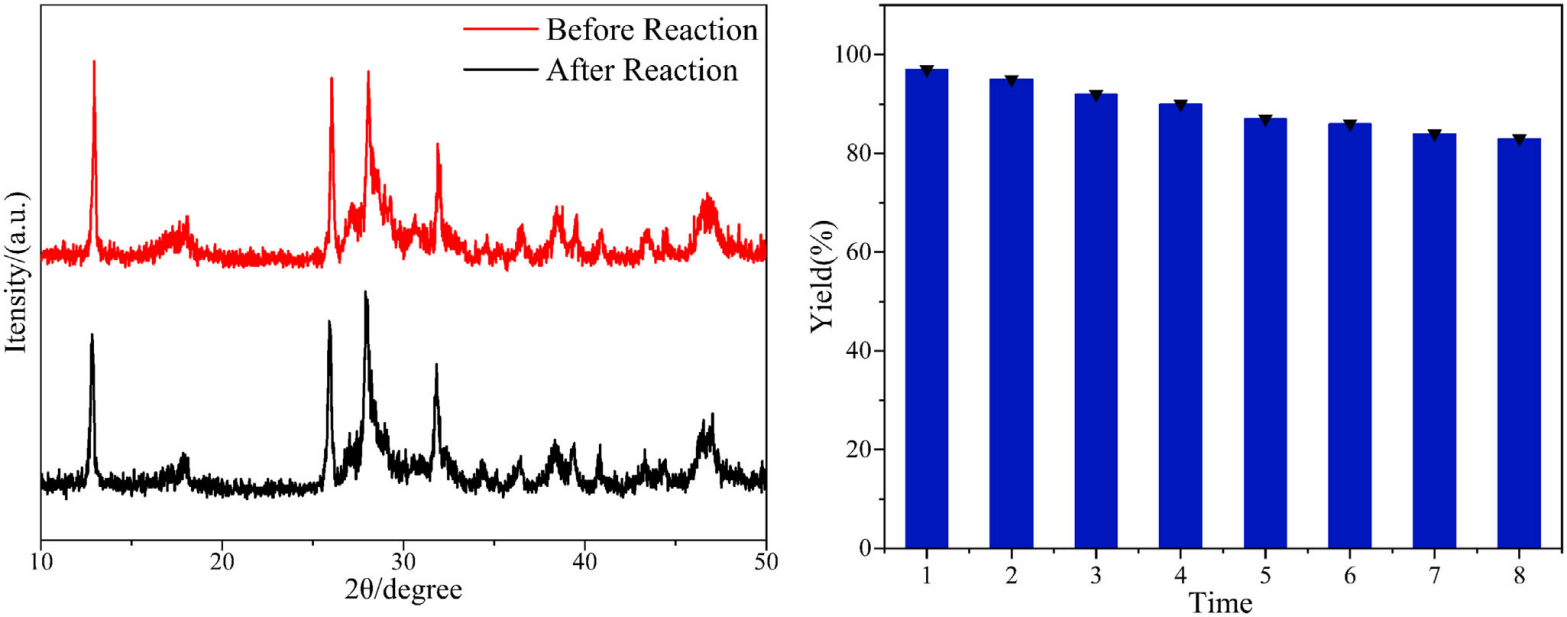
a) The Reusability of Sr_3_N_2_; b) The XRD of Sr_3_N_2_.

From a practical perspective, scalability and photocatalyst reusability are key factors for photocatalytic systems. The scalability of this protocol was demonstrated through a gram‐scale synthesis at 3 mmol, which provided product **3aa** in 79% yield with 250 mg of Sr_3_N_2_. Moreover, the reaction maintained a good yield of **3aa** even with a reduced loading of Sr_3_N_2_ (**Scheme** **2**). Furthermore, the reusability and stability of the semiconductor photocatalyst were assessed over eight consecutive recycling experiments. The Sr_3_N_2_ was efficiently recovered by centrifugation and reused without significant loss of activity (**Figure** **1**a). The X‐ray diffraction (XRD) analysis corroborated this stability, showing essentially unchanged patterns before and after reaction cycles (Figure 1b).

**Scheme 3:**
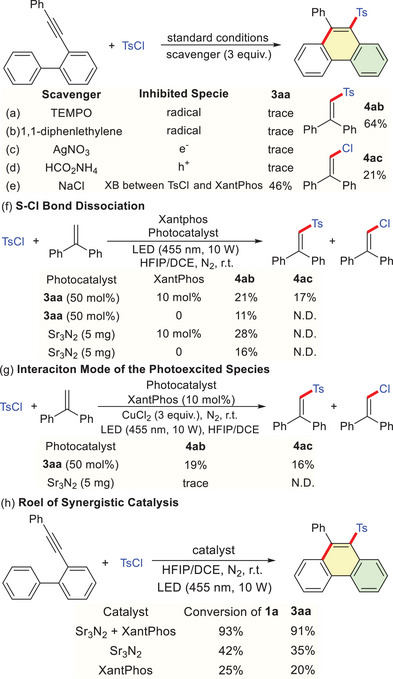
Control Experiments.

To gain deeper insight into the mechanism of this dual catalytic reaction, a set of mechanistic studies was conducted (**Scheme** **3**). The addition of radical scavengers (TEMPO or 1,1‐diphenylethene) under standard conditions completely inhibited this transformation (Scheme 3a,b). GC‐MS detection of Ts‐diphenylethene adduct (**4ab**, 64%) and Cl‐diphenylethene adduct (**4ac**, 21%) suggested the formation of Ts radical (Ts˙) and Cl radical (Cl˙) in this process. Using either the photo‐generated electron (e^−^) scavenger AgNO_3_ or the photo‐generated hole (h⁺) scavenger HCO_2_NH_4_ afforded only trace amounts of **3aa** (Scheme 3c,d), indicating that both e^−^ and h⁺ are essential for the reaction. Furthermore, the addition of chloride anion (Cl^−^) as an XB acceptor significantly suppressed this cascade reaction (Scheme 3e), indicating that XB between **2a** and XantPhos is critical for reactivity.^[^ ^43^ ^].^

When exclusively employing **3aa** as the photosensitizer (without Sr_3_N_2_) in the presence of a SET scavenger CuCl_2_,^[^ ^44^ ^]^ homolysis of XantPhos‐activated **2a** was driven via an EnT pathway under blue light irradiation, affording Ts˙ and Cl˙. In contrast, when Sr_3_N_2_ was used, it induces heterolysis of XantPhos‐activated **2a** through a SET pathway, selectively generating Ts˙ and Cl^−^ (Scheme 3f,g).

GC‐MS analysis of the reaction revealed that synergistic catalysis between Sr_3_N_2_ and XantPhos resulted in a significantly higher yield of **3aa** and the concurrent suppression of side reactions, compared to using either component alone (Scheme 3h).

**Scheme 4:**
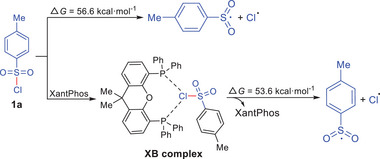
Computational Study of the Free Energy for Sulfonyl Radical Formation.

As shown in **Scheme** **4**, the direct homolytic cleavage of the S─Cl bond in 1a to produce a Ts˙ and a Cl˙ is endergonic by 56.6 kcal mol^−1^. However, this homolysis process can be facilitated by the formation of a XB complex. Upon formation of the XB complex, the energy required for homolytic cleavage is significantly reduced (ΔG = 53.6 kcal mol^−1^) (See Supporting Information).

**Figure 2:**
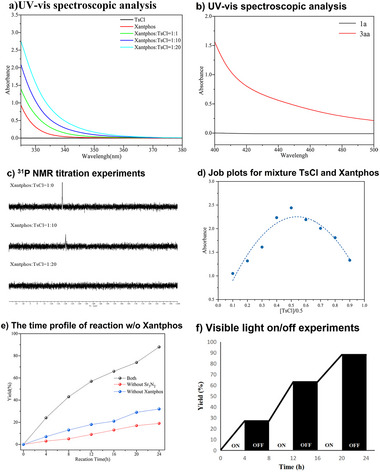
Mechanistic Studies.

The UV–vis spectroscopic analysis revealed a bathochromic shift in the absorption of the mixtures of **2a** and XantPhos, indicating an interaction between **2a** and XantPhos (**Figure** **2**a). Notably, among all tested species, including **1a**, **2a**, **3aa**, XantPhos, and the mixtures of **2a** with XantPhos, only **3aa** exhibited detectable absorption within 400–500 nm. This unique photophysical property enables its direct photoexcitation under blue LED irradiation (416–517 nm, see Supporting Information) to produce excited‐state species (Figure 2a,b), thereby designating **3aa** as the photosensitizer responsible for initiating photocatalytic EnT processes.^[^ ^45^, ^46^ ^] 31^P NMR titration experiments suggested that the shifts of the phosphorus signals of XantPhos moved toward the low field when the ratio of TsCl to XantPhos, which also strongly supported the existence of noncovalent interactions between TsCl to XantPhos (Figure 2c). The Job plot analysis confirmed a 1:1 complex formation between XantPhos and TsCl (Figure 2d). The time profile of template reaction confirmed that the simultaneous addition of Sr_3_N_2_ and XantPhos significantly enhances the efficiency of the cascade reaction (Figure 2e).

Light‐on/off experiments confirmed that continuous irradiation is essential for this reaction to proceed (Figure 2f). Furthermore, the apparent quantum efficiency (*Φ*) for the model reaction at 455 nm was calculated as 0.3%, suggesting that a radical‐chain process is unlikely involved in the photocatalytic process (See Supporting Information).

**Scheme 5:**
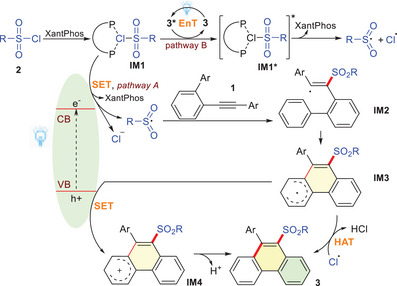
Possible Reaction Mechanism.

Based on the preliminary mechanistic studies and relevant literature,^[^ ^46^, ^47^, ^48^, ^49^ ^]^ the mechanism for the overall transformation can be proposed (**Scheme** **5**), with a key question concerning the generation of the Cl^−^ and Cl˙. Two distinct pathways are conceivable. Pathway A: Initially, the XB complex **IM1** formed in situ via noncovalent interaction between RSO_2_Cl **2** and XantPhos. Under blue light irradiation, the semiconductor Sr_3_N_2_ absorbed photons, generating an electron in the conduction band (CB) and a hole in the valence band (VB). Subsequently, **IM1** received an electron from the VB, leading to the formation of the sulfonyl radical (RSO_2_˙), Cl^−^, and the release of XantPhos. The RSO_2_˙ then attacked the alkynyl moiety of substrate **1a** to form a radical intermediate **IM2**, which underwent the intramolecular cyclization to produce a radical **IM3**. Finally, the hole oxidized the **IM3** via a SET process into the cationic intermediate **IM4**, which underwent a dehydrogenation process to afford the target product **3**. Pathway B: The ground‐state product **3** was excited by blue light irradiation to form its excited‐state species 3^*^, which then underwent an EnT process with the XB complex **IM1** to produce the excited‐state IM1^*^ and re‐generate ground‐state product **3**. Subsequently, homolytic cleavage of the S─Cl bond in IM1^*^ occurred, yielding an RSO_2_˙ radical, a Cl**˙**, and releasing a XantPhos. RSO_2_˙ then initiated a cascade sulfonylation/cyclization reaction to afford radical intermediate **IM3**. Finally, hydrogen atom transfer (HAT) from **IM3** to the chloride radical yielded the product **3**. The optimization of reaction conditions indicated that pathway A as the dominant pathway, with pathways B operating as minor pathways.

## 3 Conclusion

To summarize, we have first presented the dual semiconductor‐photoredox/XB organocatalysis for the cascade sulfonylation/cyclization of (hetero)arene‐tethered alkynes and alkenes with RSO_2_Cl. This strategy exhibits broad substrate compatibility, accommodating various functionalized alkynes, alkenes, and RSO_2_Cl to afford a variety of sulfonylated fused‐(hetero)arenes (36 examples) in good to excellent yields. This process is easily scalable to gram quantities and enables efficient recycling of the semiconductor photocatalyst. Mechanistic studies revealed that the formation of a XB complex between RSO_2_Cl and XantPhos is crucial for activating RSO_2_Cl. Notably, Sr_2_N_2_ promotes heterolytic S─Cl bond cleavage in this activated XB complex via a SET process, generating RSO_2_˙ and Cl^−^. Conversely, the sulfonylated fused‐(hetero)arene facilitates homolytic S─Cl bond cleavage through an EnT pathway, yielding RSO_2_˙ and Cl˙. The low cost and commercial availability of Sr_2_N_2_ and XantPhos, combined with mild conditions, gram‐scale scalability, and robust recyclability of the semiconductor photocatalyst, render this dual catalytic system highly competitive. We anticipate that this strategy not only provides an eco‐friendly synthetic route to sulfonylated fused‐(hetero)arenes but also advances XB organocatalysis and intrinsic semiconductor photocatalysis.

## Conflict of Interest

The authors declare no conflict of interest.

## Supporting information



Supporting Information

## Data Availability

The data that support the findings of this study are available in the supplementary material of this article.
