# Supplementary material for: Dual Semiconductor‐Photoredox/Halogen‐Bonding Organocatalyzed Cascade Sulfonylation/Cyclization of Alkynes and Alkenes with RSO2Cl
Source: Adv Sci (Weinh). 2025 Nov 25;13(4):e15993. doi: 10.1002/advs.202515993 (PMC12822463; doi:10.1002/advs.202515993)

Supporting Information

Dual Semiconductor-Photoredox/Halogen-Bonding Organocatalyzed Cascade Sulfonylation/Cyclization of Alkynes and Alkenes with RSO_2Cl

Yao-Hui Wang, Lin-Heng He, Yu-Yu Tan, Zi-Tong Zhang, Yao-Dan Xu, Rou Ding, Jun Jiang, Wei-Min He*

School of Chemistry and Chemical Engineering, University of South China, Hengyang 421001, China

Email: weiminhe@usc.edu.cn

Table of contents

1. General Considerations S2
2. General Procedure for the Synthesis of Starting Materials S4
3. Optimization of Reaction Conditions S6
   3.1 Procedure for the Synthesis of **3aa** : S6
   3.2 Evaluation of XB Acceptor: S6
   3.3 Evaluation of Semiconductor Photocatalyst and XB Acceptor: S7
   3.4 Evaluation of Solvent: S7
   3.5 Evaluation of Light Source and Atmosphere: S8
   3.6 Large-Scale Synthesis of **3aa**: S8
   3.7 Mechanism Exploration Experiments: S9
   3.8 UV-vis Absorption Spectra: S12
   3.9 NMR Titration Experiments: S13
   3.10 Visible ON-OFF Experiments: S14
   3.11 Reusability and Stability of Sr_3N_2 Experiments: S14
   3.12 The Complete Reaction Profile of Model Reaction with/without Sr_3N_2 and XantPhos S15
   3.13 XRD Analysis Experiments: S15
   3.14 The Job's Plot Analysis Experiments: S16
   3.15 Determination of Quantum Yield: S16
   3.16. Computational Details： S17
4. Characterization Data of Products S24
5. Reference S36
6. ^{1}H NMR, ^{13}C NMR and ^{19}F NMR Spectra of Products S37

1. General Considerations

All manipulations were conducted with a standard Schlenk tube under a nitrogen atmosphere. Unless otherwise noted, all reagents and solvents were obtained from commercial suppliers and used without further purification. Sr_3N_2 (product ID: 013534840, particle diameter: 5-10μm) was purchased from Shanghai Titan Scientific Co., ltd. Thin layer chromatography (TLC) employed glass 0.25 mm silica gel plates. Column chromatography was carried out on silica gel (200-300 mesh). ^{1}H NMR spectra were recorded at 400 MHz or 500 MHz, ^{13}C NMR spectra were recorded at 101 MHz or 126 MHz and ^{19}F NMR spectra were recorded at 471 MHz or 376 MHz by using a Quantum-Iplus 400 MHz or Bruker Avance 500 MHz. Chemical shifts were calibrated using residual undeuterated solvent as an internal reference (^{1}H NMR: $CDCl_3$-d 7.26 ppm, ^{13}C NMR: $CDCl_3$-d 77.0 ppm), the chemical shifts (δ) were expressed in ppm and J values were given in Hz. The following abbreviations were used to describe peak splitting patterns when appropriate: s = singlet, d = doublet, t = triplet, q = quartet, m = multiplet, dd = doublet of doublets, br = broad.

The Light Source and the Material of the Irradiation Vessel

Manufacturer: Beijing Roger-tech Ltd.

Model: OHSP-350UV

Wave: 455.0nm Value: 8326.436uW/cm²/nm

Energy peak wavelength: 453.0nm

Peak width at half height: 22.1nm

Material of the irradiation vessel: Schlenk flask.

Not use any filters.

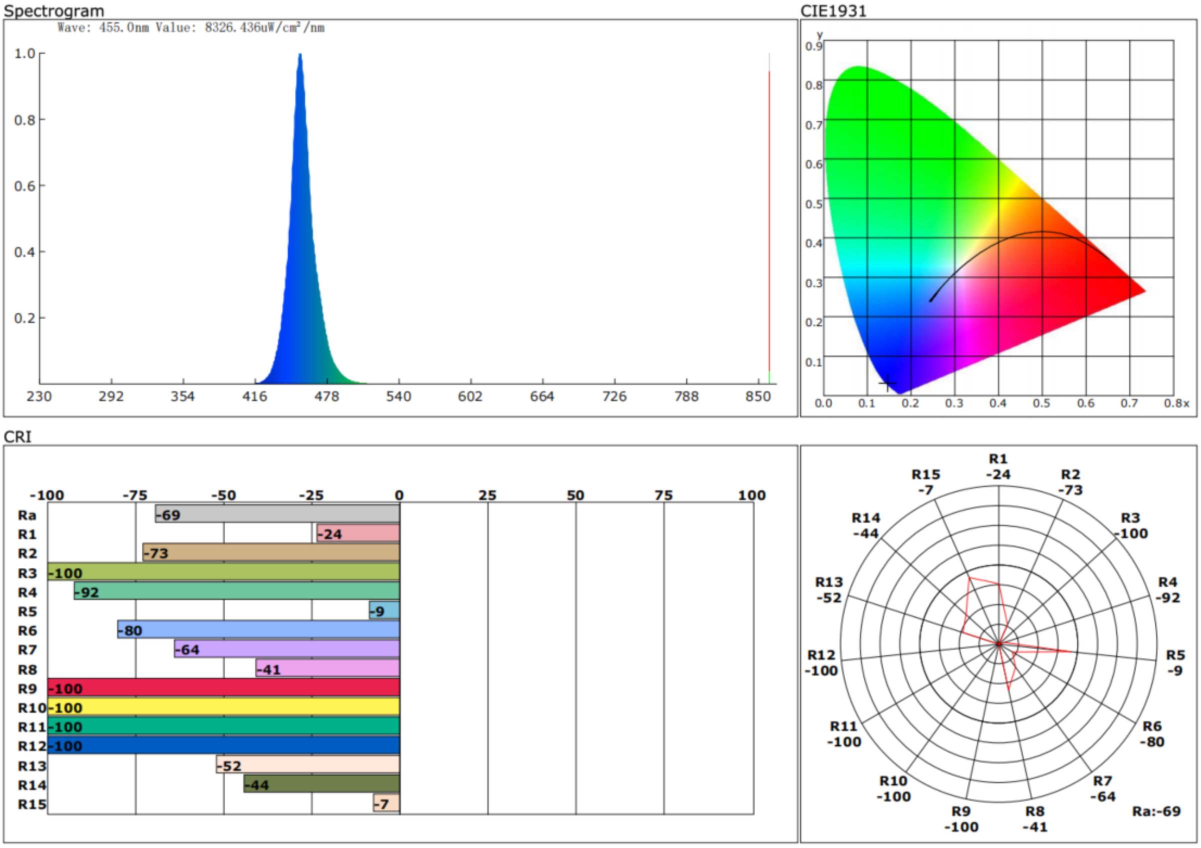

Figure S1. LED spectrum test report.

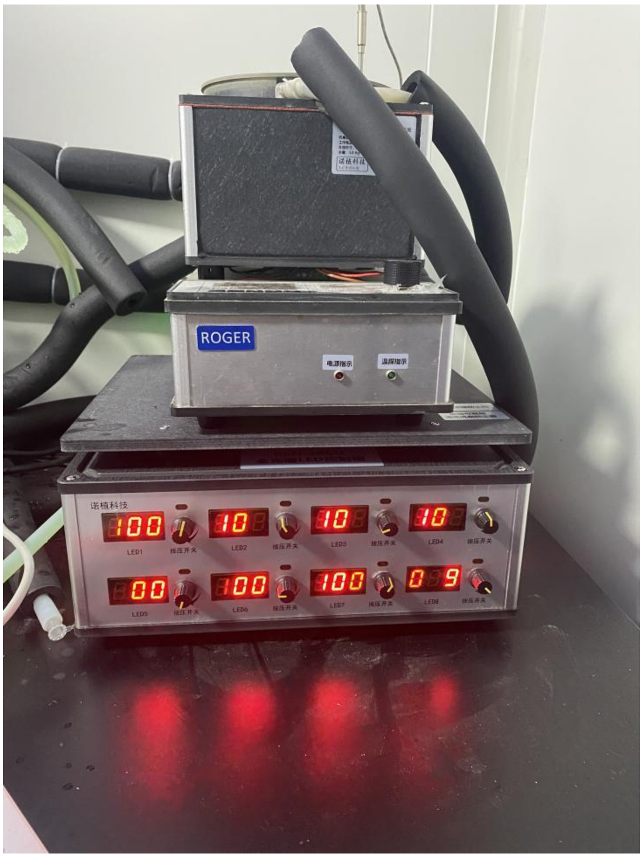

Figure S2. Photograph of Photoreaction Instrument

2. General Procedure for the Synthesis of Starting Materials[1]

2.1 Preparation of Ar_1 Substituted Substrates

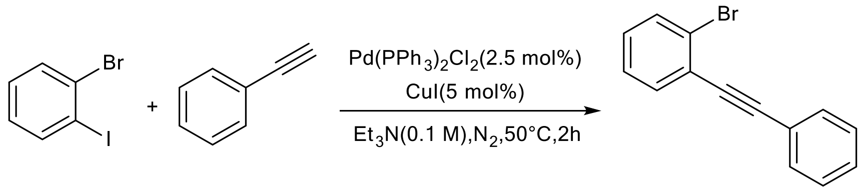

Representative Procedure for Sonogashira Cross-coupling Reaction: To a 250 mL round-bottomed flask with a stir bar was added 1-Bromo-2-iodobenzene (2829.1 mg, 10 mmol, 1.0 equiv.), $Pd(PPh_3)_2Cl_2$ (175.48 mg, 2.5 mol%), CuI (95.23 mg, 5 mol%) in 30 mL triethylamine was degassed with nitrogen for 5 minutes. Then, a solution of phenyl acetylene (1225.56 mg, 1.2 equiv.) in triethylamine (5 mL) was added drop-wise over 5 minutes via syringe and the reaction mixture was reacted at 50 ℃ for 2 h. After completing consumption of the 2-bromoiodobenzene, as monitored by TLC. The reaction mixture was filtered through pad of celite and extracted with ethyl acetate (3 × 15 mL). The organic layer was washed with a saturated solution of brine (3 × 15 mL), water (3 × 15 mL), dried over Na_2SO_4 and the organic solvent was removed under vacuo. The reaction mixture was purified by flash chromatography on silica gel by petroleum ether to give the yellow oil named 1- bromo-2-(phenylethynyl) benzene in 97% yield.

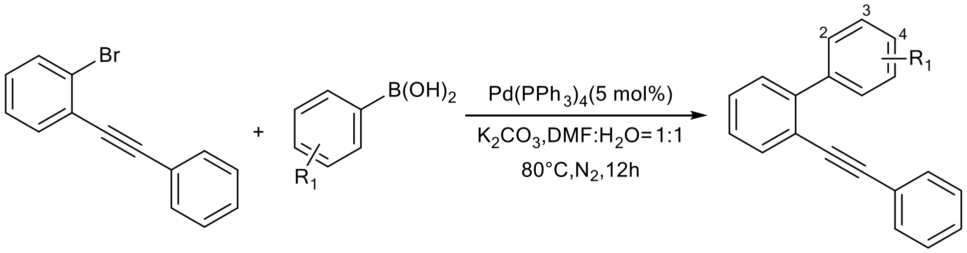

Representative Procedure for Suzuki Cross-coupling Reaction: To a 100 mL round-bottomed flask with a stir bar was added 1-bromo-2-phenylethynyl-benzene (1285.65 mg, 5 mmol, 1.0 equiv.) and phenylboronic acid (731.58 mg, 6 mmol, 1.2 equiv.) in DMF/H_2O=1:1 (20 mL) was degassed with nitrogen for 5 minutes. Then, K_2CO_3 (2073.08 mg, 3.0 equiv.) and $Pd(PPh_3)_4$ (288.89 mg, 5 mol%) were added under nitrogen atmosphere. The reaction mixture was stirred at 80 ℃ for 6h. After completing consumption of starting material, as monitored by TLC, the resulting mixture was allowed to bring to room temperature. The reaction mixture was diluted with $NaHCO_3$ (*aq*) (10 mL) and then the product was extracted with ethyl acetate (3×15 mL). The combined organic layer was dried over Na_2SO_4 and the organic solvent was removed under vacuo.

The crude product was purified on a silica gel column using petroleum ether/ethyl acetate as eluent to afford the colorless oil named 2-(phenylethynyl)-1, 1-biphenyl in 82% yield.

2.2 Preparation of Ar_2 substituted substrates

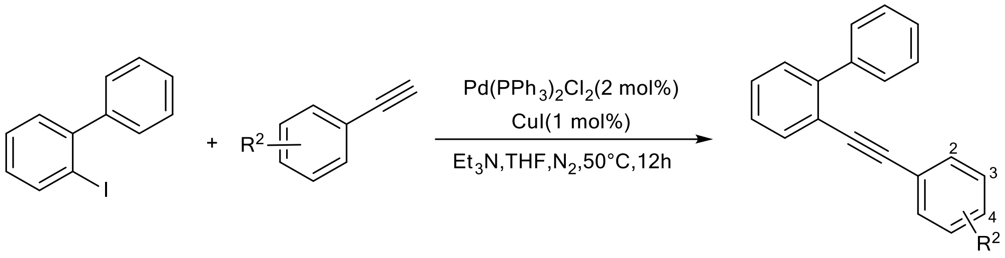

Representative Procedure for Sonogashira Cross-coupling Reaction: Add a whisk to a 250 mL three-neck flask, add 2-iodobiphenyl (5602 mg, 20 mmol, 1.0 equiv.), dissolved in 10 mL triethylamine and 30 mL tetrahydrofuran. Weigh the $Pd(PPh_3)_2Cl_2$ (281 mg, 0.4 mmol, 0.02 equiv.) and CuI (38.1 mg, 0.2 mmol, 0.01 equiv.) as catalysts was introduced into the reaction solution. Subsequently, in the nitrogen atmosphere, one-hour reaction at 50 ℃.Then, draw the phenylacetylene (2451.12 mg, 24 mmol, 1.2 equiv.) with a syringe, slowly injected into the reaction solution (At this point, the reaction solution was gradually changed from yellow to dark brown). Later, the reaction continued in the nitrogen atmosphere for 12 h. The reaction solution color changes from black to brown-yellow. After the reaction, TLC (petroleum ether as unfolding agent) was tested, filtered to remove the solid, washed with ethyl acetate (3×20 mL), add saturated saline (3×10 mL), water (3×40 mL), extract the upper organic phase, dried with anhydrous sodium sulfate, filtered, and evaporated, column chromatography to obtain colorless oil product (petroleum ether/ethyl acetate=40:1 as elution agent).

3. Optimization of Reaction Conditions

3.1 Procedure for the Synthesis of **3aa:**

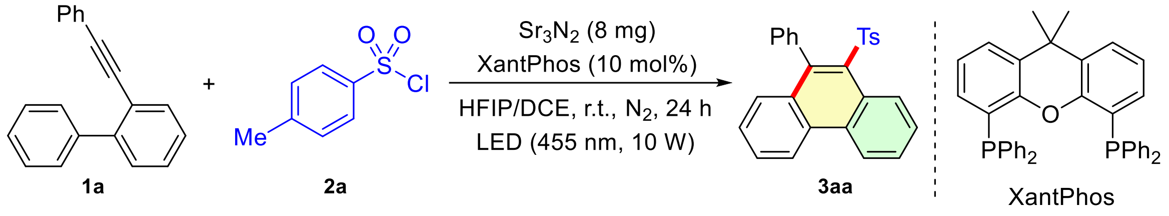

To a 15 mL quartz tube equipped with a magnetic stirring bar were added 2-(phenylethynyl)-1,1-biphenyl (**1a**, 0.2 mmol), TsCl (**2a**, 0.4 mmol), Sr_3N_2 (8 mg), XantPhos (0.02 mmol), HFIP (2 mL) and DCE (1 mL). The reaction mixture was stirred at room temperature under the irradiation of LED (455 nm, 10 W) for 24 h. After the completion of the reaction, the solvent was concentrated under reduced pressure and the insoluble were removed by filtration and the filtrate were purified by flash column to afford product **3aa**.

3.2 Evaluation of XB Acceptor[a,b]:

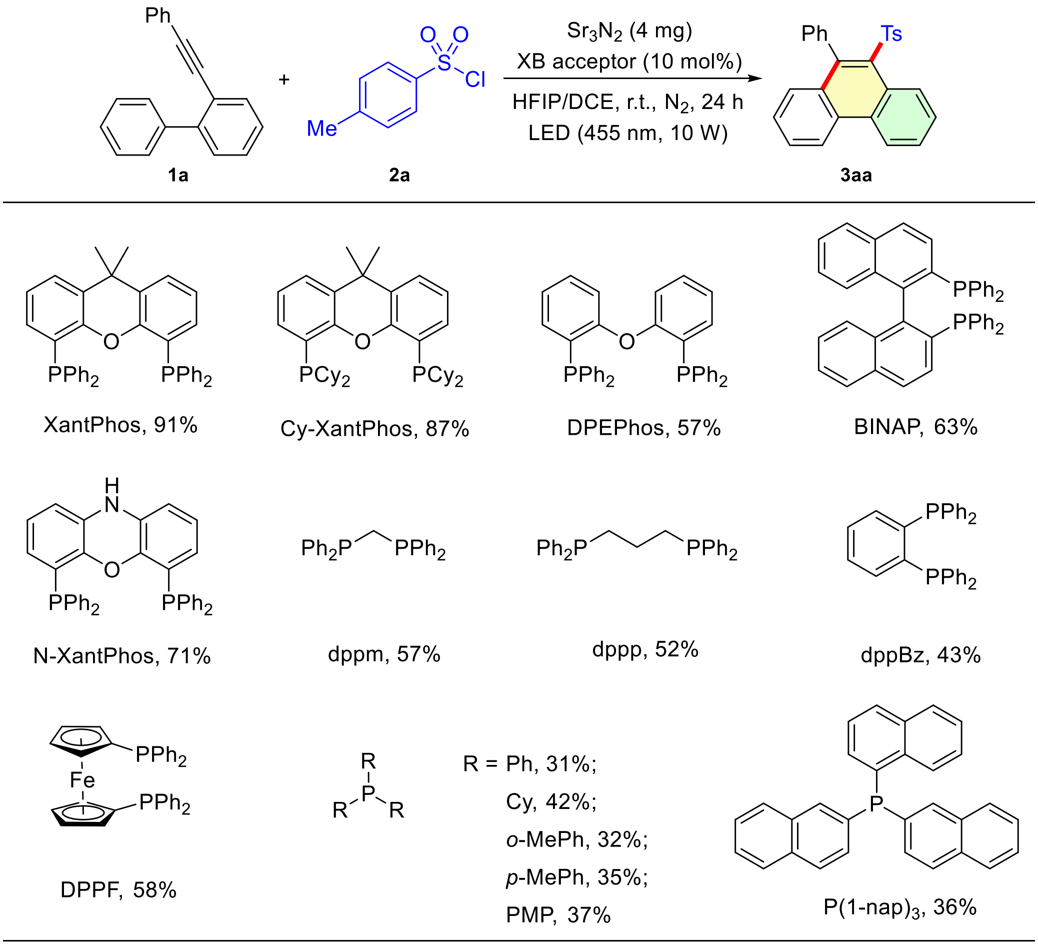

[a] Reaction conditions: **1a** (0.1 mmol), **2a** (0.2 mmol), Sr_3N_2 (4 mg), and XB acceptor (0.01 mmol), HFIP (2 mL) and DCE (1 mL) at room temperature under N_2 with LED (455 nm, 10 W) for 24 h. [b] Estimated by GC-MS with dodecane as the internal reference.

3.3 Evaluation of Semiconductor Photocatalyst and XB Acceptor [a]:

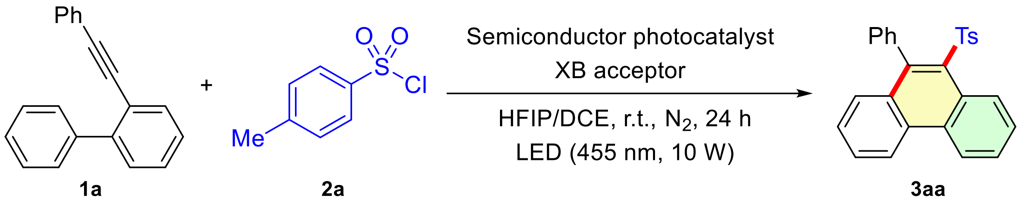

Entry	Semiconductor photocatalyst (mg)	XB acceptor (mmol)	Yield[b] (%)
1	g-C_3N_4, CdS, $CaTiO_3$, CW_2 (4)	w/o	19, 33, 24, 27
2	SrO, $SrBaTiO_4$, $SrWO_4$ (4)	w/o	28, 29, 24
3	Sr_3N_2 (4)	w/o	35
4	g-C_3N_4, CdS, $CaTiO_3$, CW_2 (4)	XantPhos (0.01)	49, 84, 59, 62
5	SrO, $SrBaTiO_4$, $SrWO_4$ (4)	XantPhos (0.01)	62, 67, 53
**6**	**Sr_3N_2 (4)**	**XantPhos** (0.01)	**91**
7	Sr_3N_2 (8)	w/o	34
8	Sr_3N_2 (12)	w/o	31
9	Sr_3N_2 (4)	XantPhos (0.005)	73
10	Sr_3N_2 (4)	XantPhos (0.02)	90
11	w/o	XantPhos (0.02)	20
12	w/o	w/o	N.R.

[a] Reaction conditions:**1a** (0.1 mmol), **2a** (0.2 mmol), semiconductor photocatalyst and XB acceptor, HFIP (2 mL) and DCE (1 mL) at room temperature under N_2 with LED (455 nm, 10 W) for 24 h. [b] Estimated by GC-MS with dodecane as the internal reference.

3.4 Evaluation of Solvent [a]:

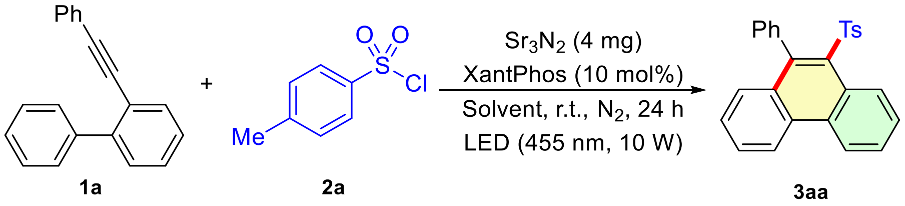

Entry	Solvent (mL)	Yield[b] (%)
1	HFIP, DCE, DCM was used (3)	29, 38, 35
2	HFIP/DCE (1/1) (3)	80
3	HFIP/DCE (1/2) (3)	69

4	HFIP/DCM (1/1) (3)	67
5	HFIP/THF (1/1) (3)	56
**6**	**HFIP/DCE (2/1)** (3)	**91**
7	DMF, DMA, CH_3CN (3)	N.R.

[a] Reaction conditions: **1a** (0.1 mmol), **2a** (0.2 mmol), Sr_3N_2 (4 mg), XantPhos (0.01 mmol), solvent (3 mL) at room temperature under N_2 with LED (455 nm, 10 W) for 24 h. [b] Estimated by GC-MS with dodecane as the internal reference.

3.5 Evaluation of Light Source and Atmosphere[a]:

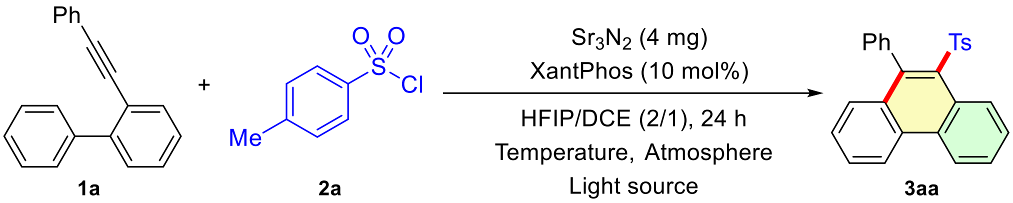

Entry	Light source	Temperature	Atmosphere	Yield[b] (%)
1	LED (455 nm, 3 W)	r.t.	N_2	23
2	LED (455 nm, 6 W)	r.t.	N_2	48
**3**	**LED (455 nm, 10 W)**	**r.t.**	$\mathbf{N_2}$	**91**
4	LED (360 nm, 10 W)	r.t.	N_2	31
5	LED (380 nm, 10 W)	r.t.	N_2	37
6	LED (420 nm, 10 W)	r.t.	N_2	51
7	LED (520 nm, 10 W)	r.t.	N_2	57
8	LED (455 nm, 10 W)	60°C	N_2	12
9	LED (455 nm, 10 W)	r.t.	Air	N.R.
10	LED (455 nm, 10 W)	r.t.	O_2	N.R.
11	w/o	r.t.	N_2	N.R.

[a] Reaction conditions:**1a** (0.1 mmol), **2a** (0.2 mmol), Sr_3N_2 (4 mg) and XantPhos (0.01 mmol), HFIP (2 mL) and DCE (1 mL) for 24 h. [b] Estimated by GC-MS with dodecane as the internal reference.

3.6 Large-Scale Synthesis of **3aa**:

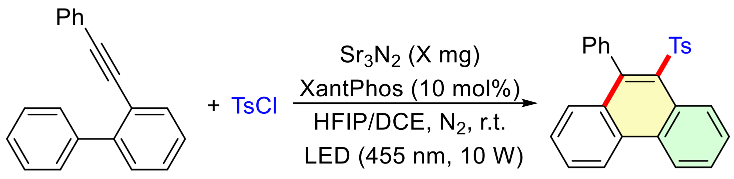

	Sr_3N_2	**3aa**
**1a**, 3 mmol	250 mg	79%, 0.97 g
	200 mg	74%, 0.91 g
	120 mg	71%, 0.87 g

The gram-scale reaction was performed in an over dried 100-mL flask equipped with a stir bar, **1a** (1271.65 mg), **2a** (2.0 equiv. 1906.5 mg), Sr_3N_2 (250 mg), XantPhos (0.3 mmol, 173.59 mg), HFIP (20 mL) and DCE (10 mL) were added. The reaction mixture was stirred under nitrogen atmosphere and irradiated by LED (455 nm, 10 W) at room temperature for about 48 h. The resulting mixture was purified by column chromatography on silica gel (PE/EA = 10:1) to afford **3aa** in 79% (0.97 g). The reaction was performed using 200 mg and 120 mg of Sr_3N_2, which afforded product **3aa** in 74% (0.91 g) and 71% yield (0.87 g), respectively.

3.7 Mechanism Exploration Experiments:

3.7.1 Control Experiments:

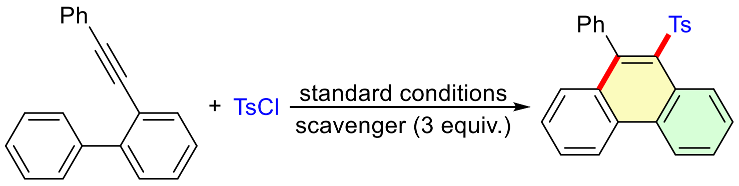

	Scavenger	Inhibited Specie	3aa
(a)	TEMPO	radical	trace
(b)	1,1-diphenlethylene	radical	trace
(c)	$AgNO_3$	e^-	trace
(d)	HCO_2NH_4	h^+	trace
(e)	NaCl	XB (TsCl-XantPhos)	46%

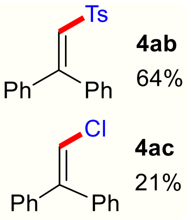

a) To an over-dried 15-mL Schlenk flask equipped with a magnetic stirring bar were added **1a** (0.1 mmol), **2a** (0.2 mmol), Sr_3N_2 (4 mg), XantPhos (0.01 mmol), TEMPO (0.3 mmol), HFIP (2 mL) and DCE (1 mL). The reaction mixture was stirred at room temperature and under nitrogen atmosphere with LED (455 nm, 10 W) irradiation for 24 h. The reaction mixture was analyzed by GC-MS and only a trace amount of **3aa** was detected.

b) To an over-dried 15-mL Schlenk flask equipped with a magnetic stirring bar were added **1a** (0.1 mmol), **2a** (0.2 mmol), Sr_3N_2 (4 mg), XantPhos (0.01 mmol) and 1,1-diphenylethylene (0.3 mmol), HFIP (2

mL) and DCE (1 mL). The reaction mixture was stirred at room temperature and under nitrogen atmosphere with LED (455 nm, 10 W) irradiation for 24 h. The reaction mixture was analyzed by GC, with detection of a trace amount of **3aa**, **4ab** in 64% yield, and **4ac** in 21% yield.

c) To an over-dried 15-mL Schlenk flask equipped with a magnetic stirring bar were added **1a** (0.1 mmol), **2a** (0.2 mmol), Sr_3N_2 (4 mg), XantPhos (0.01 mmol), $AgNO_3$ (0.3 mmol), HFIP (2 mL) and DCE (1 mL). The reaction mixture was stirred at room temperature and under nitrogen atmosphere with LED (455 nm, 10 W) irradiation for 24 h. The reaction mixture was analyzed by GC-MS and only a trace amount of **3aa** was detected.

d) To an over-dried 15-mL Schlenk flask equipped with a magnetic stirring bar were added **1a** (0.1 mmol), **2a** (0.2 mmol), Sr_3N_2 (4 mg), XantPhos (0.01 mmol), HCO_2NH_4 (0.3 mmol), HFIP (2 mL) and DCE (1 mL). The reaction mixture was stirred at room temperature and under nitrogen atmosphere with LED (455 nm, 10 W) irradiation for 24 h. The reaction mixture was analyzed by GC-MS and only a trace amount of **3aa** was detected.

e) To an over-dried 15-mL Schlenk flask equipped with a magnetic stirring bar were added **1a** (0.1 mmol), **2a** (0.2 mmol), Sr_3N_2 (4 mg), XantPhos (0.01 mmol), NaCl (0.3 mmol), HFIP (2 mL) and DCE (1 mL). The reaction mixture was stirred at room temperature and under nitrogen atmosphere with LED (455 nm, 10 W) irradiation for 24 h. The reaction mixture was analyzed by GC-MS and 46% yield of **3aa** was detected.

3.7.2 EnT, SET Process Experiments:

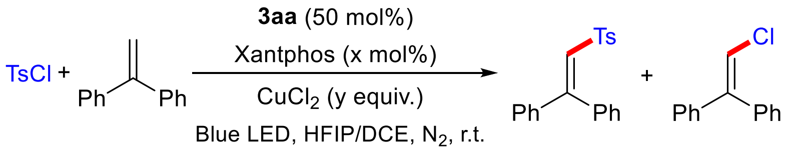

x	y	**4ab**	**4ac**
10	0	21%	17%
0	0	11%	N.D.
10	3	19%	16%

In this experiment, we precisely controlled the amounts of **3aa**, XantPhos, and $CuCl_2$ to investigate the EnT process. When both **3aa** and XantPhos were present, we successfully captured both sulfonyl radicals and chlorine radicals. Notably, sulfonyl and chlorine radicals were still detectable in the sole presence of **3aa** with a decreased yield (as determined by GC). Interestingly, increasing the amount of $CuCl_2$ to 3.0 equiv. did not significantly alter the yields of these radicals. This finding confirms that the coexistence of **3aa** and

XantPhos synergistically catalyzes the reaction, leading to the homolytic cleavage of **2a** exclusively via the EnT process.

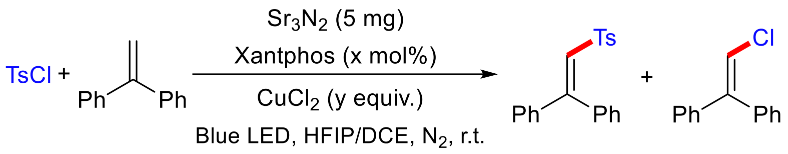

x	y	**4ab**	**4ac**
10	0	28%	N.D.
0	0	16%	N.D.
10	3	trace	N.D.

We further investigated the catalytic mechanism between Sr_3N_2 and XantPhos. In this experiment, we controlled the amounts of XantPhos and $CuCl_2$. When XantPhos was present, 1,1-diphenylethylene exclusively trapped the sulfonyl radical, whereas the yield of **4ab** decreased in the sole presence of strontium nitride. This indicates that XantPhos can activate the S-Cl bond to accelerate its cleavage, generating the sulfonyl radical. Notably, only trace amounts of the sulfonyl radical were detected upon the addition of $CuCl_2$, and no chlorine radical was observed in any of the three experiments. These results suggest that XantPhos accelerates the homolytic cleavage of the S-Cl bond in **2a**, whereas Sr_3N_2 alone catalyzes the heterolytic cleavage of the S-Cl bond, generating the sulfonyl radical and chloride anion via a SET process exclusively.

3.7.3 The Conversion Rates and Product Yields:

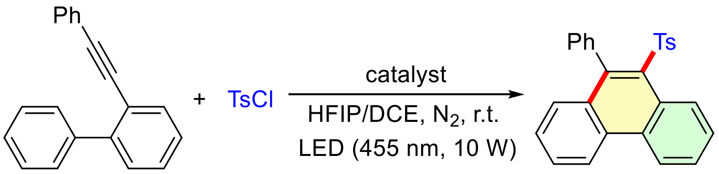

Catalyst	Conversion of **1a**	**3aa**
Sr_3N_2 + XantPhos	93%	91%
Sr_3N_2	42%	35%
XantPhos	25%	20%

1a (0.1 mmol), **2a** (0.2 mmol), Sr_3N_2 (4 mg), XantPhos (0.01 mmol) in HFIP (2 mL), DCE (1 mL) were in a 15-mL Schlenk flask at room temperature under nitrogen atmosphere with LED (455 nm, 10 W) irradiation for 24 h. In addition, the same condition was conducted again, but without XantPhos or Sr_3N_2. GC-MS analysis of the reaction revealed that synergistic catalysis between Sr_3N_2 and XantPhos resulted in a significantly higher yield of **3aa** and the concurrent suppression of side reactions, compared to using either component alone.

3.8 UV-vis Absorption Spectra:

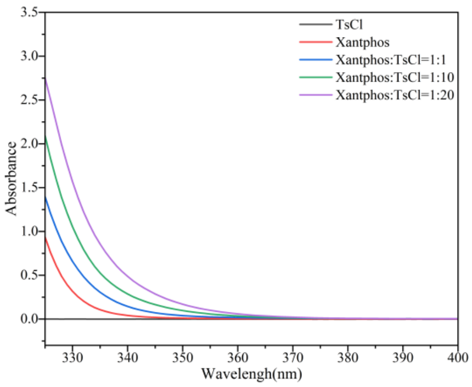

The UV-vis experiments were performed on Shimadzu UV-2600 spectrophotometer with a quartz cuvette (10 mm path length). The UV-vis experiments were carried out by the preparation of HFIP solution containing respectively XantPhos and **2a** in different ratios, keeping constant the amount of XantPhos (1×10^{-3} mol/L) and increasing the amount of **2a** (XantPhos:**2a**=0:1, 1:0, 1:1, 1:10, 1:20).The full spectras were collected, and we observed that after the stoichiometric mixing of XantPhos and **2a** in HFIP, the optical absorption spectrum generated a chemical shift compared to the spectra of the individual components. Moreover, a substantial increase of the absorption was observed with the increase of **2a** loading. These results show pronounced interaction between XantPhos and **2a**.

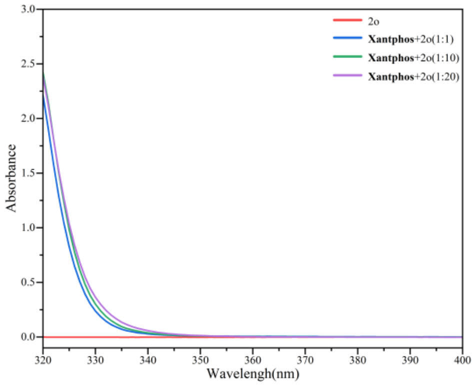

We also measured the UV-Vis absorption spectra of XantPhos and Cyclopropanesulfonyl chloride (**2o**). We found that the visible absorption spectra of the solution with different concentration gradients also had a small chemical shift compared to **2a**. In addition, the absorption peak of the combined solution of XantPhos (1×10^{-3} mol/L) and **2a** or **2o** in the UV-Vis absorption spectrum is not within the wavelength of LED emission at 455 nm. Thus, it strongly supported the existence of noncovalent interactions between **2a** to XantPhos.

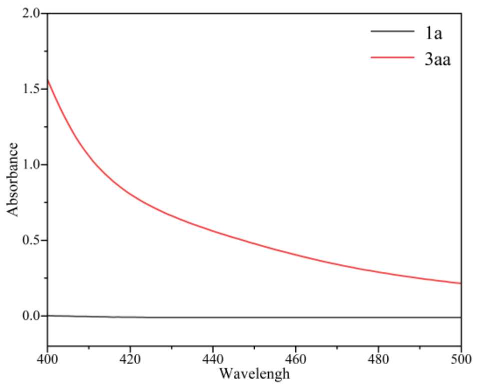

In the UV-vis experiment for measuring the model substrate **1a** and the product **3aa**, we found that the visible light absorption peak of **1a** is not within the wavelength range of 455 nm blue light, while **3aa** has absorption peaks in the range of 400 nm-500 nm. This further proves that **3aa** can be photo-excited to catalyze the reaction during the process.

3.9 NMR Titration Experiments:

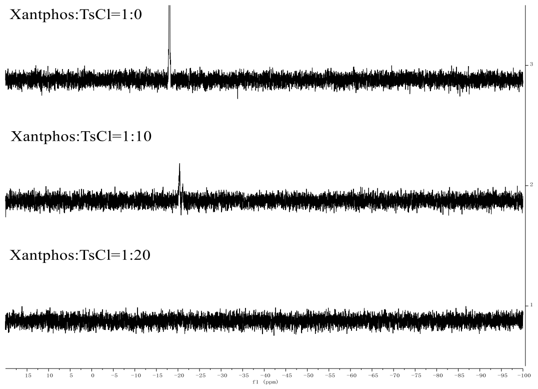

^{31}P NMR titration experiments were carried out by the preparation of $CDCl_3$ solution containing XantPhos and **2a** in different ratios, keeping constant the amount of XantPhos (1×10^{-3} mol/L) and increasing the amount of **2a** (^{31}P-- XantPhos:**2a** = 1:0, 1:10, 1:20).

The complete spectra were collected, the ^{31}P NMR titration experiments showed that as the ratio of **2a** to XantPhos changed, the phosphorus signal of XantPhos shifted to a lower field, which further proved the existence of noncovalent interactions between **2a** and XantPhos.

3.10 Visible ON-OFF Experiments:

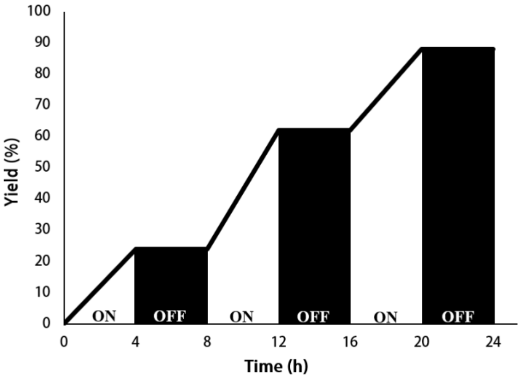

As shown in the figure, the reaction between **1a** and **2a** was conducted under the standard conditions on a 0.1 mmol scale. The mixture was subjected to sequential periods of stirring under a LED (455 nm, 10 W) irradated under nitrogen atmosphere at room temperature with 4 hours and followed by stirring in the absence of light with 4 hours. Compared to the OFF periods, the reaction system was continued during the ON period. The mixture was then purifide with chromatography column on silica gel (PE/EA=15:1) to give the corresponding products **3aa**.

3.11 Reusability and Stability of Sr_3N_2 Experiments:

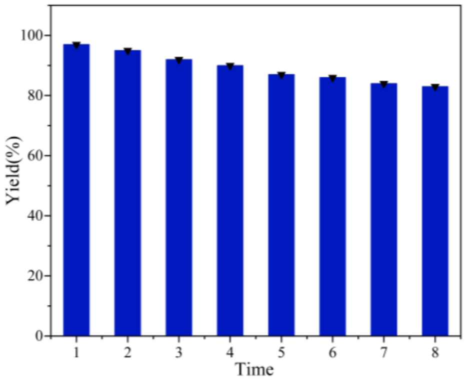

The reusability and stability of Sr_3N_2 was tested: In a typical procedure, to an over-dried 15-mL Schlenk flask equipped with a magnetic stirring bar were added **1a** (0.1 mmol), **2a** (0.2 mmol), Sr_3N_2 (4 mg), XantPhos (0.01 mmol), HFIP (2 mL) and DCE (1 mL).The reaction mixture was stirred at room temperature and under nitrogen atmosphere with LED (455 nm, 10 W) irradiation for 24 h. After completion of the reaction, the photocatalyst was separated by centrifugation and washed twice with the mixed solution, then under vacuum removes the residual solvents, and used for next run.

3.12 The Complete Reaction Profile of Model Reaction with and without Sr_3N_2 and XantPhos:

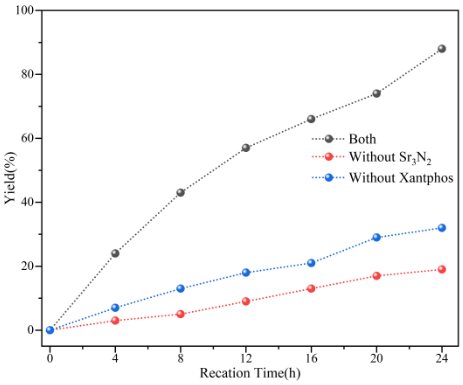

1a (0.2 mmol), **2a** (0.4 mmol), Sr_3N_2 (10 mg), XantPhos (0.02 mmol), HFIP (2 mL) and DCE (1 mL) were in a 15-mL Schlenk flask at room temperature under nitrogen atmosphere with LED (455 nm, 10 W) irradiation for 24 h. In addition, the same condition was conducted again, but without Sr_3N_2 or XantPhos. The processes of these three reactions were monitored by GC. The time profile of model reaction demonstrates that regardless of whether only the photocatalyst or XantPhos was added, it will cause the formation of **3aa** products, but when the two are synergistically catalyzed, one plus one is greater than two in promoting the radical transformation.

3.13 XRD Analysis Experiments:

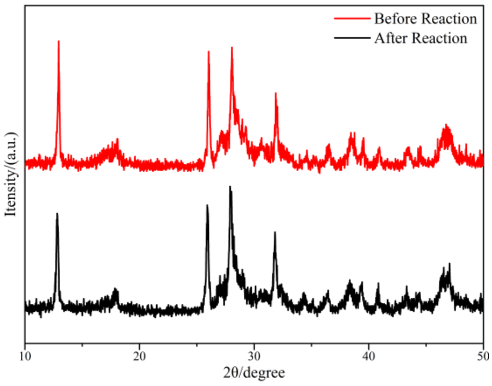

The phase and crystal structure of commercially available strontium nitride powder samples before and after the reaction were characterized by XRD analysis, with a scanning range from 10 °to 50 °, and the results are shown in the figure. The post-reaction strontium nitride powder was alternately washed with acetone (3×10 mL), ethanol (3×10 mL) and deionized water (3×10 mL) followed by centrifugation. Finally, the powder was dried overnight at 40 ℃ under vacuum. XRD characterization analysis confirmed the stability of the semiconductor catalyst, showing that the characteristic peaks remained unchanged before and after the reaction cycles.

3.14 The Job's Plot Analysis Experiments:

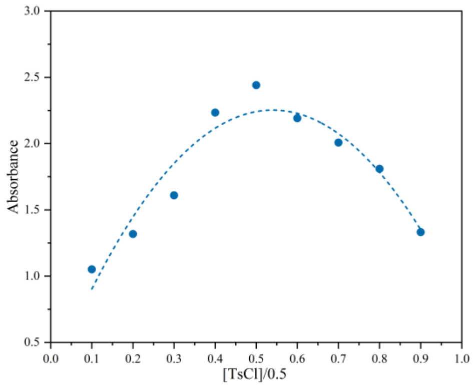

The Job's Plot curve was used to evaluate the binding stoichiometry between **2a** and the halogen bond acceptor XantPhos through experimental analysis by ultraviolet-spectrophotometry. The ultraviolet spectra of 9 samples of the mixture of **2a** and XantPhos in HFIP were recorded at 298 K. In the preparation of the solutions, the total constant concentration [**2a** + XantPhos] was maintained at 1×10^{-3} M. The mole fractions χ (**2a**) of the samples were 0.10, 0.20, 0.30, 0.40, 0.50, 0.60, 0.70, 0.80, and 0.90. Experimental analysis showed that the maximum absorbance was obtained when the ratio of XantPhos: **2a** =1:1, which means that the optimal coordination ratio of XantPhos: **2a** =1:1.

3.15 Determination of Quantum Yield:

$$E_{photon} = \frac{hc}{\lambda_{inc}(455\ nm)} = \frac{6.63 \times 10^{-34}\ J.S \times 3 \times 10^{8}\ m.s^{-1}}{455 \times 10^{-9}\ m} = 4.37 \times 10^{-19}\ J$$

$$E_{total}=P(10W)St = 17.47\times 10^{-3}\ W.cm^{-2} \times 4.78\ cm^{2} \times 24 \times 3600s = 7.21 \times 10^{3}\ J$$

$$\text{Number of incident photons} = \frac{E_{total}}{E_{photon}{*}N_A} = \frac{7.21 \times 10^{3}\ J}{4.37 \times 10^{-19}\ J} = 16.50 \times 10^{21} {*}N_A^{-1} = 27.41\ mmol$$

$$A.Q.Y(\%) = \frac{\text{Number of Product}}{\text{Number of incident photons}} = \frac{0.91\ mmol}{27.41\ mmol} = 3.32\ \% < 1$$

Where h (J·s) is Planck's constant, c ($m\cdot s^{-1}$) is the speed of light and λ_{inc} (m) is the wavelength of the incident light. P ($W\cdot cm^{-2}$) is the power density of the incident light, S (cm^2) is the irradiation area and t (s) is the photoreaction time.

3.16. Computational Details:

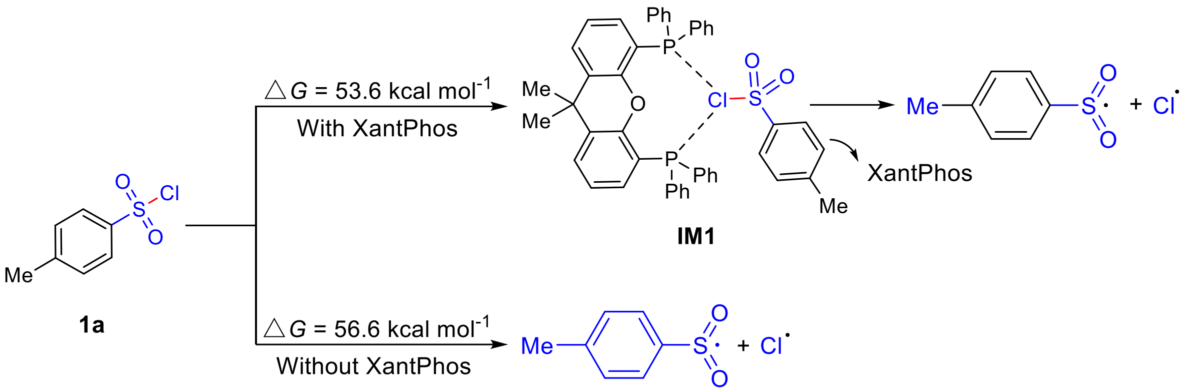

All calculations in this work were performed using the GAUSSIAN 16 quantum chemistry program package[5]. The reactants, transition states, intermediates, and products involved in the theoretical calculations were geometrically optimized using the dispersion-corrected density functional method B3LYP with the 6-31G* basis set. Vibrational frequency calculations were used to confirm whether each stationary point was a stable intermediate or a transition state, and also to obtain the enthalpy and Gibbs free energy corrections for each stationary point. Furthermore, based on the optimized geometries, single-point energy calculations were performed for all stationary points using the M062x functional with the def2-TZVPP basis set[6a] for all atoms. The energies reported in this work are Gibbs free energies obtained using the M062x[6b-d] method in acetonitrile solvent. Solvation calculations employed the SMD solvation model[7-8].

IM1

Zero-point Energy Correction = 0.728608 Hartree
Thermal Correction to Energy = 0.776789 Hartree
Thermal Correction to Enthalpy = 0.777733 Hartree
Thermal Correction to Free Energy = 0.642344 Hartree
EE + Zero-point Energy = -3542.6439 Hartree
EE + Thermal Energy Correction = -3542.5957 Hartree
EE + Thermal Enthalpy Correction = -3542.5948 Hartree
EE + Thermal Free Energy Correction = -3542.7302 Hartree
E(M062X)=- 3543.078161

Symbol	X	Y	Z
C	-4.54241	-1.20778	1.810421
C	-4.05773	-2.20899	0.975393
C	-3.9076	-1.9226	-0.38176
C	-4.24091	-0.68015	-0.91675
C	-4.71696	0.306859	-0.06052
C	-4.84867	0.067523	1.313044
H	-4.65955	-1.40917	2.871644
H	-3.79325	-3.18857	1.356617

H	-4.11524	-0.48459	-1.97503
H	-4.96342	1.282775	-0.46233
C	-5.23742	1.17947	2.24994
H	-4.33885	1.568392	2.746663
H	-5.70788	2.008641	1.714509
H	-5.92209	0.832104	3.031019
S	-3.19569	-3.14892	-1.45117
Cl	-1.18371	-2.44528	-1.51902
O	-3.73467	-2.99975	-2.79968
O	-3.1699	-4.43849	-0.7662
C	1.217891	5.087192	-1.67922
C	0.1921	4.155056	-1.52087
C	0.45562	2.891392	-0.97978
C	1.776789	2.614005	-0.60497
C	2.815553	3.542776	-0.70075
C	2.515183	4.786691	-1.26494
O	2.014436	1.352867	-0.09202
C	3.279701	0.825339	-0.27039
C	3.368067	-0.5698	-0.37187
C	4.6432	-1.11864	-0.56119
C	5.765875	-0.29776	-0.65055
C	5.639695	1.08619	-0.5344
C	4.388999	1.674888	-0.32997
C	4.163064	3.167052	-0.08423
P	-0.78833	1.541073	-0.78472
C	-0.88172	1.43965	1.057085
C	-0.44151	2.452762	1.921205
C	-0.60434	2.32771	3.300668
C	-1.21386	1.190292	3.833012
C	-1.64148	0.172288	2.980949
C	-1.46564	0.290328	1.603474
C	-2.34818	2.474113	-1.14579
C	-2.98328	3.331674	-0.23609
C	-4.18202	3.961408	-0.57127
C	-4.76492	3.7435	-1.82248
C	-4.13928	2.896181	-2.73908
C	-2.94092	2.265638	-2.39961
P	1.801806	-1.55288	-0.3959
C	2.448901	-3.2509	-0.71663
C	2.28234	-3.77122	-2.00703
C	2.753706	-5.0452	-2.32714
C	3.390885	-5.81698	-1.35575
C	3.556589	-5.31114	-0.06395
C	3.089379	-4.03709	0.253799

C	1.383925	-1.69503	1.397451
C	0.457486	-2.68339	1.773115
C	0.09893	-2.84897	3.108519
C	0.640716	-2.01597	4.089764
C	1.538545	-1.01523	3.721981
C	1.911459	-0.85725	2.386757
C	5.311022	4.024655	-0.63088
C	4.055327	3.387267	1.44985
H	1.001546	6.060091	-2.1109
H	-0.81909	4.406025	-1.82174
H	3.293978	5.533395	-1.37257
H	4.752312	-2.19288	-0.6537
H	6.74612	-0.73904	-0.80553
H	6.525682	1.707995	-0.59775
H	0.026304	3.343196	1.512755
H	-0.25844	3.120276	3.959202
H	-1.33767	1.089633	4.907829
H	-2.08678	-0.72914	3.386986
H	-1.77582	-0.51629	0.948681
H	-2.5445	3.49545	0.742644
H	-4.66375	4.62153	0.145394
H	-5.70147	4.229942	-2.08058
H	-4.58689	2.720532	-3.71337
H	-2.46363	1.592501	-3.10762
H	1.769232	-3.1781	-2.75908
H	2.612916	-5.43748	-3.3304
H	3.751369	-6.81228	-1.60066
H	4.049146	-5.91122	0.696535
H	3.215021	-3.64872	1.259636
H	0.032139	-3.33821	1.018724
H	-0.6043	-3.63127	3.382563
H	0.358955	-2.14207	5.131613
H	1.956292	-0.35385	4.47596
H	2.625916	-0.08636	2.121221
H	5.427642	3.899462	-1.71213
H	5.135744	5.083449	-0.42026
H	6.255403	3.760845	-0.14647
H	3.866481	4.44441	1.668029
H	3.234714	2.799179	1.872228
H	4.986061	3.085076	1.942774

Xantphos

Zero-point Energy Correction = 0.597202 Hartree

Thermal Correction to Energy = 0.633057 Hartree

Thermal Correction to Enthalpy = 0.634001 Hartree
Thermal Correction to Free Energy = 0.526644 Hartree
EE + Zero-point Energy = -2262.9733 Hartree
EE + Thermal Energy Correction = -2262.9375 Hartree
EE + Thermal Enthalpy Correction = -2262.9365 Hartree
EE + Thermal Free Energy Correction = -2263.0439 Hartree
E(M062X)= -2263.291420

Symbol	X	Y	Z
C	3.679145	3.067073	-0.18929
C	3.580356	1.675086	-0.21322
C	2.332962	1.047404	-0.16115
C	1.190814	1.867752	-0.07654
C	1.255308	3.26134	-0.06026
C	2.528798	3.844285	-0.11828
O	-6E-06	1.187973	0.000369
C	-1.19079	1.867832	0.076974
C	-2.333	1.047573	0.161463
C	-3.58036	1.675365	0.213558
C	-3.67904	3.067348	0.18973
C	-2.52862	3.844474	0.118739
C	-1.25519	3.261431	0.060701
C	0.000085	4.13081	0.000034
P	2.102974	-0.77283	-0.3933
C	3.850194	-1.35499	-0.23505
C	4.542127	-1.65403	-1.41712
C	5.86954	-2.0834	-1.3775
C	6.516991	-2.23156	-0.15059
C	5.834032	-1.946	1.034167
C	4.511101	-1.50853	0.992589
C	1.421545	-1.31432	1.2327
C	1.051858	-2.66312	1.341005
C	0.484211	-3.15478	2.513983
C	0.267786	-2.30005	3.596729
C	0.639377	-0.95932	3.502171
C	1.217736	-0.47039	2.330315
P	-2.10329	-0.77268	0.393608
C	-3.85051	-1.35475	0.234723
C	-4.54247	-1.6553	1.416355
C	-5.8698	-2.08496	1.376099
C	-6.51711	-2.23177	0.148989
C	-5.83412	-1.9446	-1.0354
C	-4.5113	-1.50695	-0.99318
C	-1.42149	-1.3142	-1.23225
C	-1.05175	-2.66301	-1.34043

C	-0.48398	-3.15471	-2.51331
C	-0.26747	-2.30003	-3.5961
C	-0.63907	-0.95932	-3.50166
C	-1.21758	-0.47033	-2.32987
C	0.057636	5.022894	1.263579
C	-0.05742	5.022174	-1.26406
H	4.654822	3.542508	-0.22755
H	4.478286	1.071106	-0.2757
H	2.616522	4.926456	-0.10828
H	-4.47833	1.071439	0.275912
H	-4.65467	3.542865	0.228046
H	-2.61627	4.926651	0.108754
H	4.03259	-1.55141	-2.37207
H	6.393922	-2.30958	-2.30199
H	7.548008	-2.57288	-0.11613
H	6.334357	-2.06317	1.991834
H	3.983377	-1.28728	1.915145
H	1.201617	-3.32656	0.493535
H	0.201975	-4.20215	2.580954
H	-0.18686	-2.67805	4.508271
H	0.478888	-0.28925	4.342665
H	1.507398	0.573455	2.269294
H	-4.03307	-1.55363	2.371471
H	-6.39418	-2.31237	2.300291
H	-7.54805	-2.57327	0.114017
H	-6.33436	-2.06071	-1.99323
H	-3.98351	-1.28454	-1.91543
H	-1.20152	-3.32635	-0.49289
H	-0.2017	-4.20208	-2.58022
H	0.187286	-2.6781	-4.50756
H	-0.47851	-0.28927	-4.34216
H	-1.5072	0.573532	-2.26894
H	0.098263	4.406039	2.166506
H	0.942767	5.666783	1.247914
H	-0.82507	5.666964	1.328347
H	0.825348	5.666127	-1.32919
H	-0.09809	4.404777	-2.16661
H	-0.94253	5.666102	-1.24875

Cl-radidal

Zero-point Energy Correction = 0 Hartree

Thermal Correction to Energy = 0.001416 Hartree

Thermal Correction to Enthalpy = 0.00236 Hartree

Thermal Correction to Free Energy = -0.015677 Hartree

EE + Zero-point Energy = -460.13624 Hartree
EE + Thermal Energy Correction = -460.13483 Hartree
EE + Thermal Enthalpy Correction = -460.13388 Hartree
EE + Thermal Free Energy Correction = -460.15192 Hartree
E(M062X)= -460.132636

Symbol	X	Y	Z
Cl	0.0000000	0.0000000	0.0000000

IM2

Zero-point Energy Correction = 0.126461 Hartree
Thermal Correction to Energy = 0.135957 Hartree
Thermal Correction to Enthalpy = 0.136901 Hartree
Thermal Correction to Free Energy = 0.089564 Hartree
EE + Zero-point Energy = -819.42293 Hartree
EE + Thermal Energy Correction = -819.41344 Hartree
EE + Thermal Enthalpy Correction = -819.41249 Hartree
EE + Thermal Free Energy Correction = -819.45983 Hartree
E(M062X)=-819.526868

Symbol	X	Y	Z
C	-1.70364	1.205856	-0.01941
C	-0.31286	1.218271	-0.07918
C	0.364889	-6E-06	-0.09188
C	-0.31291	-1.21829	-0.0792
C	-1.70365	-1.20584	-0.01944
C	-2.41823	0.000034	0.012808
H	-2.24444	2.148181	0.004047
H	0.241948	2.149708	-0.09698
H	0.241981	-2.14968	-0.09707
H	-2.24453	-2.14811	0.004047
C	-3.92304	-5E-06	0.106475
H	-4.35367	-0.88704	-0.36839
H	-4.24767	-3.6E-05	1.155153
H	-4.35372	0.887032	-0.36833
S	2.163688	-1.4E-05	-0.24157
O	2.649835	-1.30368	0.290753
O	2.649876	1.303683	0.29069

TsCl

Zero-point Energy Correction = 0.129518 Hartree
Thermal Correction to Energy = 0.140321 Hartree
Thermal Correction to Enthalpy = 0.141265 Hartree
Thermal Correction to Free Energy = 0.09075 Hartree
EE + Zero-point Energy = -1279.6455 Hartree
EE + Thermal Energy Correction = -1279.6347 Hartree

EE + Thermal Enthalpy Correction = -1279.6337 Hartree

EE + Thermal Free Energy Correction = -1279.6842 Hartree

E(M062X)= -1279.766504

Symbol	X	Y	Z
C	-2.15611	1.206755	-0.01377
C	-0.77562	1.220223	-0.18559
C	-0.10681	-5.4E-05	-0.27017
C	-0.77564	-1.2203	-0.18521
C	-2.15613	-1.20675	-0.01339
C	-2.86422	0.000024	0.079873
H	-2.69443	2.148521	0.044986
H	-0.22547	2.150903	-0.26514
H	-0.22552	-2.15102	-0.26447
H	-2.69448	-2.14848	0.04568
C	-4.35533	0.000068	0.30044
H	-4.82445	-0.88689	-0.13613
H	-4.58799	-0.00034	1.37318
H	-4.8243	0.887446	-0.13544
S	1.65533	-0.00012	-0.49718
Cl	2.326087	0.000351	1.529389
O	2.074188	1.276623	-1.06159
O	2.074188	-1.27713	-1.06098

4. Characterization Data of Products

9-phenyl-10-(o-tolylsulfonyl) phenanthrene (3ab):

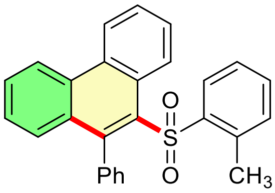

^{1}H NMR (400 MHz, Chloroform-d) δ 9.11 (d, J = 8.6 Hz, 1H), 8.77 (t, J = 8.1 Hz, 2H), 7.73 (dt, J = 14.8, 7.3 Hz, 2H), 7.61 (t, J = 7.8 Hz, 1H), 7.49 (dd, J = 13.4, 7.8 Hz, 2H), 7.37 (dd, J = 15.1, 7.8 Hz, 2H), 7.31 – 7.26 (m, 3H), 7.16 (d, J = 7.3 Hz, 2H), 7.04 (t, J = 7.4 Hz, 2H), 2.13 (s, 3H).

^{13}C NMR (101 MHz, Chloroform-d) δ 142.60, 132.20, 132.08, 130.84, 129.52, 129.36, 127.98, 127.63, 127.57, 127.47, 127.34, 127.30, 126.16, 122.99, 122.63, 77.48, 76.84, 19.86, 1.17.

HRMS (ESI) calculated for $C_{27}H_{21}O_2S$ $[M+H]^+$ m/z 408.1184, found 408.1186.

9-phenyl-10-(m-tolylsulfonyl) phenanthrene (3ac):

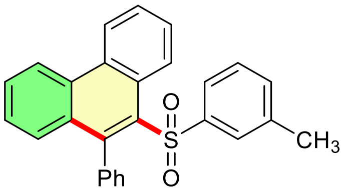

^{1}H NMR (400 MHz, Chloroform-d) δ 9.14 (d, J = 8.5 Hz, 1H), 8.85 – 8.80 (m, 2H), 7.83 (t, J = 7.6 Hz, 1H), 7.77 (t, J = 7.6 Hz, 1H), 7.70 (t, J = 7.8 Hz, 1H), 7.55 (t, J = 7.5 Hz, 2H), 7.49 (d, J = 7.6 Hz, 4H), 7.45 (d, J = 8.6 Hz, 1H), 7.34 (d, J = 6.7 Hz, 2H), 7.29 (d, J = 6.6 Hz, 2H), 2.36 (s, 3H).

^{13}C NMR (101 MHz, Chloroform-d) δ 138.99, 137.50, 133.40, 130.36, 129.70, 129.64, 128.75, 127.64, 127.52, 127.32, 127.20, 127.02, 126.82, 123.63, 123.02, 122.54, 77.48, 76.84, 21.43.

HRMS (ESI) calculated for $C_{27}H_{21}O_2S$ $[M+H]^+$ m/z 408.1184, found 408.1188.

9-phenyl-10-(phenylsulfonyl) phenanthrene (3ad)[2]:

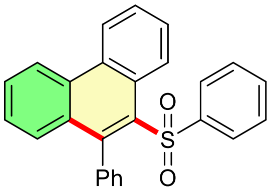

^{1}H NMR (400 MHz, Chloroform-d) δ 9.02 (d, J = 8.6 Hz, 1H), 8.75 (t, J = 8.0 Hz, 2H), 7.78 – 7.74 (m, 1H), 7.68 (d, J = 8.0 Hz, 3H), 7.60 (t, J = 7.8 Hz, 1H), 7.46 (dd, J = 14.0, 7.1 Hz, 5H), 7.41 (d, J = 7.1 Hz, 1H), 7.38 – 7.33 (m, 2H), 7.29 (d, J = 7.3 Hz, 2H).

^{13}C NMR (101 MHz, Chloroform-d) δ 143.99, 143.83, 137.62, 132.52, 132.36, 131.75, 130.96, 130.25, 129.75, 129.70, 128.90, 127.72, 127.54, 127.35, 127.16, 126.97, 126.42, 123.05, 122.54.

9-([1,1'-biphenyl]-4-ylsulfonyl)-10-phenylphenanthrene (3ae):

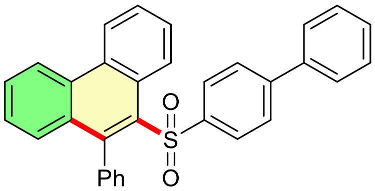

^{1}H NMR (400 MHz, Chloroform-d) δ 9.12 (d, J = 8.4 Hz, 1H), 8.76 (t, J = 9.3 Hz, 2H), 7.78 – 7.69 (m, 4H), 7.67 – 7.63 (m, 1H), 7.51 (t, J = 9.5 Hz, 5H), 7.47 – 7.42 (m, 5H), 7.39 (dd, J = 7.6, 5.2 Hz, 2H), 7.31 – 7.28

(m, 2H).
^{13}C NMR (101 MHz, Chloroform-d) δ 130.38, 129.73, 129.70, 129.13, 128.55, 127.77, 127.74, 127.62, 127.59, 127.54, 127.38, 127.23, 127.08, 123.09, 122.57, 77.48, 76.84.
HRMS (ESI) calculated for $C_{32}H_{23}O_2S$ $[M+H]^+$ m/z 470.1341, found 470.1345.
9-phenyl-10-tosylphenanthrene (3aa):

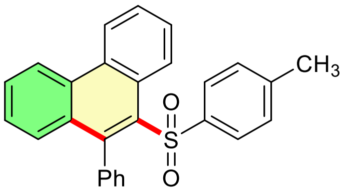

^{1}H NMR (500 MHz, Chloroform-d) δ 9.07 – 9.01 (m, 1H), 8.74 (t, J = 9.2 Hz, 2H), 7.74 (ddd, J = 8.3, 6.9, 1.3 Hz, 1H), 7.70 – 7.66 (m, 1H), 7.62 – 7.57 (m, 3H), 7.50 – 7.43 (m, 4H), 7.40 – 7.37 (m, 1H), 7.30 (dd, J = 7.7, 1.7 Hz, 2H), 7.13 (d, J = 8.0 Hz, 2H), 2.33 (s, 3H).
^{13}C NMR (101 MHz, Chloroform-d) δ 130.18, 129.80, 129.62, 129.53, 127.69, 127.52, 127.48, 127.32, 127.26, 126.58, 123.03, 122.53, 77.48, 76.84, 21.65.
HRMS (ESI) calculated for $C_{27}H_{21}O_2S$ $[M+H]^+$ m/z 408.1136, found 408.1143.
9-((4-(tert-butyl)phenyl)sulfonyl)-10-phenylphenanthrene (3af):

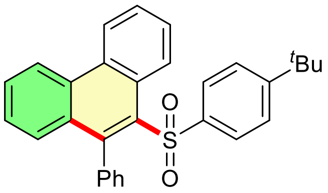

^{1}H NMR (400 MHz, Chloroform-d) δ 9.11 (d, J = 8.6 Hz, 1H), 8.78 – 8.72 (m, 2H), 7.72 (dt, J = 15.2, 7.2 Hz, 2H), 7.65 (d, J = 7.3 Hz, 1H), 7.59 (d, J = 8.5 Hz, 2H), 7.50 – 7.43 (m, 2H), 7.42 (d, J = 7.4 Hz, 3H), 7.37 – 7.32 (m, 3H), 7.28 (s, 1H), 1.26 (s, 9H).
^{13}C NMR (101 MHz, Chloroform-d) δ 156.22, 140.77, 137.71, 132.30, 131.79, 130.98, 130.32, 129.70, 129.57, 128.62, 127.66, 127.64, 127.53, 127.48, 127.34, 127.28, 127.09, 126.41, 125.91, 123.00, 122.52, 35.17, 31.14.
HRMS (ESI) calculated for $C_{30}H_{27}O_2S$ $[M+H]^+$ m/z 450.1654, found 450.1657.
9-((4-methoxyphenyl)sulfonyl)-10-phenylphenanthrene (3ag):

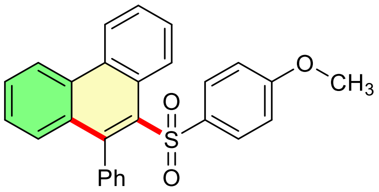

^{1}H NMR (400 MHz, Chloroform-d) δ 9.09 (d, J = 8.5 Hz, 1H), 8.74 (t, J = 9.1 Hz, 2H), 7.76 – 7.71 (m, 1H), 7.71 – 7.67 (m, 1H), 7.65 – 7.61 (m, 3H), 7.49 – 7.43 (m, 4H), 7.36 (d, J = 8.4 Hz, 1H), 7.29 (dd, J = 6.1, 2.5 Hz, 2H), 6.79 (d, J = 8.9 Hz, 2H), 3.78 (s, 3H).
^{13}C NMR (101 MHz, Chloroform-d) δ 162.75, 143.40, 137.94, 135.47, 133.32, 132.27, 131.85, 131.00, 130.20, 129.74, 129.55, 128.88, 127.70, 127.50, 127.46, 127.29, 127.24, 126.98, 123.03, 122.51, 114.09, 55.68.
HRMS (ESI) calculated for $C_{27}H_{21}O_2S$ $[M+H]^+$ m/z 424.1133, found 424.1139.

9-((4-fluorophenyl)sulfonyl)-10-phenylphenanthrene (3ah):

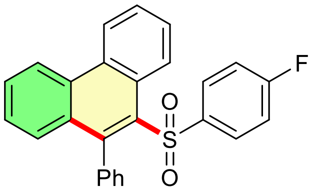

^{1}H NMR (400 MHz, Chloroform-d) δ 9.12 (d, J = 8.6 Hz, 1H), 8.86 – 8.79 (m, 2H), 7.81 (dt, J = 17.7, 7.4 Hz, 2H), 7.71 (dt, J = 8.2, 4.5 Hz, 3H), 7.52 (dd, J = 14.1, 7.6 Hz, 4H), 7.44 (d, J = 8.4 Hz, 1H), 7.06 (t, J = 8.6 Hz, 2H).

^{13}C NMR (101 MHz, Chloroform-d) δ 139.88, 137.48, 132.37, 131.01, 130.40, 129.80, 129.71, 129.39, 129.29, 127.90, 127.78, 127.64, 127.62, 127.42, 127.03, 126.87, 123.14, 122.58, 116.24, 116.01.

^{19}F NMR (376 MHz, Chloroform-d) δ -105.23 (tt, J = 8.8, 5.1 Hz).

HRMS (ESI) calculated for $C_{26}H_{18}FO_2S$ $[M+H]^+$ m/z 412.0933, found 412.0941.

9-((4-chlorophenyl)sulfonyl)-10-phenylphenanthrene (3ai):

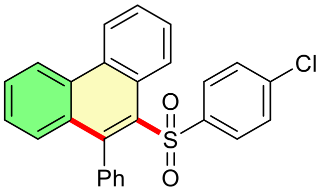

^{1}H NMR (400 MHz, Chloroform-d) δ 9.04 (d, J = 8.6 Hz, 1H), 8.76 (t, J = 9.8 Hz, 2H), 7.75 (dd, J = 16.4, 8.9 Hz, 2H), 7.66 – 7.62 (m, 1H), 7.56 (d, J = 8.2 Hz, 2H), 7.44 (dt, J = 17.5, 6.8 Hz, 5H), 7.37 (d, J = 8.1 Hz, 1H), 7.29 (s, 1H), 7.23 (s, 1H), 7.16 (s, 1H).

^{13}C NMR (101 MHz, Chloroform-d) δ 142.30, 138.95, 137.37, 132.38, 131.60, 130.99, 130.42, 129.84, 129.70, 129.13, 128.61, 128.01, 127.92, 127.77, 127.66, 127.43, 126.99, 126.25, 123.15, 122.58.

HRMS (ESI) calculated for $C_{26}H_{18}ClO_2S$ $[M+H]^+$ m/z 428.0638, found 428.0651.

9-((4-bromophenyl)sulfonyl)-10-phenylphenanthrene (3aj):

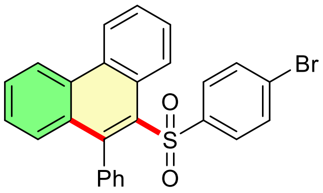

^{1}H NMR (400 MHz, Chloroform-d) δ 9.03 (d, J = 8.6 Hz, 1H), 8.76 (dd, J = 11.2, 8.6 Hz, 2H), 7.73 (dd, J = 17.3, 8.3 Hz, 2H), 7.63 (d, J = 7.8 Hz, 1H), 7.47 (s, 3H), 7.45 – 7.42 (m, 4H), 7.38 (t, J = 8.6 Hz, 2H), 7.24 (d, J = 7.4 Hz, 2H).

^{13}C NMR (101 MHz, Chloroform-d) δ 143.88, 142.86, 142.86, 137.36, 132.54, 132.54, 132.41, 132.12, 131.61, 131.00, 130.45, 129.86, 129.72, 128.10, 127.94, 127.79, 127.69, 127.45, 127.00, 126.84, 126.84, 123.17, 122.60, 117.96.

HRMS (ESI) calculated for $C_{26}H_{18}BrO_2S$ $[M+H]^+$ m/z 472.0133, found 472.0142.

4-((10-phenylphenanthren-9-yl)sulfonyl)benzonitrile (3ak):

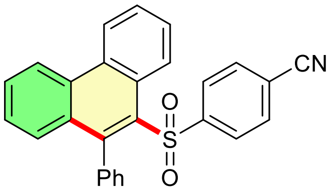

^{1}H NMR (400 MHz, Chloroform-d) δ 9.01 (d, J = 8.6 Hz, 1H), 8.77 (dd, J = 13.1, 8.4 Hz, 2H), 7.80 – 7.72 (m, 2H), 7.67 (t, J = 7.4 Hz, 3H), 7.58 (d, J = 8.4 Hz, 2H), 7.51 – 7.45 (m, 2H), 7.41 – 7.36 (m, 3H), 7.21 (d, J = 7.1 Hz, 2H).

^{13}C NMR (101 MHz, Chloroform-d) δ 148.01, 144.25, 136.89, 132.63, 132.54, 131.41, 131.41, 130.76, 130.16, 129.68, 128.20, 128.00, 127.89, 127.81, 127.62, 127.07, 126.76, 123.30, 122.68, 77.48, 76.84, 1.17.

HRMS (ESI) calculated for $C_{27}H_{18}NO_2S$ $[M+H]^+$ m/z 419.0980, found 419.0992.

methyl 4-((10-phenylphenanthren-9-yl)sulfonyl)benzoate (3al):

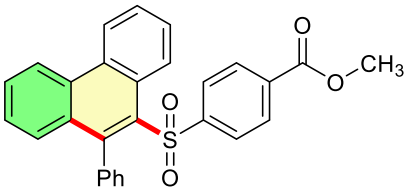

^{1}H NMR (400 MHz, Chloroform-d) δ 8.97 (d, J = 8.6 Hz, 1H), 8.76 (t, J = 8.2 Hz, 2H), 7.97 (d, J = 8.4 Hz, 2H), 7.77 (t, J = 7.6 Hz, 1H), 7.70 (t, J = 7.4 Hz, 3H), 7.61 (t, J = 7.8 Hz, 1H), 7.49 (dd, J = 14.1, 6.9 Hz, 2H), 7.45 – 7.36 (m, 4H), 7.27 (s, 1H), 3.91 (s, 3H).

^{13}C NMR (101 MHz, Chloroform-d) δ 133.48, 130.43, 130.10, 129.96, 129.76, 127.99, 127.81, 127.70, 127.49, 126.92, 126.42, 123.18, 122.62, 77.48, 76.84, 52.78.

HRMS (ESI) calculated for $C_{28}H_{21}O_4S$ $[M+H]^+$ m/z 452.1082, found 452.1095.

3-((10-phenylphenanthren-9-yl)sulfonyl)thiophene (3am):

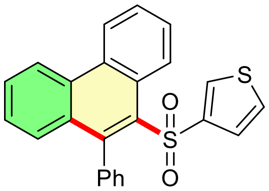

^{1}H NMR (400 MHz, Chloroform-d) δ 9.20 (d, J = 8.2 Hz, 1H), 8.76 (dd, J = 15.4, 8.3 Hz, 2H), 7.82 – 7.79 (m, 1H), 7.77 – 7.67 (m, 3H), 7.50 – 7.46 (m, 4H), 7.37 (d, J = 8.3 Hz, 1H), 7.28 (dd, J = 7.2, 4.0 Hz, 2H), 7.21 (dd, J = 4.9, 2.8 Hz, 1H), 7.18 – 7.15 (m, 1H).

^{13}C NMR (101 MHz, Chloroform-d) δ 144.03, 137.75, 133.37, 132.34, 131.78, 131.03, 130.64, 130.13, 129.84, 129.73, 127.82, 127.72, 127.60, 127.43, 127.35, 127.05, 126.93, 125.45, 123.13, 122.54.

HRMS (ESI) calculated for $C_{24}H_{17}O_2S_2$ $[M+H]^+$ m/z 400.0592, found 400.0596.

9-(cyclohexylsulfonyl)-10-phenylphenanthrene (3an):

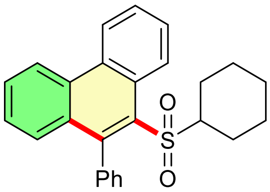

^{1}H NMR (400 MHz, Chloroform-d) δ 9.13 (dd, J = 7.0, 2.2 Hz, 1H), 8.85 – 8.74 (m, 2H), 7.76 (dt, J = 8.2, 5.0 Hz, 3H), 7.50 – 7.47 (m, 3H), 7.37 – 7.30 (m, 3H), 7.25 (s, 1H), 1.86 (d, J = 11.2 Hz, 2H), 1.78 (s, 2H),

1.58 – 1.57 (m, 1H), 1.52 (s, 1H), 1.26 (s, 2H), 1.11 (d, J = 7.8 Hz, 2H).

^{13}C NMR (101 MHz, Chloroform-d) δ 129.92, 129.64, 127.94, 127.64, 127.46, 127.41, 126.84, 123.47, 122.51, 77.48, 76.84, 62.93, 25.19, 24.69, 1.18

HRMS (ESI) calculated for $C_{26}H_{25}O_2S$ $[M+H]^+$ m/z 400.1497, found 400.1502.

9-(cyclopropylsulfonyl)-10-phenylphenanthrene (3ao):

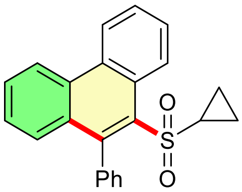

^{1}H NMR (400 MHz, Chloroform-d) δ 9.29 – 9.21 (m, 1H), 8.86 – 8.74 (m, 2H), 7.80 – 7.73 (m, 3H), 7.51 – 7.47 (m, 4H), 7.39 – 7.33 (m, 3H), 1.23 (dd, J = 4.7, 2.1 Hz, 2H), 0.86 (dd, J = 7.9, 2.1 Hz, 2H).

^{13}C NMR (101 MHz, Chloroform-d) δ 131.30, 130.10, 129.95, 129.53, 127.86, 127.63, 127.40, 127.00, 123.42, 122.54, 77.48, 76.84, 34.24, 6.35, 1.18.

HRMS (ESI) calculated for $C_{23}H_{19}O_2S$ $[M+H]^+$ m/z 358.1028, found 358.1033.

9-(butylsulfonyl)-10-phenylphenanthrene (3ap):

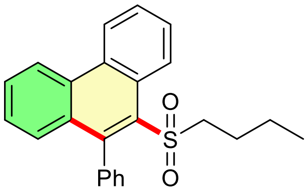

^{1}H NMR (400 MHz, Chloroform-d) δ 9.10 (dd, J = 6.9, 2.6 Hz, 1H), 8.84 (dd, J = 6.7, 2.6 Hz, 1H), 8.77 (d, J = 8.4 Hz, 1H), 7.79 – 7.75 (m, 3H), 7.53 – 7.49 (m, 4H), 7.39 (d, J = 8.3 Hz, 1H), 7.34 – 7.31 (m, 2H), 3.28 – 3.23 (m, 2H), 1.66 – 1.61 (m, 2H), 1.26 (s, 2H), 0.80 (t, J = 7.3 Hz, 3H).

^{13}C NMR (101 MHz, Chloroform-d) δ 138.04, 138.04, 132.21, 131.84, 131.19, 131.19, 129.97, 129.80, 129.73, 128.64, 128.02, 127.77, 127.68, 127.44, 126.59, 123.47, 122.55, 56.85, 24.21, 21.64, 13.59.

HRMS (ESI) calculated for $C_{24}H_{23}O_2S$ $[M+H]^+$ m/z 374.1341, found 374.1348.

9-(o-tolyl)-10-tosylphenanthrene (3ba):

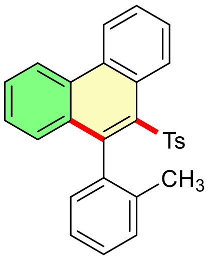

^{1}H NMR (500 MHz, Chloroform-d) δ 9.03 (d, J = 8.6 Hz, 1H), 8.75 (dd, J = 11.3, 8.6 Hz, 2H), 7.77 – 7.73 (m, 1H), 7.67 (dd, J = 16.0, 8.1 Hz, 3H), 7.60 (t, J = 7.8 Hz, 1H), 7.48 (t, J = 7.6 Hz, 1H), 7.40 (t, J = 7.3 Hz, 1H), 7.34 – 7.26 (m, 3H), 7.16 (d, J = 8.1 Hz, 2H), 7.12 (d, J = 7.4 Hz, 1H), 2.34 (s, 3H), 2.05 (s, 3H).

^{13}C NMR (126 MHz, Chloroform-d) δ 143.59, 143.44, 140.96, 137.75, 137.33, 132.50, 131.11, 131.06, 129.72, 129.66, 129.52, 129.38, 129.09, 128.08, 127.60, 127.47, 127.41, 127.19, 126.60, 125.29, 123.04, 122.65, 77.41, 76.91, 21.60, 20.45.

HRMS (ESI) calculated for $C_{28}H_{23}O_2S$ $[M+H]^+$ m/z 422.1341, found 422.1344.

9-(m-tolyl)-10-tosylphenanthrene (3ca):

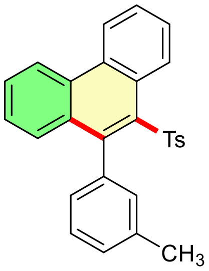

^{1}H NMR (500 MHz, Chloroform-d) δ 9.13 (d, J = 8.5 Hz, 1H), 8.73 (dd, J = 14.1, 8.3 Hz, 2H), 7.75 – 7.71 (m, 1H), 7.70 – 7.66 (m, 1H), 7.65 – 7.61 (m, 1H), 7.54 (d, J = 8.3 Hz, 2H), 7.47 (t, J = 7.6 Hz, 1H), 7.39 (d, J = 8.4 Hz, 1H), 7.33 (t, J = 7.6 Hz, 1H), 7.24 (d, J = 7.6 Hz, 1H), 7.12 (t, J = 8.3 Hz, 3H), 6.96 (s, 1H), 2.35 (s, 3H), 2.33 (s, 3H).

^{13}C NMR (126 MHz, Chloroform-d) δ 143.71, 143.16, 141.13, 137.49, 137.03, 133.27, 132.30, 131.88, 131.10, 130.95, 129.78, 129.51, 129.38, 128.47, 127.73, 127.54, 127.45, 127.41, 127.32, 127.24, 127.20, 126.71, 122.99, 122.49, 77.41, 76.91, 21.59.

HRMS (ESI) calculated for $C_{28}H_{23}O_2S$ $[M+H]^+$ m/z 422.1341, found 422.1343.

9-([1,1'-biphenyl]-4-yl)-10-tosylphenanthrene (3da):

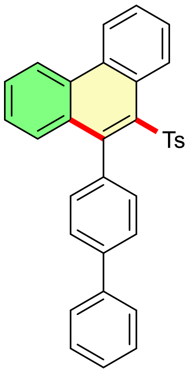

^{1}H NMR (400 MHz, Chloroform-d) δ 9.09 (d, J = 8.6 Hz, 1H), 8.76 (t, J = 8.0 Hz, 2H), 7.78 – 7.74 (m, 1H), 7.71 (t, J = 8.0 Hz, 3H), 7.64 (d, J = 7.9 Hz, 3H), 7.56 (d, J = 8.1 Hz, 2H), 7.51 (d, J = 7.6 Hz, 2H), 7.48 – 7.45 (m, 2H), 7.39 (s, 1H), 7.33 (d, J = 8.0 Hz, 2H), 7.11 (d, J = 8.0 Hz, 2H), 2.34 (s, 3H).

^{13}C NMR (101 MHz, Chloroform-d) δ 130.81, 129.71, 129.48, 128.97, 127.58, 127.54, 127.37, 127.27, 127.25, 126.67, 126.32, 123.04, 122.59, 77.48, 76.84, 21.70, 1.17.

HRMS (ESI) calculated for $C_{33}H_{25}O_2S$ $[M+H]^+$ m/z 484.1497, found 484.1506.

9-(p-tolyl)-10-tosylphenanthrene (3ea):

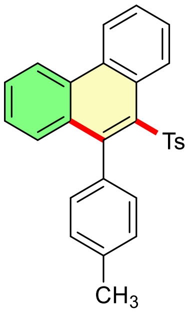

^{1}H NMR (500 MHz, Chloroform-d) δ 9.03 (d, J = 8.5 Hz, 1H), 8.73 (t, J = 9.1 Hz, 2H), 7.75 – 7.71 (m, 1H), 7.67 (t, J = 7.6 Hz, 1H), 7.58 (dd, J = 18.3, 8.3 Hz, 3H), 7.49 – 7.42 (m, 2H), 7.24 (d, J = 7.8 Hz, 2H), 7.17

(d, J = 7.9 Hz, 2H), 7.12 (d, J = 8.1 Hz, 2H), 2.47 (s, 3H), 2.33 (s, 3H).

^{13}C NMR (126 MHz, Chloroform-d) δ 143.50, 140.80, 137.02, 133.57, 133.12, 130.68, 130.57, 130.24, 130.02, 129.56, 128.68, 128.06, 127.90, 127.86, 127.77, 127.45, 127.04, 126.66, 124.24, 122.96, 77.41, 76.91, 21.61, 1.15.

HRMS (ESI) calculated for $C_{28}H_{23}O_2S$ $[M+H]^+$ m/z 422.1341, found 422.1354.

9-(4-methoxyphenyl)-10-tosylphenanthrene (3fa):

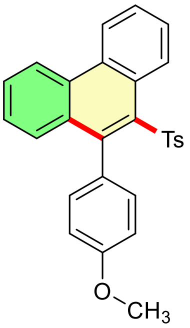

^{1}H NMR (400 MHz, Chloroform-d) δ 9.08 (d, J = 8.6 Hz, 1H), 8.73 (t, J = 8.1 Hz, 2H), 7.76 – 7.66 (m, 2H), 7.63 – 7.59 (m, 1H), 7.54 – 7.47 (m, 3H), 7.46 – 7.44 (m, 1H), 7.13 (dd, J = 18.6, 8.4 Hz, 4H), 6.93 (d, J = 8.6 Hz, 2H), 3.90 (s, 3H), 2.33 (s, 3H).

^{13}C NMR (101 MHz, Chloroform-d) δ 143.17, 143.17, 131.59, 131.59, 129.41, 127.43, 127.30, 127.30, 127.27, 126.56, 122.99, 122.54, 113.22, 77.48, 76.84, 55.43, 21.65, 1.17.

HRMS (ESI) calculated for $C_{28}H_{23}O_3S$ $[M+H]^+$ m/z 438.1290, found 438.1301.

9-(4-fluorophenyl)-10-tosylphenanthrene (3ga):

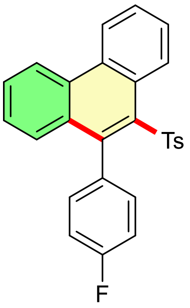

^{1}H NMR (500 MHz, Chloroform-d) δ 9.07 (d, J = 8.6 Hz, 1H), 8.74 (t, J = 8.3 Hz, 2H), 7.75 (t, J = 7.6 Hz, 1H), 7.69 (t, J = 7.6 Hz, 1H), 7.62 (t, J = 7.8 Hz, 1H), 7.56 (d, J = 8.2 Hz, 2H), 7.50 (t, J = 7.6 Hz, 1H), 7.37 (d, J = 8.4 Hz, 1H), 7.24 (dd, J = 8.4, 5.4 Hz, 2H), 7.16 – 7.10 (m, 4H), 2.34 (s, 3H).

^{13}C NMR (126 MHz, Chloroform-d) δ 143.50, 142.63, 141.02, 133.56(d, J=3.03),133.46, 132.39, 131.92(d, J=6.06), 131.80, 131.05, 129.71, 129.54, 129.45, 127.60(d, J=6.06), 127.35(d, J=12.12), 127.04, 126.51, 123.05, 122.65, 114.91, 114.74, 21.61.

^{19}F NMR (471 MHz, Chloroform-d) δ -114.04, -114.25 (tt, J = 9.3, 5.2 Hz), -115.13, -115.14.

HRMS (ESI) calculated for $C_{27}H_{20}FO_2S$ $[M+H]^+$ m/z 426.1090, found 426.1102.

9-(4-chlorophenyl)-10-tosylphenanthrene (3ha):

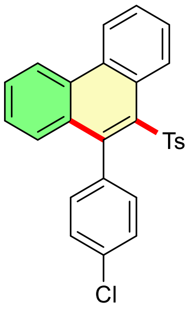

^{1}H NMR (500 MHz, Chloroform-d) δ 9.05 (d, J = 8.6 Hz, 1H), 8.76 – 8.71 (m, 2H), 7.75 (t, J = 7.7 Hz, 1H), 7.69 (t, J = 7.3 Hz, 1H), 7.61 (t, J = 7.8 Hz, 1H), 7.57 (d, J = 8.3 Hz, 2H), 7.50 (t, J = 7.7 Hz, 1H), 7.41 (d, J = 8.3 Hz, 2H), 7.36 (d, J = 8.4 Hz, 1H), 7.22 (d, J = 8.3 Hz, 2H), 7.15 (d, J = 8.1 Hz, 2H), 2.34 (s, 3H).
^{13}C NMR (126 MHz, Chloroform-d) δ 143.49, 142.20, 140.77, 136.22, 133.77, 133.22, 132.27, 131.44, 131.40, 130.97, 129.64, 129.45, 129.33, 127.88, 127.57, 127.50, 127.34, 127.16, 126.83, 126.45, 122.95, 122.55, 21.51.
HRMS (ESI) calculated for $C_{27}H_{20}ClO_2S$ $[M+H]^+$ m/z 442.0794, found 442.0813.
9-(4-bromophenyl)-10-tosylphenanthrene (3ia):

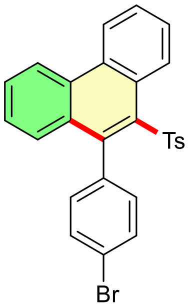

^{1}H NMR (500 MHz, Chloroform-d) δ 9.05 (d, J = 8.6 Hz, 1H), 8.74 (t, J = 7.9 Hz, 2H), 7.75 (t, J = 7.6 Hz, 1H), 7.69 (t, J = 7.5 Hz, 1H), 7.61 (t, J = 7.7 Hz, 1H), 7.56 (d, J = 7.7 Hz, 4H), 7.50 (t, J = 7.7 Hz, 1H), 7.36 (d, J = 8.4 Hz, 1H), 7.16 (dd, J = 7.9, 4.9 Hz, 4H), 2.35 (s, 3H).
^{13}C NMR (126 MHz, Chloroform-d) δ 142.47, 141.10, 139.72, 135.70, 132.16, 131.24, 130.71, 130.27, 129.94, 129.78, 128.62, 128.43, 128.30, 126.55, 126.47, 126.31, 126.13, 125.79, 125.43, 121.91, 121.52, 120.92, 20.49.
HRMS (ESI) calculated for $C_{27}H_{20}BrO_2S$ $[M+H]^+$ m/z 486.0289, found 488.0306.
3-(10-tosylphenanthren-9-yl)thiophene (3ja):

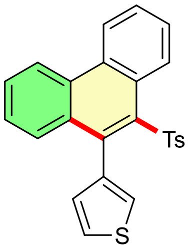

^{1}H NMR (400 MHz, Chloroform-d) δ 9.19 – 9.15 (m, 1H), 8.77 – 8.71 (m, 2H), 7.77 – 7.63 (m, 3H), 7.50 (d, J = 8.2 Hz, 3H), 7.45 (d, J = 8.3 Hz, 1H), 7.30 (dd, J = 4.8, 3.0 Hz, 1H), 7.18 (d, J = 2.4 Hz, 1H), 7.11 (d, J = 8.1 Hz, 2H), 6.89 (d, J = 4.8 Hz, 1H), 2.34 (s, 3H).
^{13}C NMR (101 MHz, Chloroform-d) δ 130.51, 129.65, 129.45, 129.07, 127.63, 127.49, 127.43, 127.22,

126.38, 125.79, 124.50, 123.01, 122.58, 77.48, 76.84, 21.68, 1.18.
HRMS (ESI) calculated for $C_{25}H_{19}O_2S_2$ $[M+H]^+$ m/z 414.0748, found 414.0752.

4-methyl-10-phenyl-9-tosylphenanthrene (3ka):

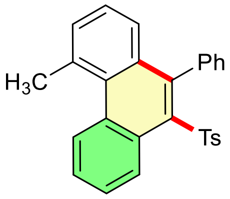

+

¹H NMR (500 MHz, Chloroform-d) δ 9.03 (dd, J = 6.5, 3.3 Hz, 1H), 8.62 (dd, J = 6.3, 3.4 Hz, 1H), 7.54 (dd, J = 6.5, 3.4 Hz, 3H), 7.41 (d, J = 8.2 Hz, 2H), 7.35 (d, J = 7.3 Hz, 1H), 7.29 (dd, J = 12.7, 5.5 Hz, 3H), 7.20 (s, 1H), 7.14 (d, J = 7.1 Hz, 2H), 7.01 (d, J = 8.1 Hz, 2H), 3.02 (s, 3H), 2.26 (s, 3H).
¹³C NMR (126 MHz, Chloroform-d) δ 143.14, 140.95, 137.74, 134.48, 133.71, 130.92, 129.40, 127.77, 127.71, 127.59, 127.39, 126.82, 126.79, 126.70, 126.11, 125.81, 77.41, 76.91, 26.40, 21.60.
HRMS (ESI) calculated for $C_{28}H_{23}O_2S$ $[M+H]^+$ m/z 422.1341, found 422.134 4.

3-methyl-10-phenyl-9-tosylphenanthrene (3la):

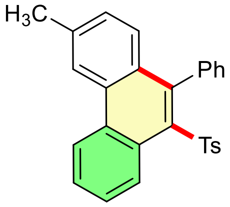

¹H NMR (400 MHz, Chloroform-d) δ 8.98 (d, J = 8.6 Hz, 1H), 8.74 (d, J = 8.2 Hz, 1H), 7.68 – 7.64 (m, 1H), 7.59 (d, J = 8.2 Hz, 3H), 7.46 – 7.43 (m, 3H), 7.29 (t, J = 7.3 Hz, 5H), 7.13 (d, J = 8.2 Hz, 2H), 2.61 (s, 3H), 2.32 (s, 3H).
¹³C NMR (101 MHz, Chloroform-d) δ 143.26, 140.00, 130.64, 130.04, 129.75, 129.71, 129.50, 129.06, 127.64, 127.58, 127.41, 127.25, 127.14, 126.51, 123.00, 122.34, 77.48, 76.84, 22.27, 21.66.
HRMS (ESI) calculated for $C_{28}H_{23}O_2S$ $[M+H]^+$ m/z 422.1341, found 422.1344.

2-methyl-10-phenyl-9-tosylphenanthrene (3ma)[1]:

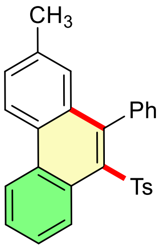

¹H NMR (400 MHz, Chloroform-d) δ 9.03 – 8.95 (m, 1H), 8.71 (d, J = 8.2 Hz, 1H), 8.62 (d, J = 8.5 Hz, 1H), 7.68 – 7.63 (m, 1H), 7.61 – 7.55 (m, 4H), 7.49 – 7.43 (m, 3H), 7.33 – 7.29 (m, 2H), 7.13 (d, J = 8.2 Hz, 3H), 2.38 (s, 3H), 2.33 (s, 3H).
¹³C NMR (101 MHz, Chloroform-d) δ 143.26, 140.92, 137.86, 137.25, 131.40, 130.21, 130.12, 129.50, 129.19, 127.65, 127.37, 127.15, 127.07, 126.55, 126.49, 122.80, 122.47, 77.48, 76.84, 21.85, 21.65.

2-methoxy-10-phenyl-9-tosylphenanthrene (3na):

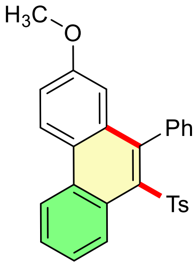

^{1}H NMR (500 MHz, Chloroform-d) δ 9.01 (d, J = 8.6 Hz, 1H), 8.63 (dd, J = 8.6, 3.1 Hz, 2H), 7.64 (t, J = 7.7 Hz, 1H), 7.58 (d, J = 8.3 Hz, 2H), 7.53 (t, J = 8.3 Hz, 1H), 7.45 – 7.42 (m, 3H), 7.37 – 7.35 (m, 1H), 7.29 (dd, J = 5.9, 2.5 Hz, 2H), 7.13 (d, J = 8.1 Hz, 2H), 6.71 (d, J = 2.6 Hz, 1H), 3.64 (s, 3H), 2.33 (s, 3H).
^{13}C NMR (126 MHz, Chloroform-d) δ 130.11, 129.51, 127.77, 127.72, 127.50, 127.27, 126.61, 126.51, 124.17, 122.52, 119.79, 110.40, 77.41, 76.91, 55.21, 21.62, 1.16.
HRMS (ESI) calculated for $C_{28}H_{23}O_3S$ $[M+H]^+$ m/z 438.1290, found 438.1302.
2-fluoro-10-phenyl-9-tosylphenanthrene (3oa):

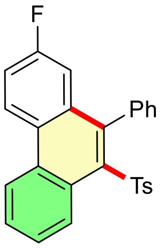

^{1}H NMR (500 MHz, Chloroform-d) δ 9.07 (d, J = 8.6 Hz, 1H), 8.72 (dd, J = 9.2, 5.5 Hz, 1H), 8.67 (d, J = 8.3 Hz, 1H), 7.70 (t, J = 7.6 Hz, 1H), 7.61 (s, 1H), 7.57 (d, J = 8.3 Hz, 2H), 7.48 (dd, J = 4.3, 1.9 Hz, 1H), 7.46 (t, J = 6.2 Hz, 3H), 7.27 (dd, J = 5.4, 1.7 Hz, 2H), 7.14 (d, J = 8.2 Hz, 2H), 7.01 (dd, J = 10.9, 2.7 Hz, 1H), 2.34 (s, 3H).
^{13}C NMR (126 MHz, Chloroform-d) δ 143.50, 140.85, 130.76, 130.15, 129.56, 127.92(t, J=0.32), 127.45, 127.34, 126.69, 125.00(d, J=6.06), 122.87, 118.60(d, J=19.19), 114.26(d, J=18.18), 77.41, 76.91, 21.63.
^{19}F NMR (471 MHz, Chloroform-d) δ -112.27 (ddd, J = 11.2, 7.5, 5.9 Hz).
HRMS (ESI) calculated for $C_{27}H_{20}FO_2S$ $[M+H]^+$ m/z 426.1090, found 426.1101.
2-chloro-10-phenyl-9-tosylphenanthrene (3pa)[1]:

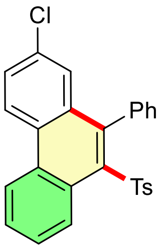

^{1}H NMR (500 MHz, Chloroform-d) δ 8.90 (d, J = 8.5 Hz, 1H), 8.49 (dd, J = 15.2, 8.6 Hz, 2H), 7.54 – 7.49 (m, 2H), 7.48 – 7.44 (m, 1H), 7.39 (d, J = 8.3 Hz, 2H), 7.32 – 7.26 (m, 3H), 7.16 (d, J = 2.2 Hz, 1H), 7.11 – 7.08 (m, 2H), 6.96 (d, J = 8.1 Hz, 2H), 2.17 (s, 3H).
^{13}C NMR (126 MHz, Chloroform-d) δ 143.50, 140.80, 137.02, 133.57, 133.12, 130.68, 130.57, 130.24, 130.02, 129.56, 128.68, 128.06, 127.90, 127.86, 127.77, 127.45, 127.04, 126.66, 124.24, 122.96, 77.41, 76.91, 21.61, 1.15.

2-bromo-10-phenyl-9-tosylphenanthrene (3qa):

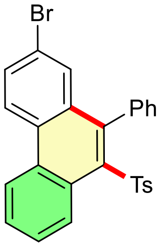

^{1}H NMR (500 MHz, Chloroform-d) δ 9.07 (d, J = 8.5 Hz, 1H), 8.68 (d, J = 8.2 Hz, 1H), 8.57 (d, J = 8.9 Hz, 1H), 7.81 (dd, J = 8.9, 1.9 Hz, 1H), 7.70 (d, J = 7.2 Hz, 1H), 7.65 – 7.62 (m, 1H), 7.55 (d, J = 8.3 Hz, 2H), 7.49 – 7.44 (m, 4H), 7.26 (s, 1H), 7.25 (s, 1H), 7.12 (d, J = 8.1 Hz, 2H), 2.33 (s, 3H).
^{13}C NMR (126 MHz, Chloroform-d) δ 143.50, 140.79, 136.95, 132.70, 131.85, 130.27, 129.56, 128.10, 127.90, 127.87, 127.48, 126.67, 124.33, 122.91, 121.78, 77.41, 76.91, 21.63, 1.16.
HRMS (ESI) calculated for $C_{27}H_{20}BrO_2S$ $[M+H]^+$ m/z 486.0289, found 486.0301.
R-methyl-4-phenyl-3-tosylquinolin-2(1H)-one (3ra):

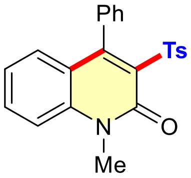

^{1}H NMR (400 MHz, Chloroform-d) δ 7.93 (d, J = 8.3 Hz, 2H), 7.46 – 7.33 (m, 5H), 7.17 – 7.12 (m, 2H), 6.43 (s, 4H), 2.83 (s, 3H), 2.44 (s, 3H).
^{13}C NMR (101 MHz, Chloroform-d) δ 183.84, 144.19, 133.44, 130.26, 128.92, 128.84, 127.84, 77.48, 76.84, 70.51, 27.15, 1.15.
HRMS (ESI) calculated for $C_{23}H_{20}NO_3S$ $[M+H]^+$ m/z 389.1086, found 389.1094.
3-((4-ethylphenyl)sulfonyl)-4-phenyl-2H-chromen-2-one (3sq):

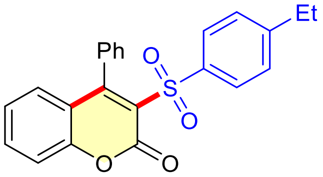

^{1}H NMR (400 MHz, Chloroform-d) δ 7.92 (d, J = 8.3 Hz, 2H), 7.62 – 7.56 (m, 4H), 7.34 (dd, J = 7.1, 2.9 Hz, 4H), 7.31 (s, 1H), 7.18 (t, J = 7.6 Hz, 1H), 7.03 – 6.99 (m, 1H), 2.71 (q, J = 7.6 Hz, 2H), 1.24 (t, J = 7.6 Hz, 3H).
^{13}C NMR (101 MHz, Chloroform-d) δ 150.97, 137.54, 134.68, 132.82, 130.06, 129.54, 129.35, 128.28, 127.61, 124.91, 120.43, 116.91, 77.48, 76.84, 29.07, 15.18.
HRMS (ESI) calculated for $C_{23}H_{19}O_4S$ $[M+H]^+$ m/z 390.0926, found 390.0933.
R-2,4-dimethyl-4-(tosylmethyl)isoquinoline-1,3(2H,4H)-dione (3ta)[3]:

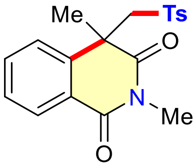

^{1}H NMR (400 MHz, Chloroform-d) δ 8.27 (d, J = 6.3 Hz, 1H), 7.42 – 7.39 (m, 2H), 7.36 (d, J = 8.2 Hz, 2H),

7.21 – 7.18 (m, 1H), 7.15 (d, J = 7.8 Hz, 2H), 4.46 – 4.41 (m, 1H), 3.87 (d, J = 14.5 Hz, 1H), 3.38 (s, 3H), 2.38 (s, 3H), 1.57 (s, 3H).

^{13}C NMR (101 MHz, Chloroform-d) δ 144.65, 139.31, 133.54, 129.80, 129.40, 128.17, 127.80, 126.01, 77.48, 76.84, 64.94, 45.52, 31.78, 27.66, 21.69, 1.15.

(R)-5-methyl-5-(tosylmethyl)benzo[4,5]imidazo[2,1-a]isoquinolin-6(5H)-one (3ua)[4]:

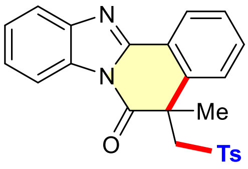

^{1}H NMR (400 MHz, Chloroform-d) δ 8.50 (d, J = 7.8 Hz, 1H), 8.24 (dd, J = 6.5, 2.4 Hz, 1H), 7.82 (dd, J = 6.6, 2.3 Hz, 1H), 7.50 – 7.40 (m, 4H), 7.29 (d, J = 8.4 Hz, 3H), 6.98 (d, J = 8.1 Hz, 2H), 4.55 (d, J = 14.7 Hz, 1H), 3.99 (d, J = 14.7 Hz, 1H), 2.14 (s, 3H), 1.68 (s, 3H).

^{13}C NMR (101 MHz, Chloroform-d) δ 170.60, 137.31, 131.46, 130.41, 129.62, 128.58, 128.16, 128.06, 127.13, 126.31, 126.15, 125.83, 120.01, 115.90, 77.48, 76.84, 64.61, 31.72, 21.46, 1.17.

5. Reference

(1) Tian, H.-D.; Fu, Z.-H.; Li, C.; Lin, H.-C.; Li, M.; Ni, S.-F.; Wen, L.-R.; Zhang, L.-B. Selective Electrochemical Synthesis of 9-Aryl-10-sulfonyl Substituted Phenanthrene from Alkynes and Sulfonyl Hydrazides. Organic Letters **2022**, 24 (50), 9322-9326.

(2) Ma, T.; Bian, M.; Lin, X.; Yang, Z.; Yang, X.; Duan, J.; Zhu, N.; Liu, C.; Fang, Z.; Guo, K. Visible-Light-Promoted Intramolecular Annulation of 2-Alkynylbiphenyl Compounds to Synthesize 9-Sulfenylphenanthrenes Under Metal-Free and Additive-Free Conditions. ChemPhotoChem **2022**, 6 (11).

(3) Xia, X.-F.; Zhu, S.-L.; Wang, D.; Liang, Y.-M. Sulfide and Sulfonyl Chloride as Sulfonylating Precursors for the Synthesis of Sulfone-Containing Isoquinolinonediones. Advanced Synthesis & Catalysis **2017**, 359 (5), 859-865.

(4) Tang, Y.; Duan, J.; Yang, B.; He, Y.; Du, C.; Zhang, X. Visible-light-promoted organic-dye-catalyzed sulfonylation/cyclization to access indolo[2,1-a]isoquinoline derivatives. Organic & Biomolecular Chemistry **2023**, 21 (40), 8152-8161.

(5) Gaussian 16 Rev. C.01; Wallingford, CT, 2016.

(6) (a)Weigend, F.; Ahlrichs, R. Phys. Chem. Chem. Phys. 2005, 7, 3297. (b) Zhao, Y.; Truhlar, D. Theor. Chem. Acc. 2008, 120, 215. (c) Qi, X.; Zhang, H.; Shao, A.; Zhu, L.; Xu, T.; Gao, M.; Liu, C.; Lan, Y. ACS Catal. 2015, 5, 6640. (d) Zhao, Y.; Truhlar, D. G. J. Chem. Phys. 2006, 125, 194101.

(7) Marenich, A. V.; Cramer, C. J.; Truhlar, D. G. Generalized born solvation model SM12. J. Chem. Theory Comput. 2013, 9, 609-620.

(8) Legault, C. Y. CYLview 1.0b, Université de Sherbrooke, Canada, 2009, http://www.cylview.org.

6.^{1}H NMR,^{13}C NMR and ^{19}F NMR Spectra of Products

3ab ^{1}H NMR (400MHz) and ^{13}C NMR (101MHz)

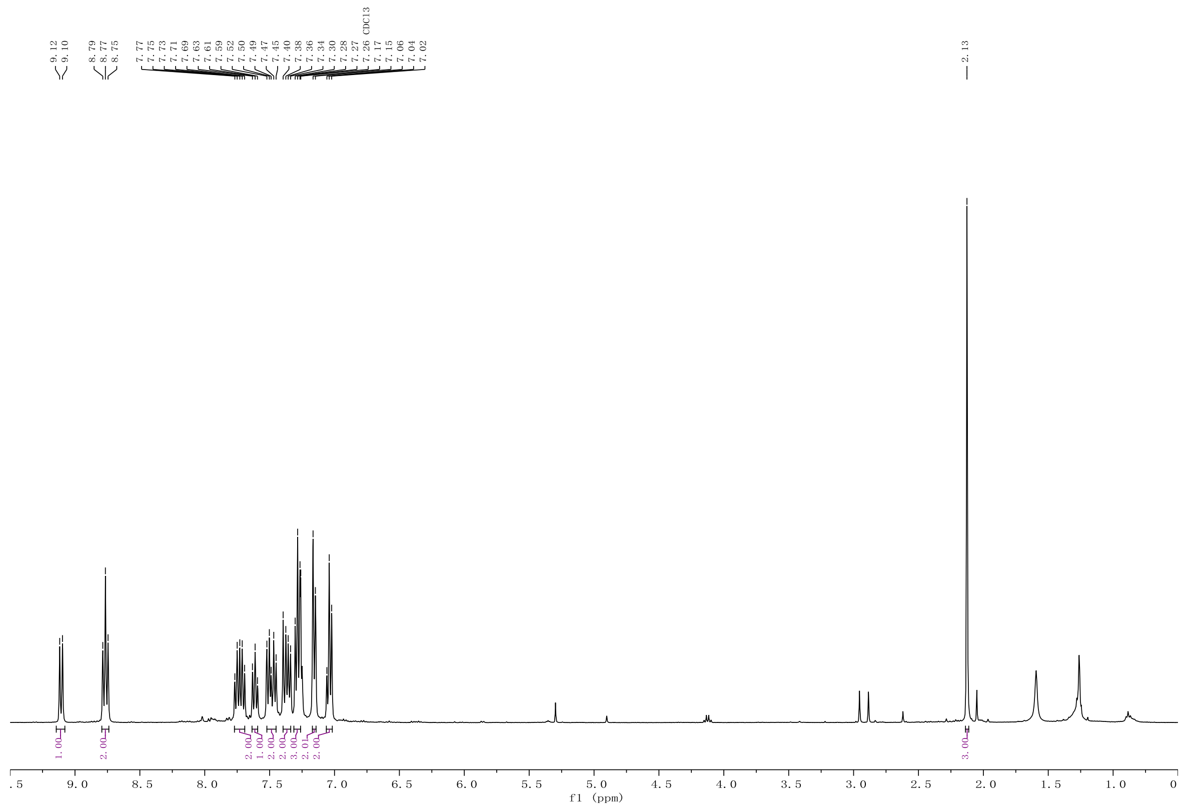

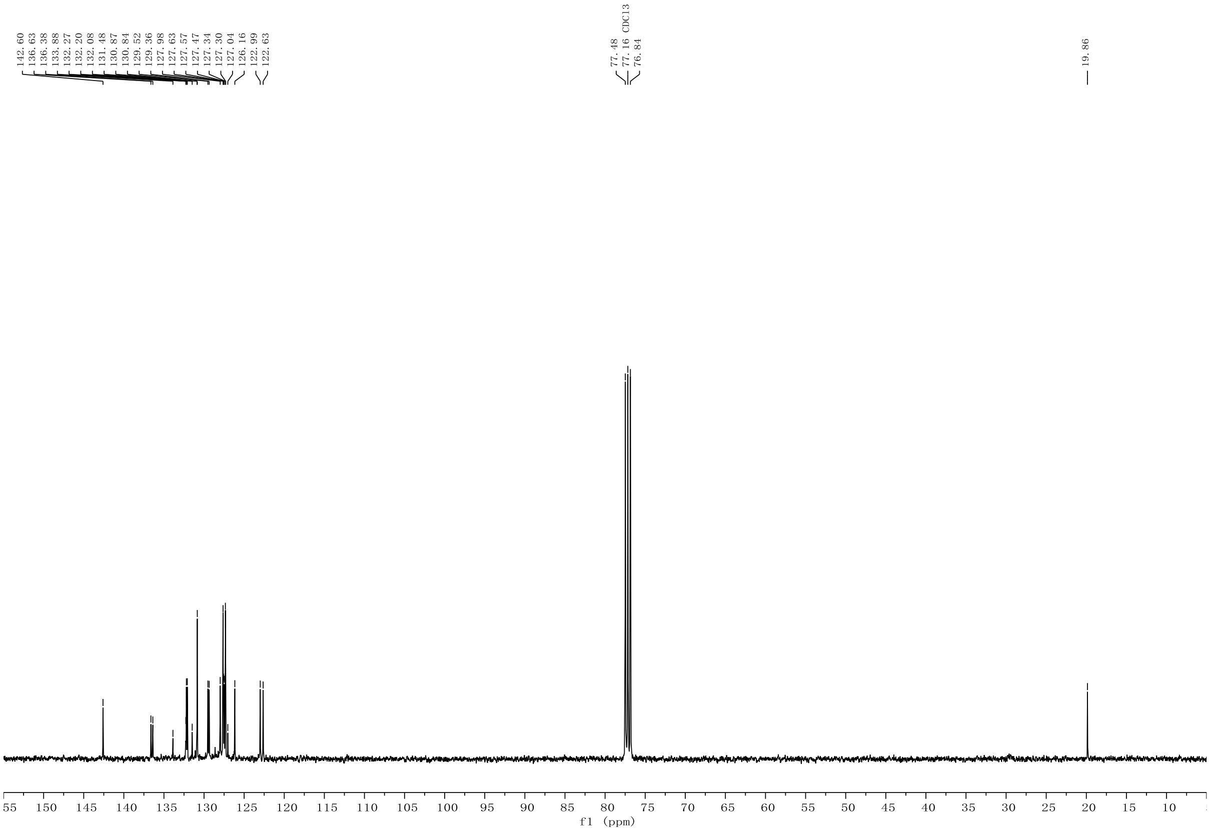

3ac ^{1}H NMR (400MHz) and ^{13}C NMR (101MHz)

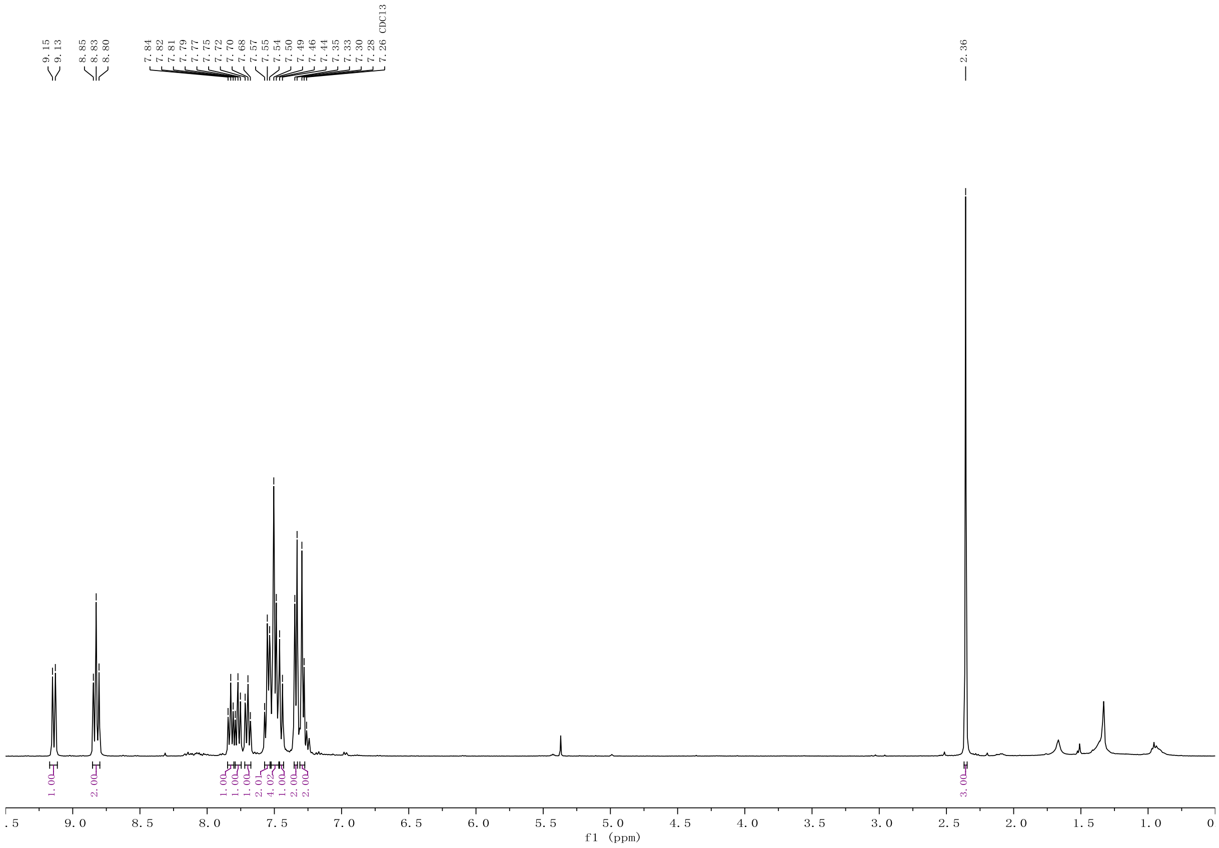

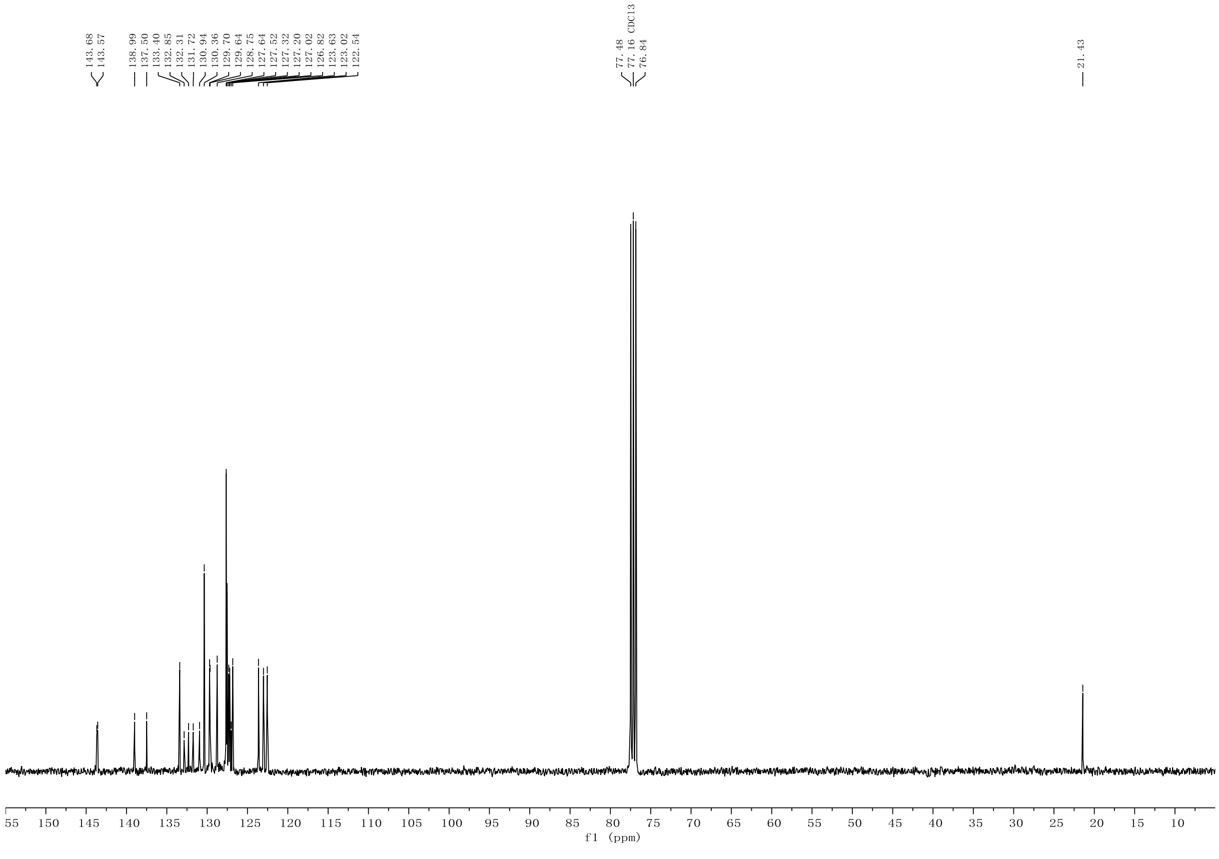

3ad ^{1}H NMR (400MHz) and ^{13}C NMR (101MHz)

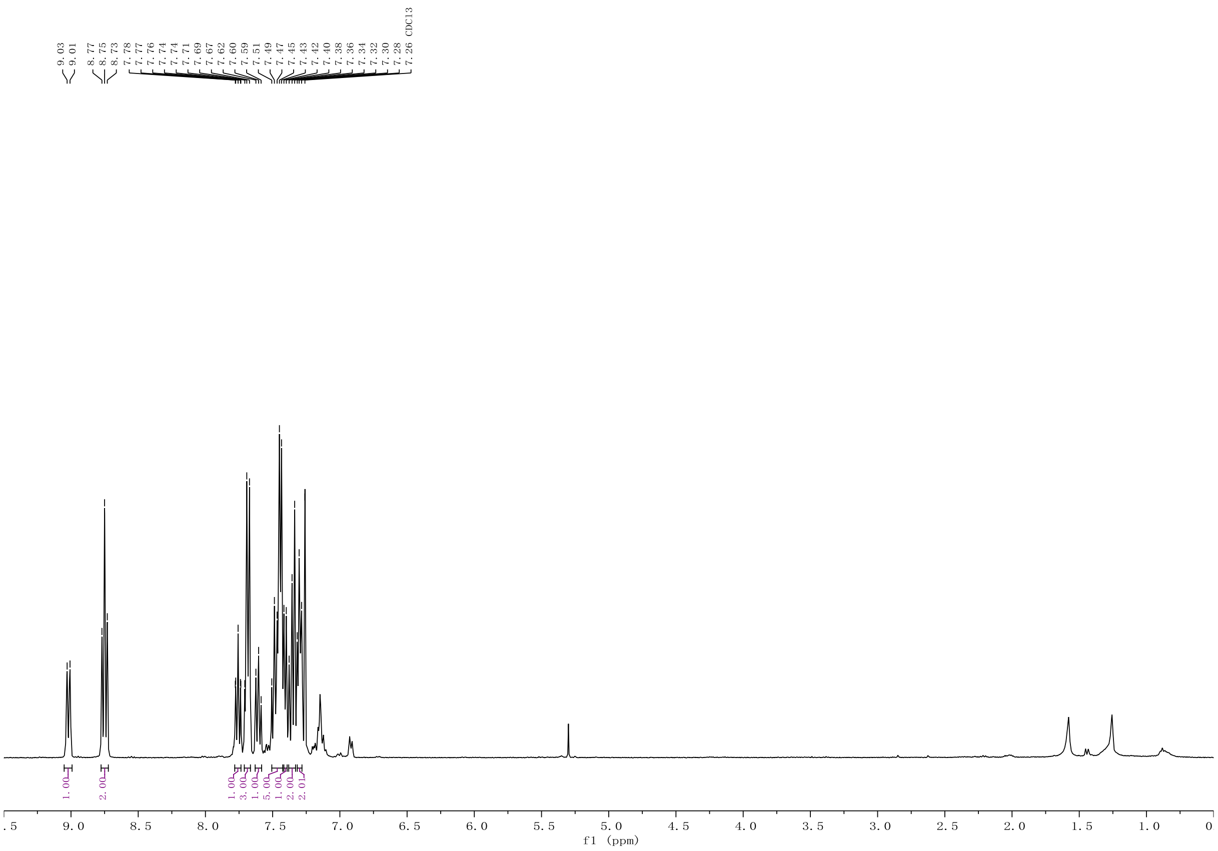

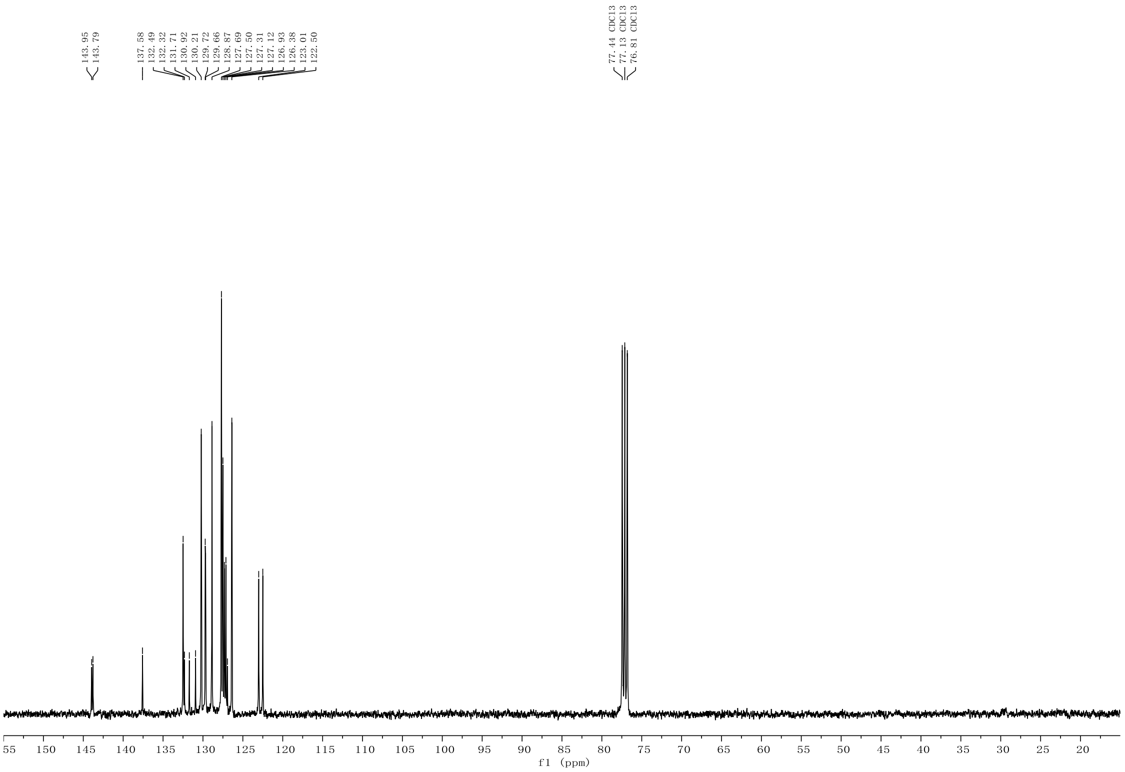

3ae ^{1}H NMR (400MHz) and ^{13}C NMR (101MHz)

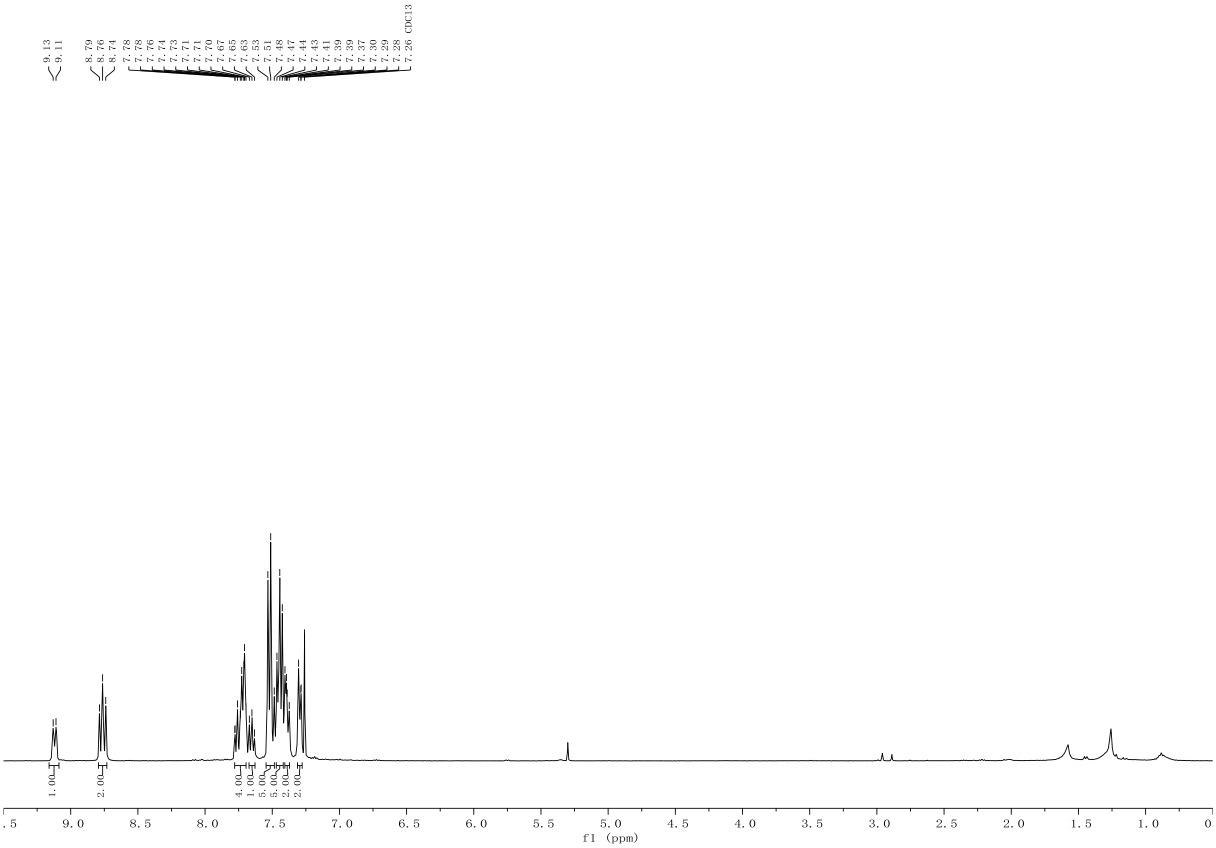

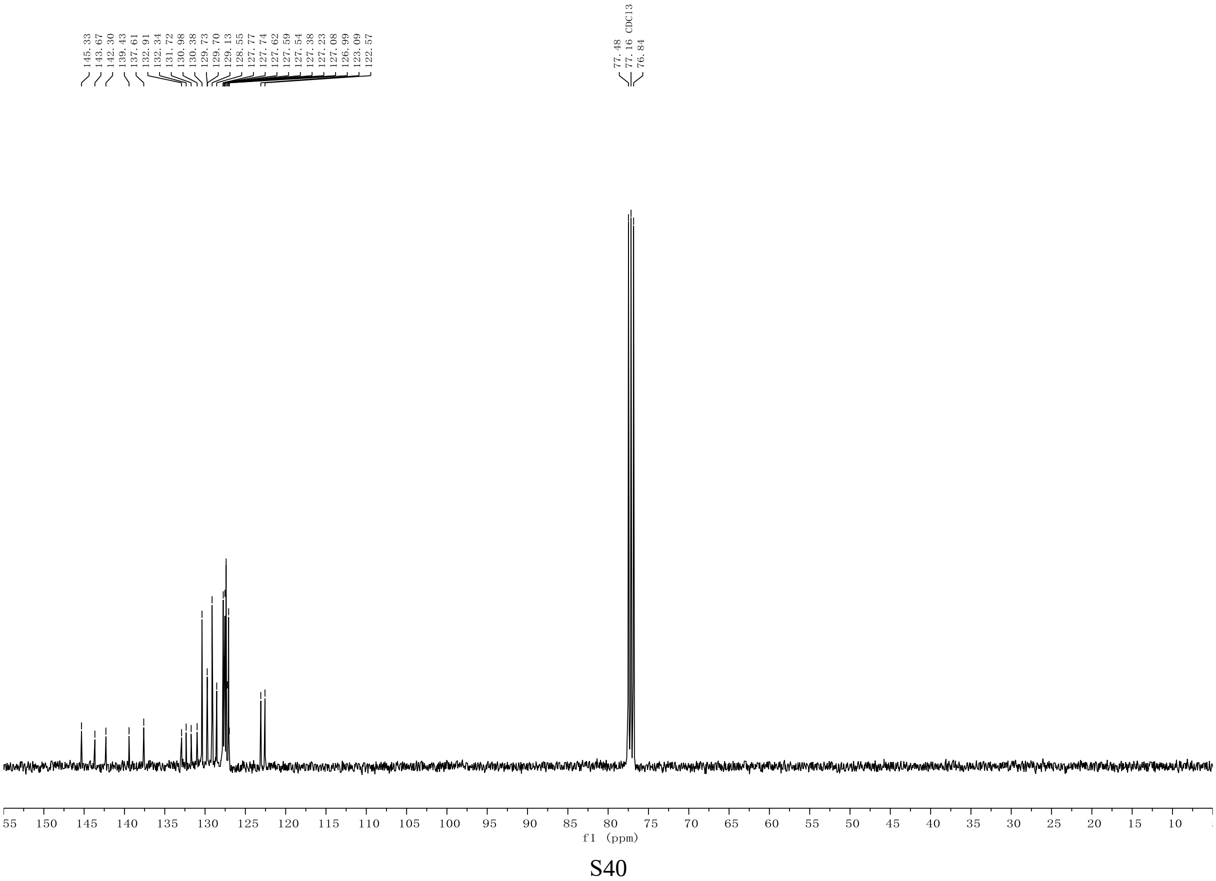

3aa ^{1}H NMR (500MHz) and ^{13}C NMR (101MHz)

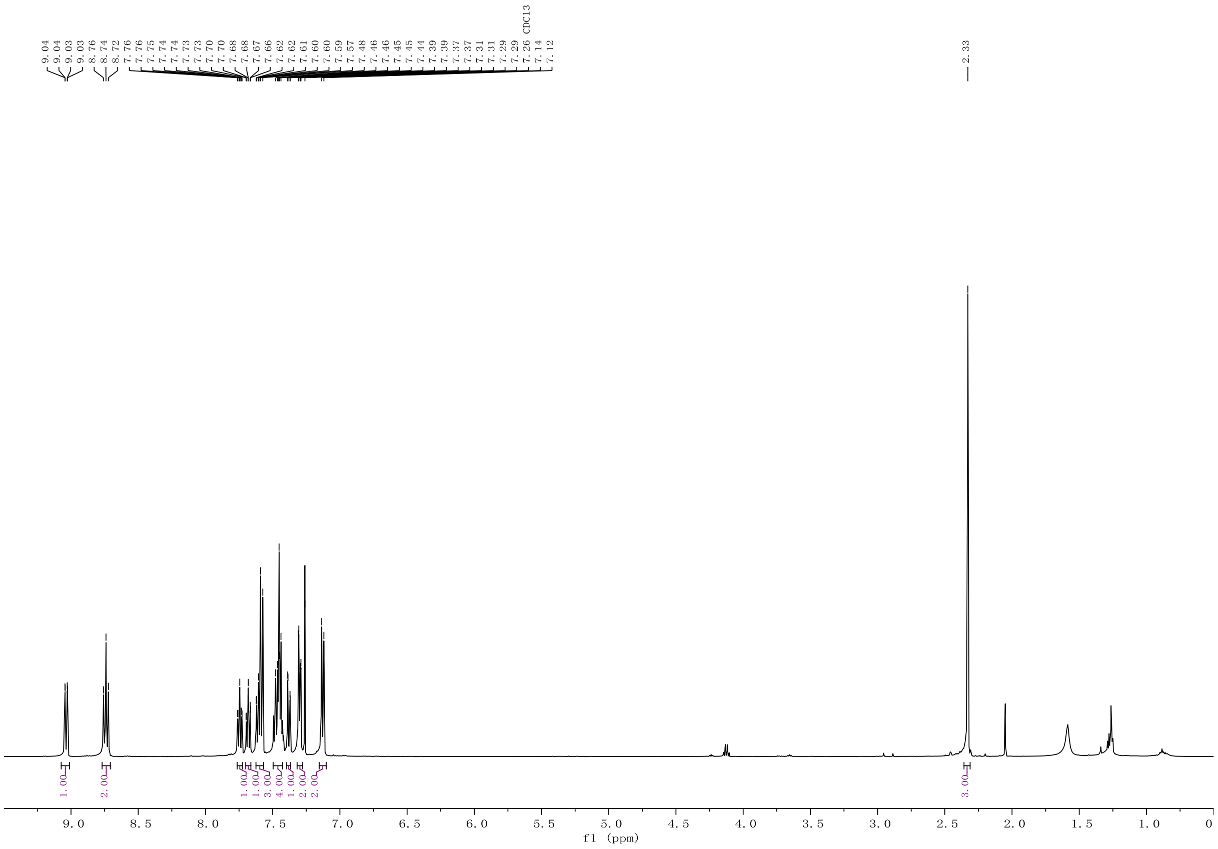

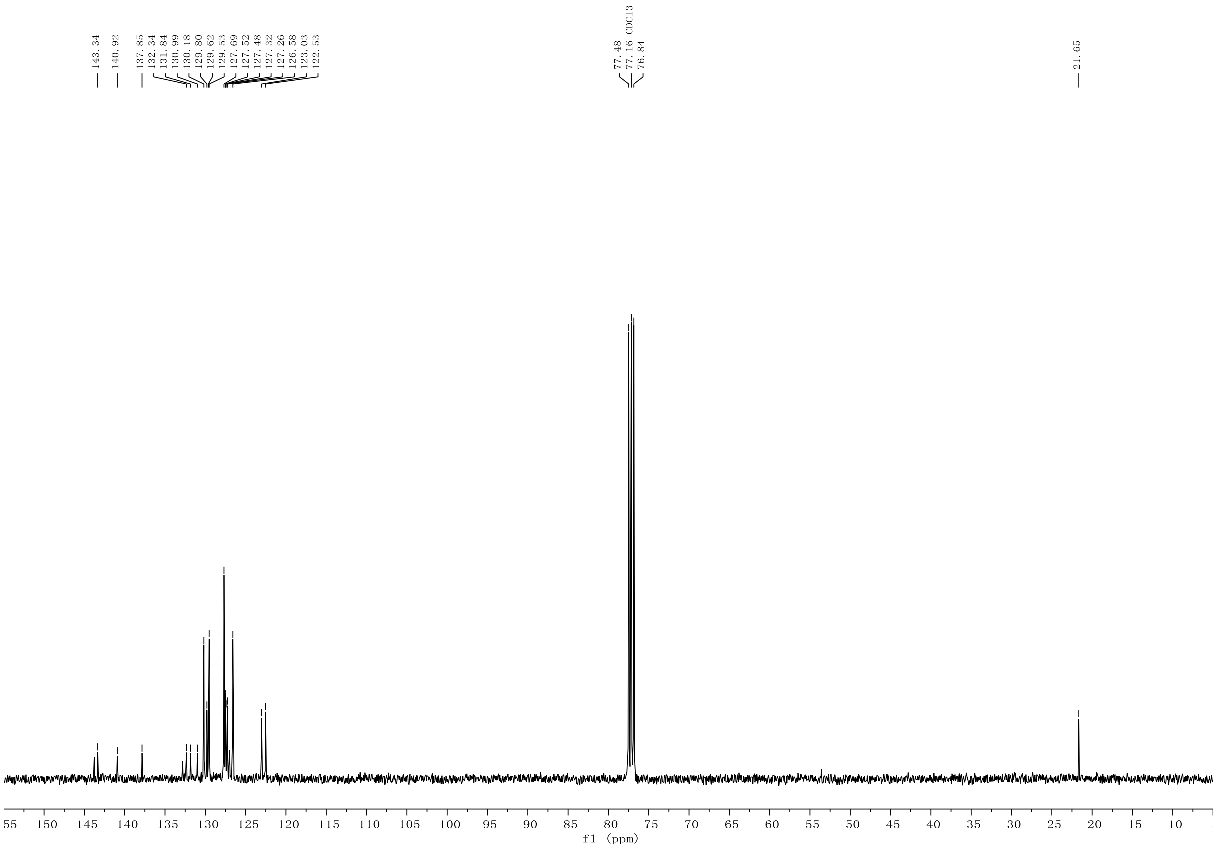

3af ^{1}H NMR (400MHz) and ^{13}C NMR (101MHz)

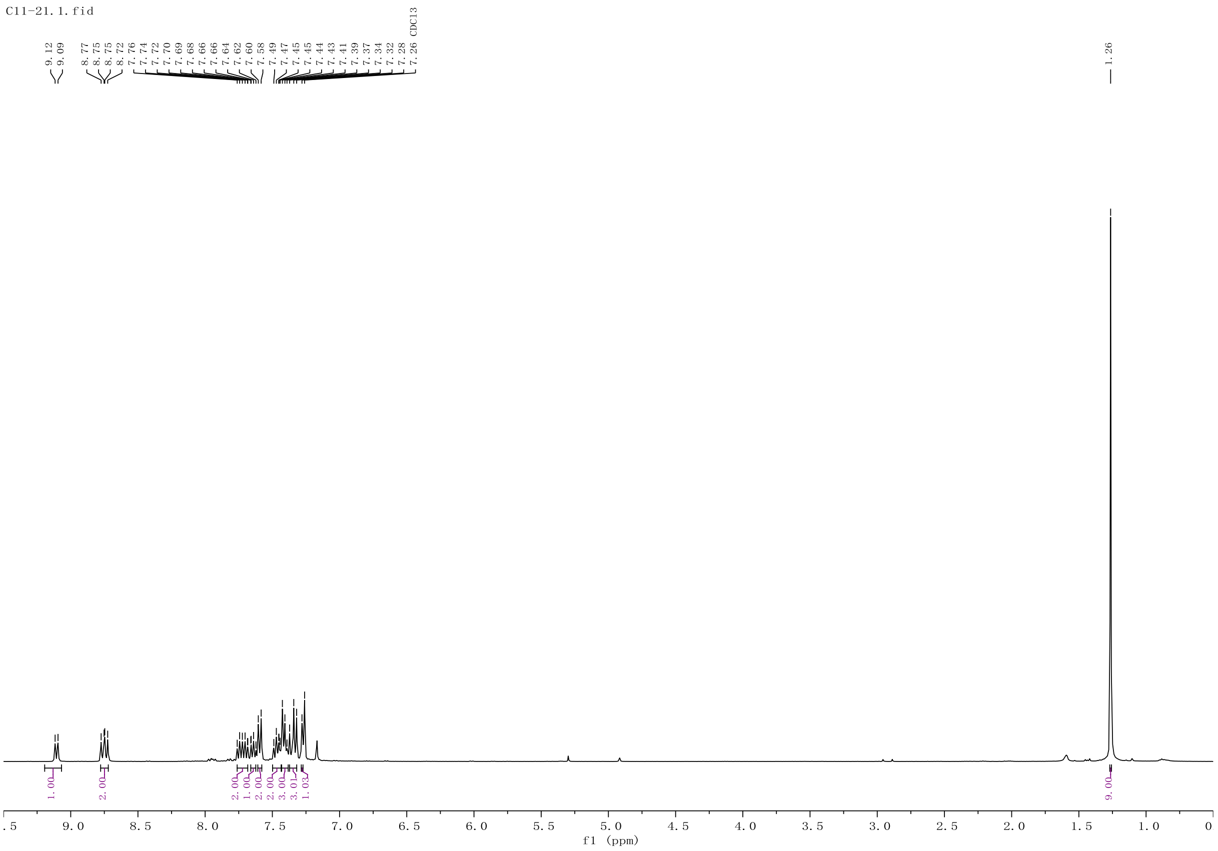

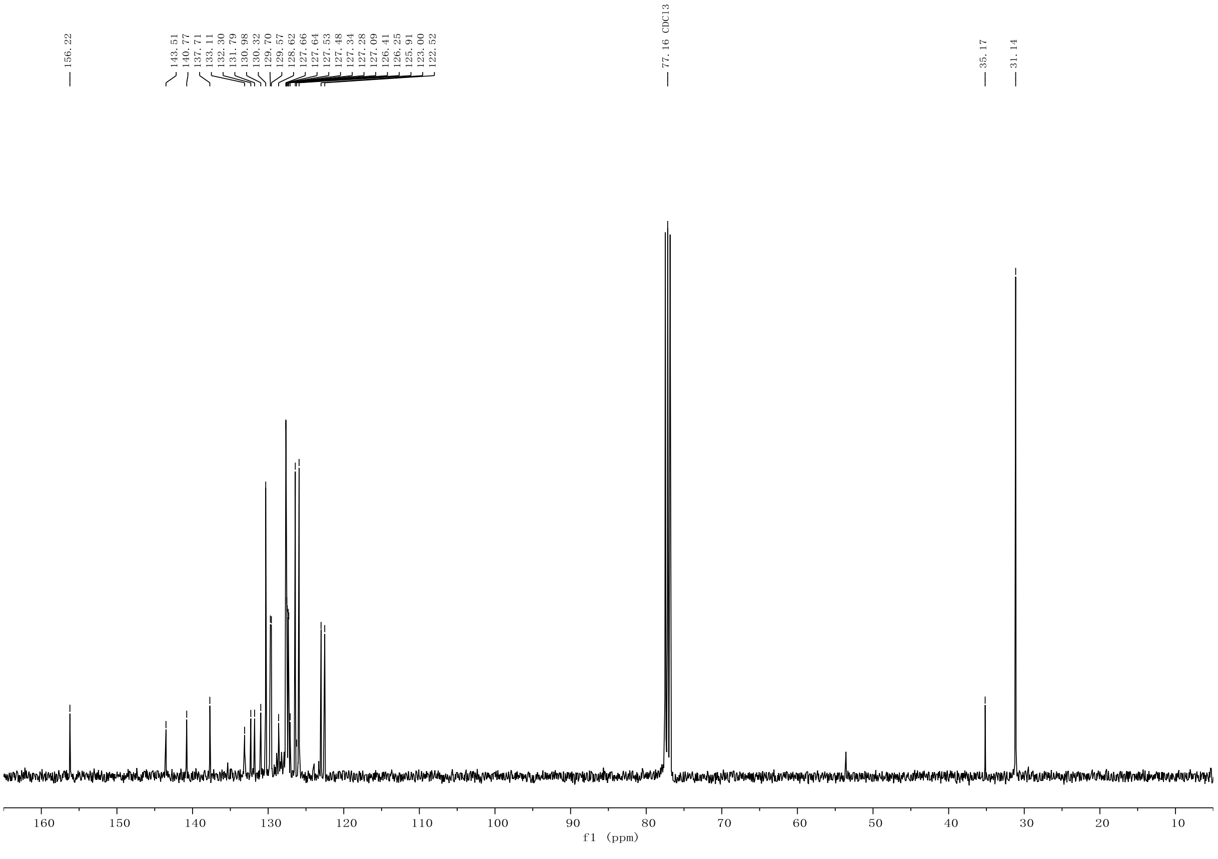

3ag ^{1}H NMR (400MHz) and ^{13}C NMR (101MHz)

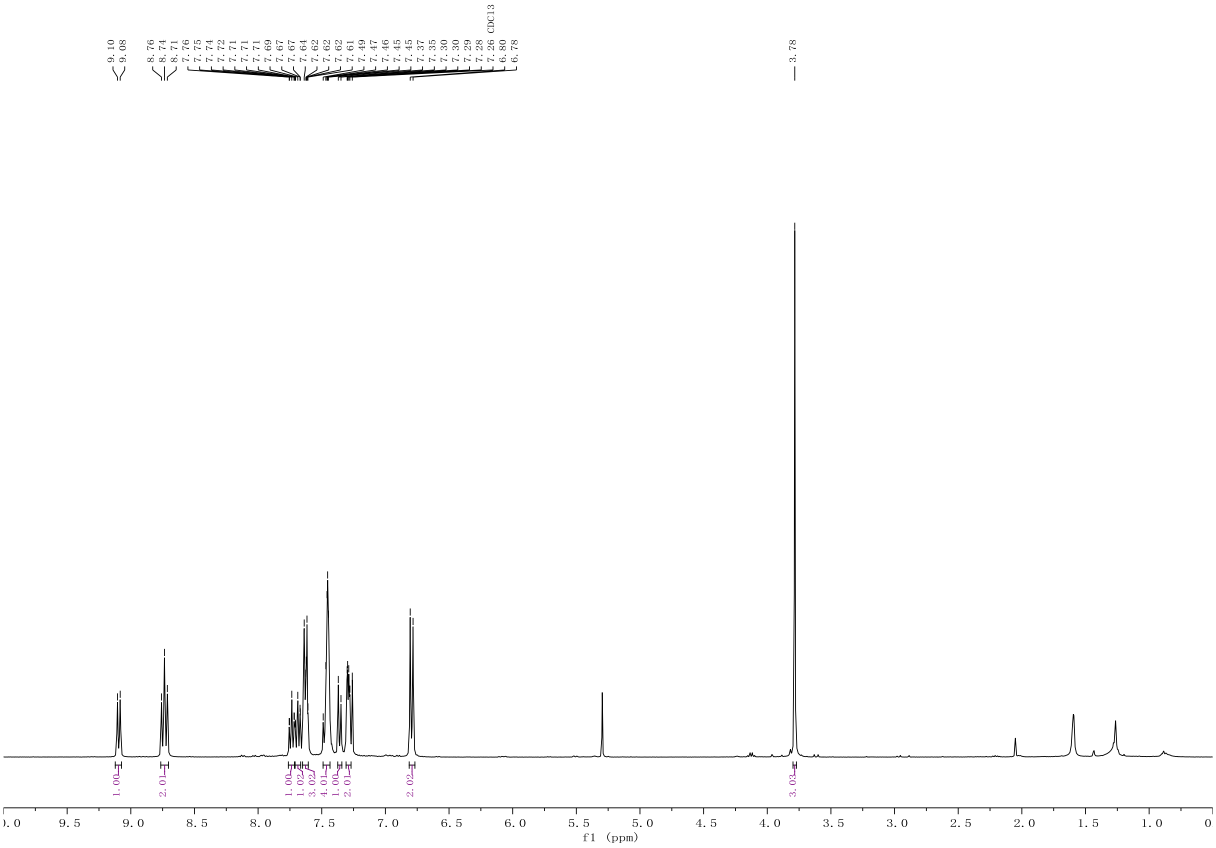

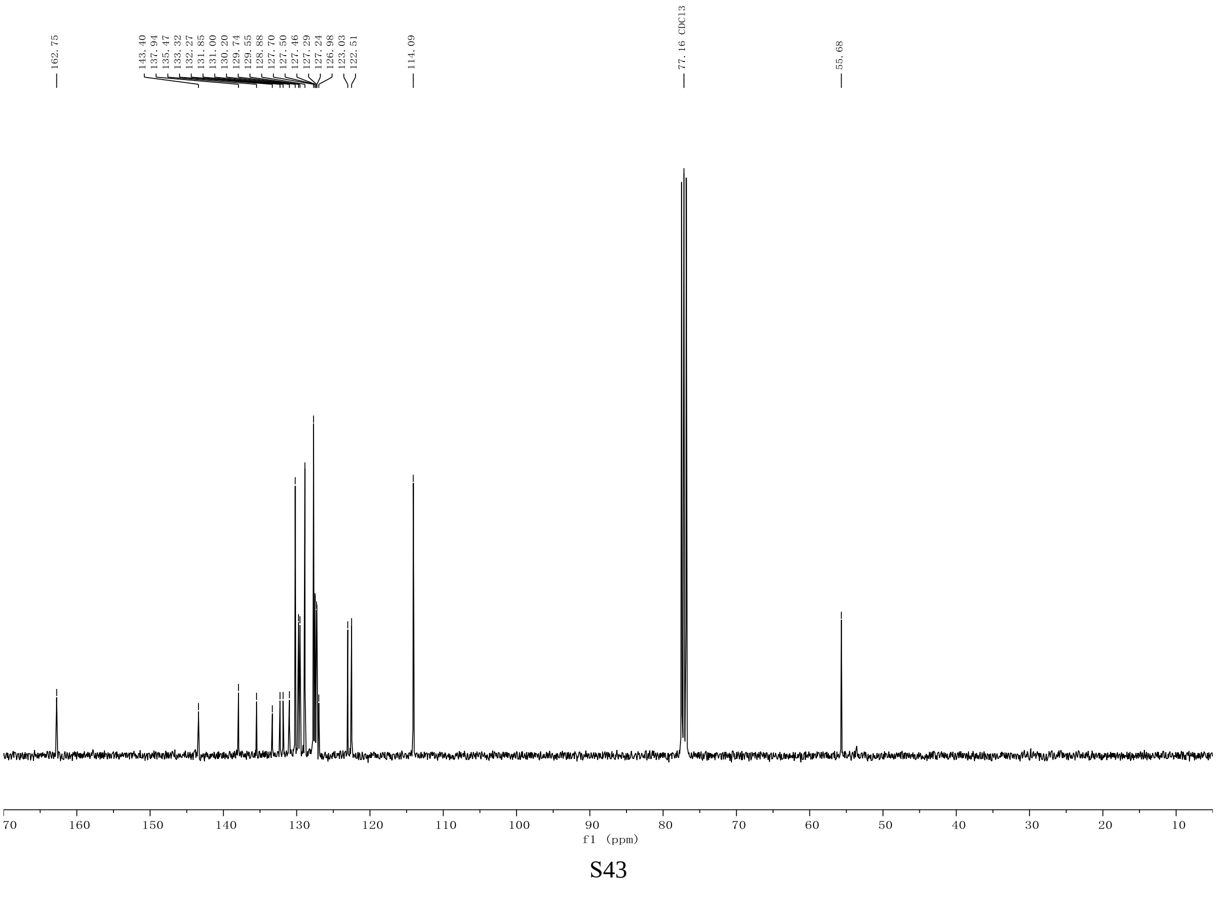

3ah ^{1}H NMR (400MHz), ^{13}C NMR (101MHz) and ^{19}F NMR (376 MHz)

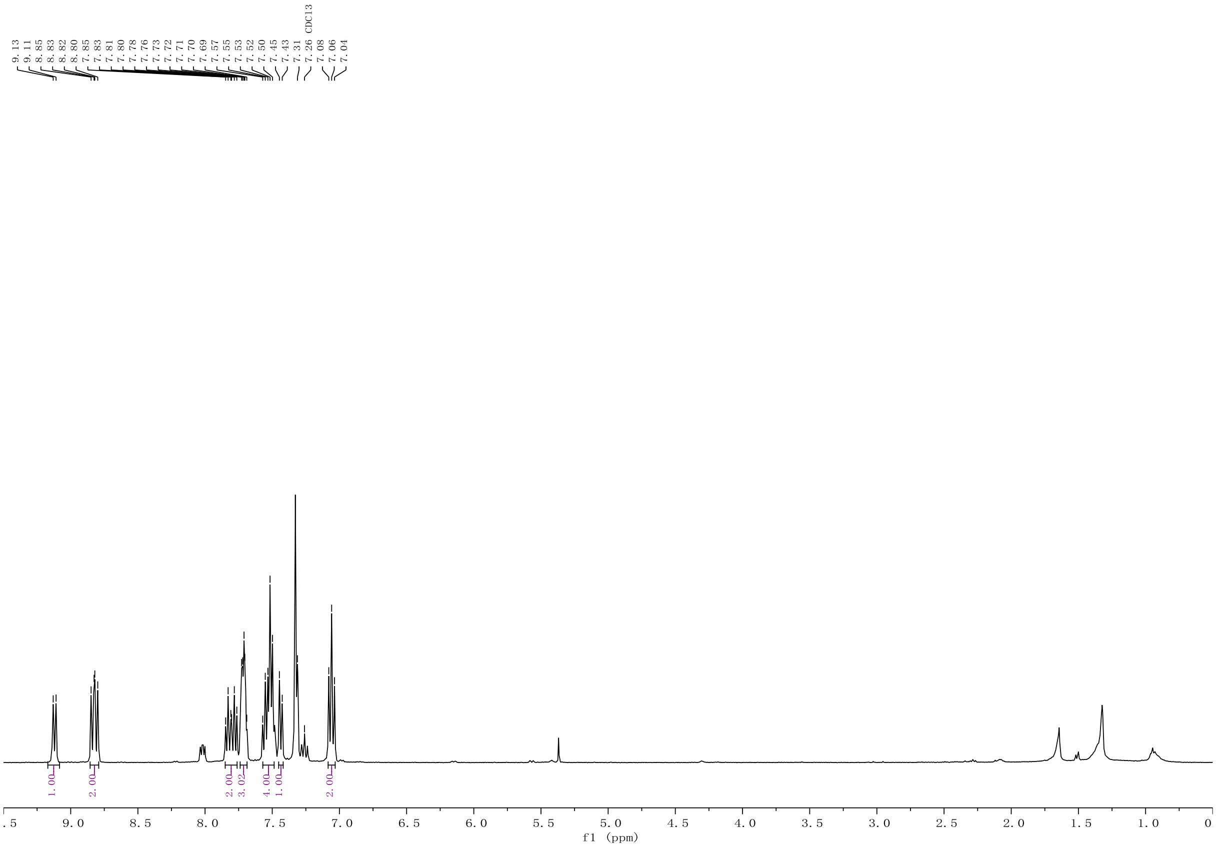

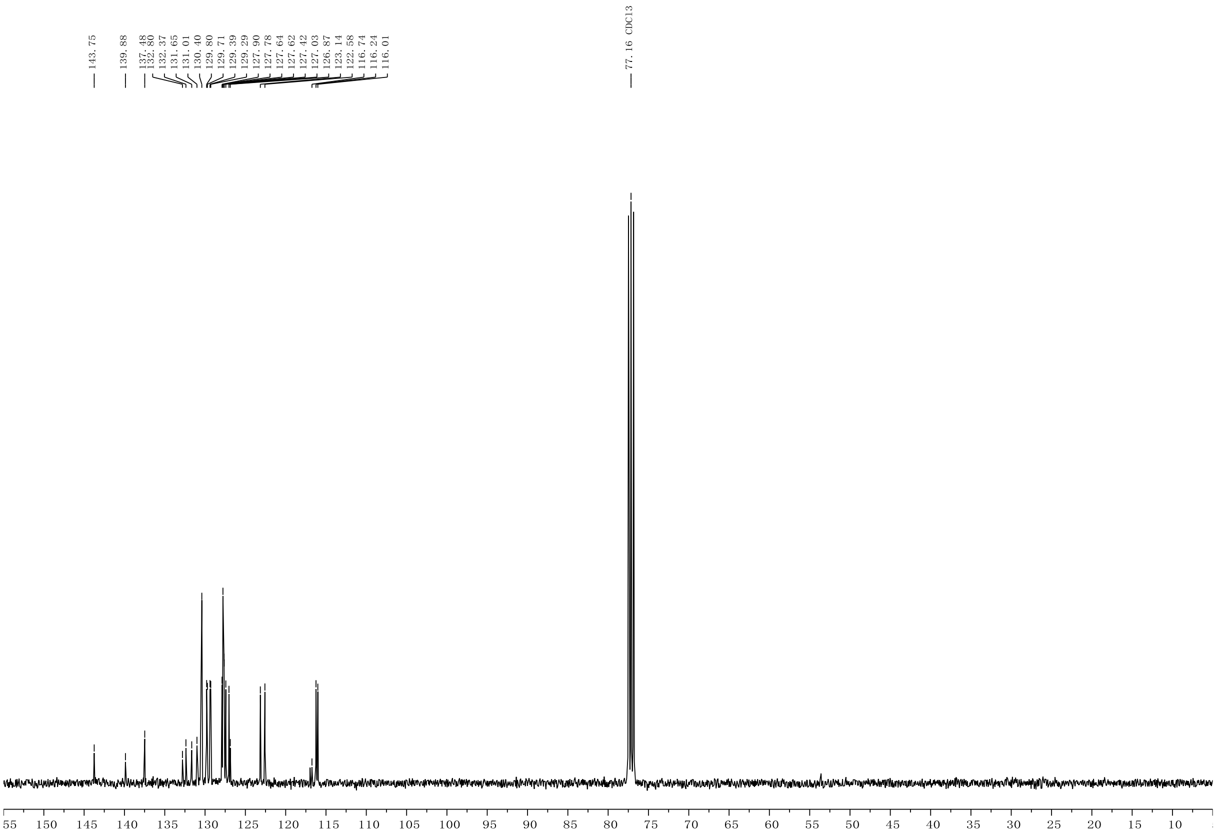

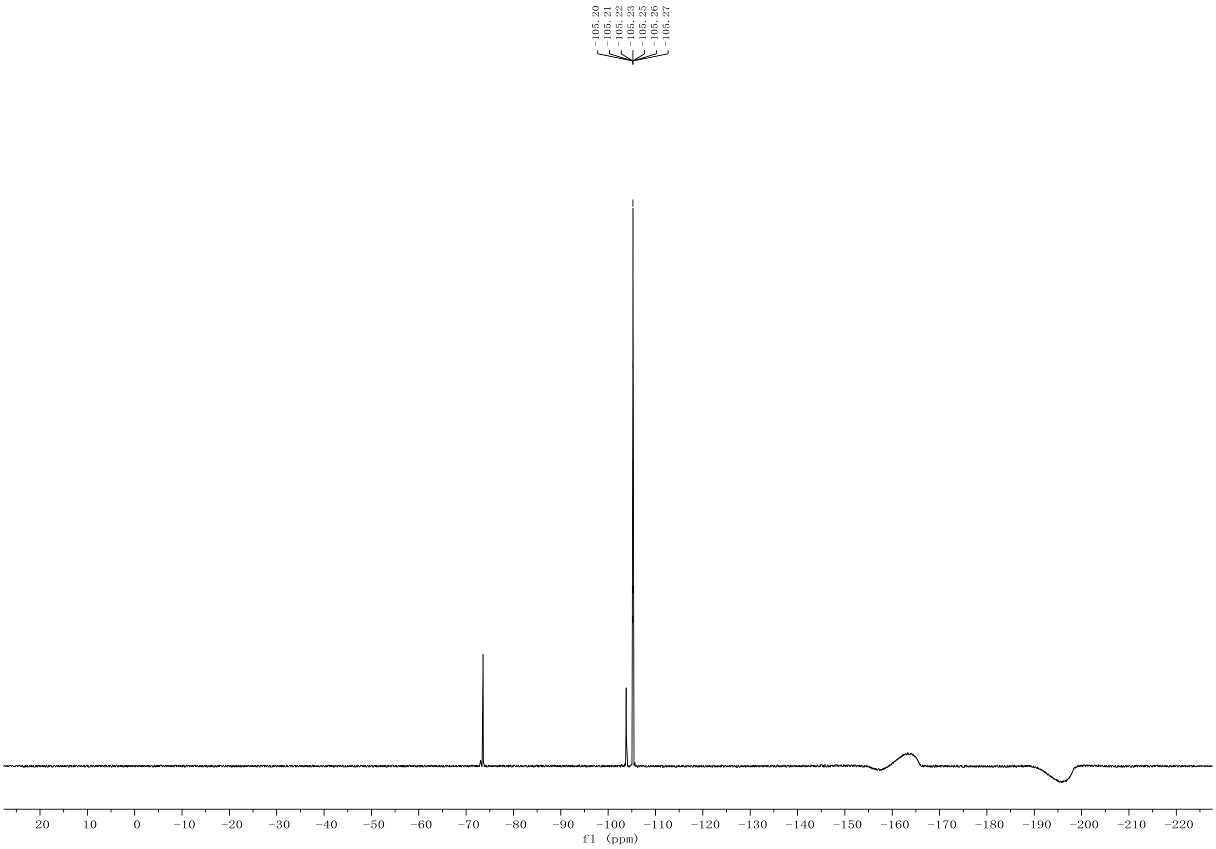
-105.20
-105.21
-105.22
-105.23
-105.25
-105.26
-105.27
20 10 0 -10 -20 -30 -40 -50 -60 -70 -80 -90 -100 -110 -120 -130 -140 -150 -160 -170 -180 -190 -200 -210 -220
f1 (ppm)

3ai ^{1}H NMR (400MHz) and ^{13}C NMR (101MHz)

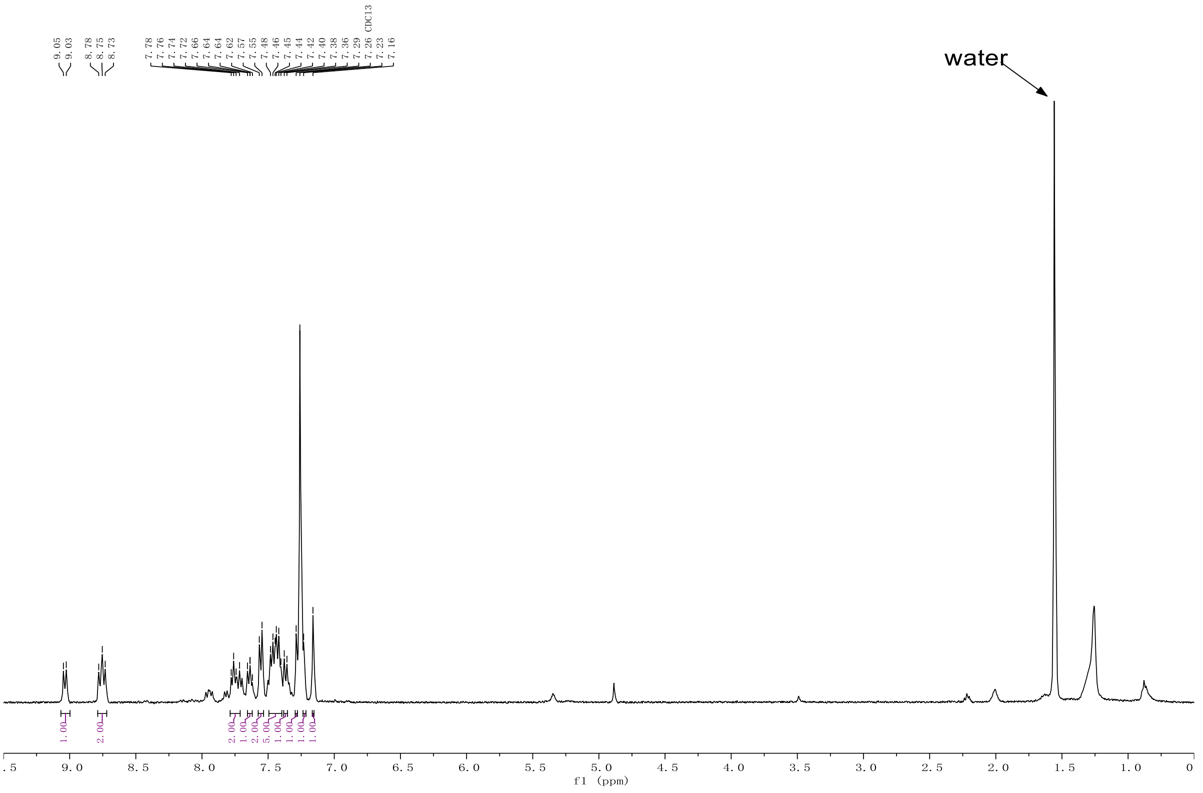

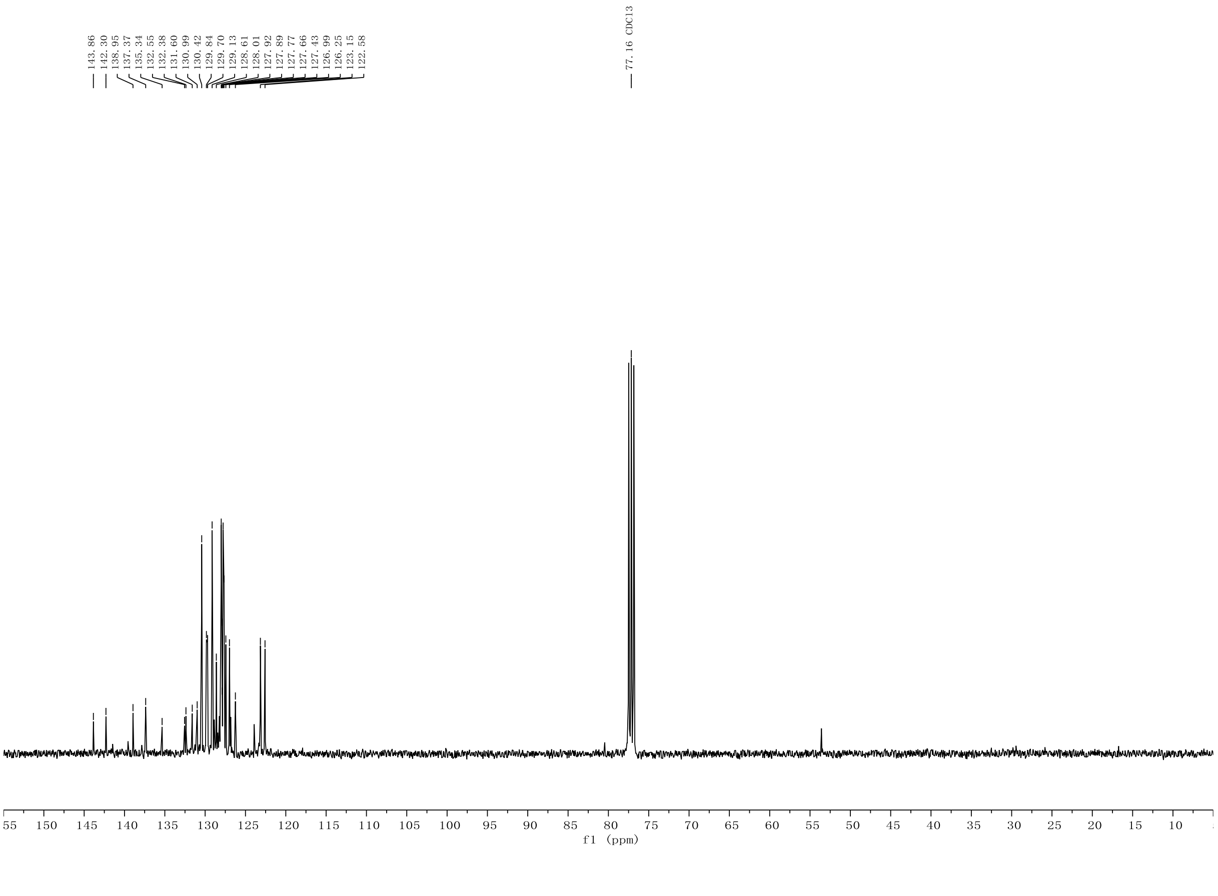

3aj ^{1}H NMR (400MHz) and ^{13}C NMR (101MHz)

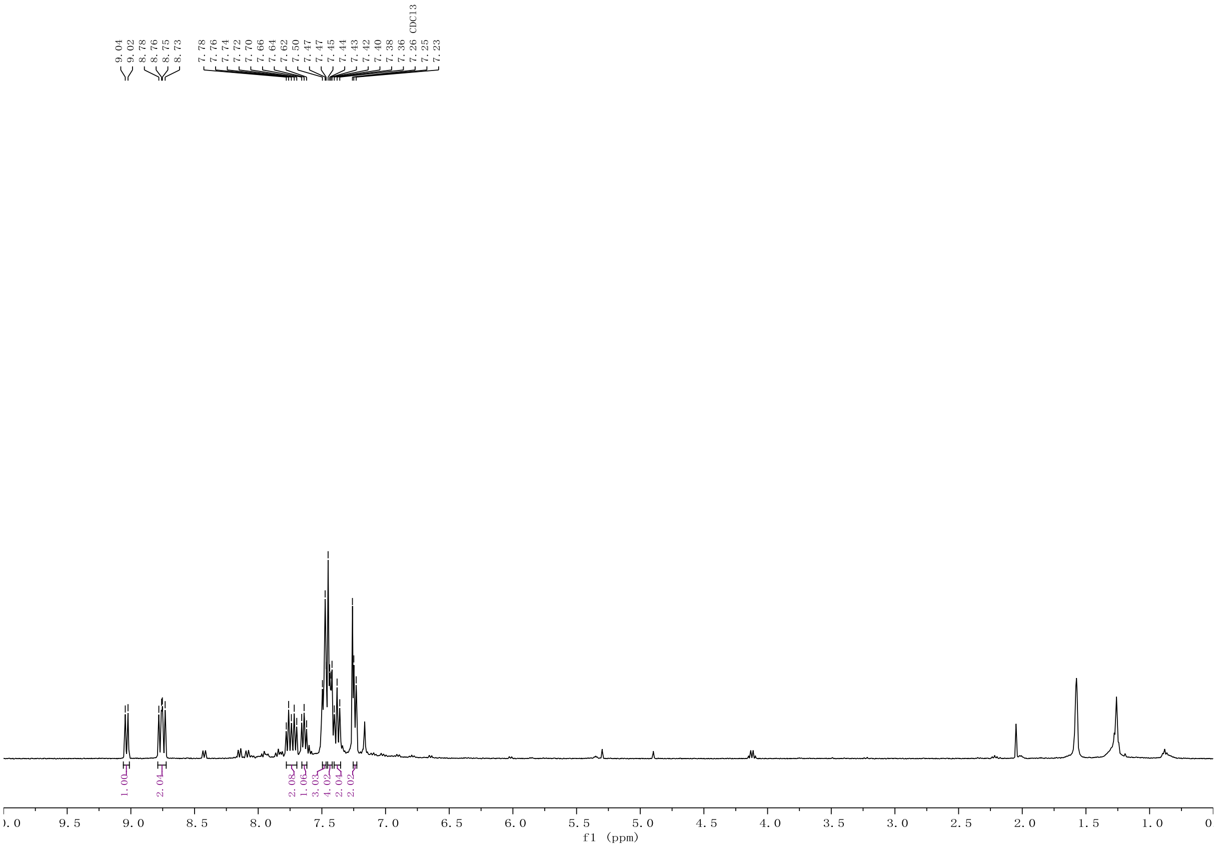

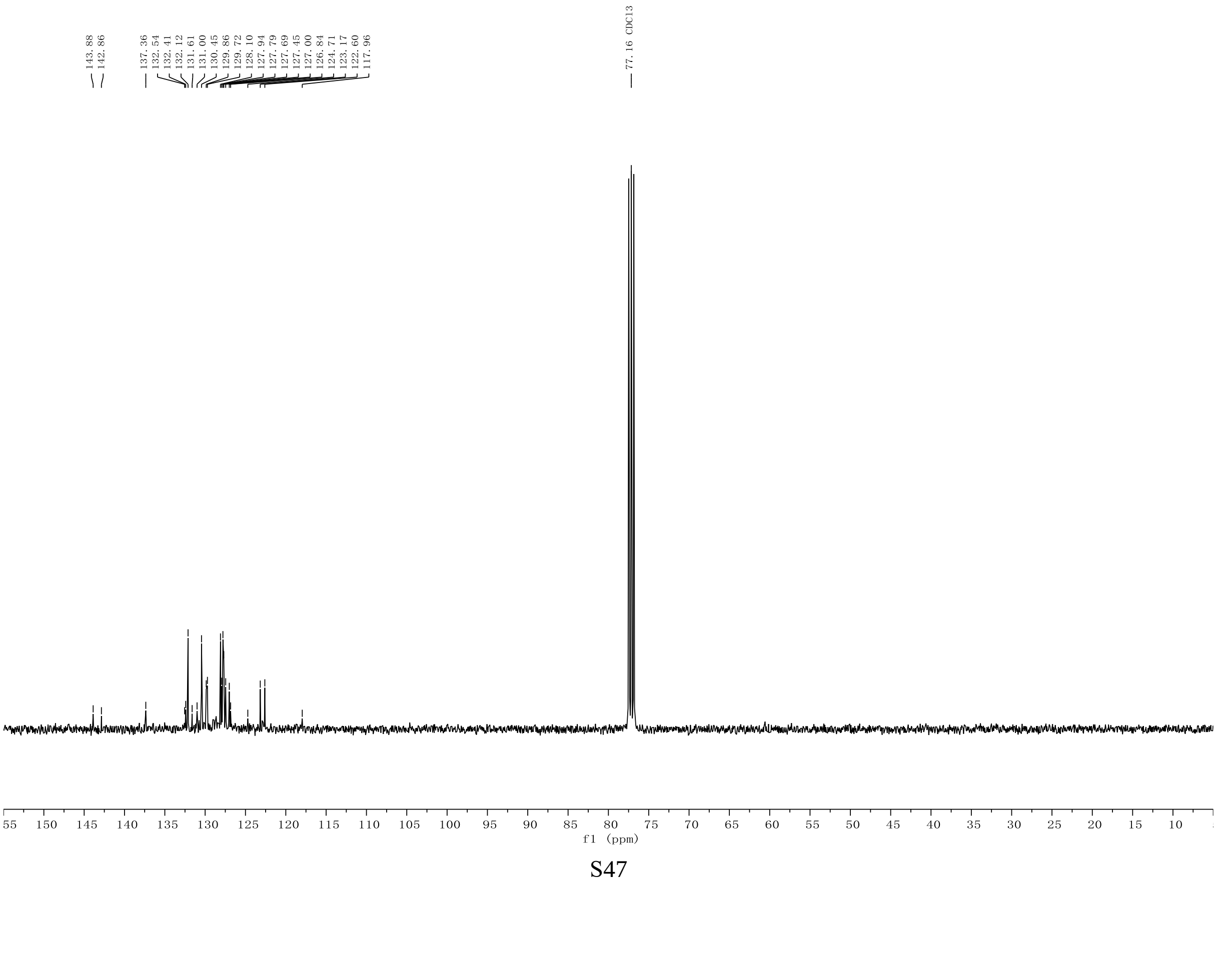

3ak ^{1}H NMR (400MHz) and ^{13}C NMR (101MHz)

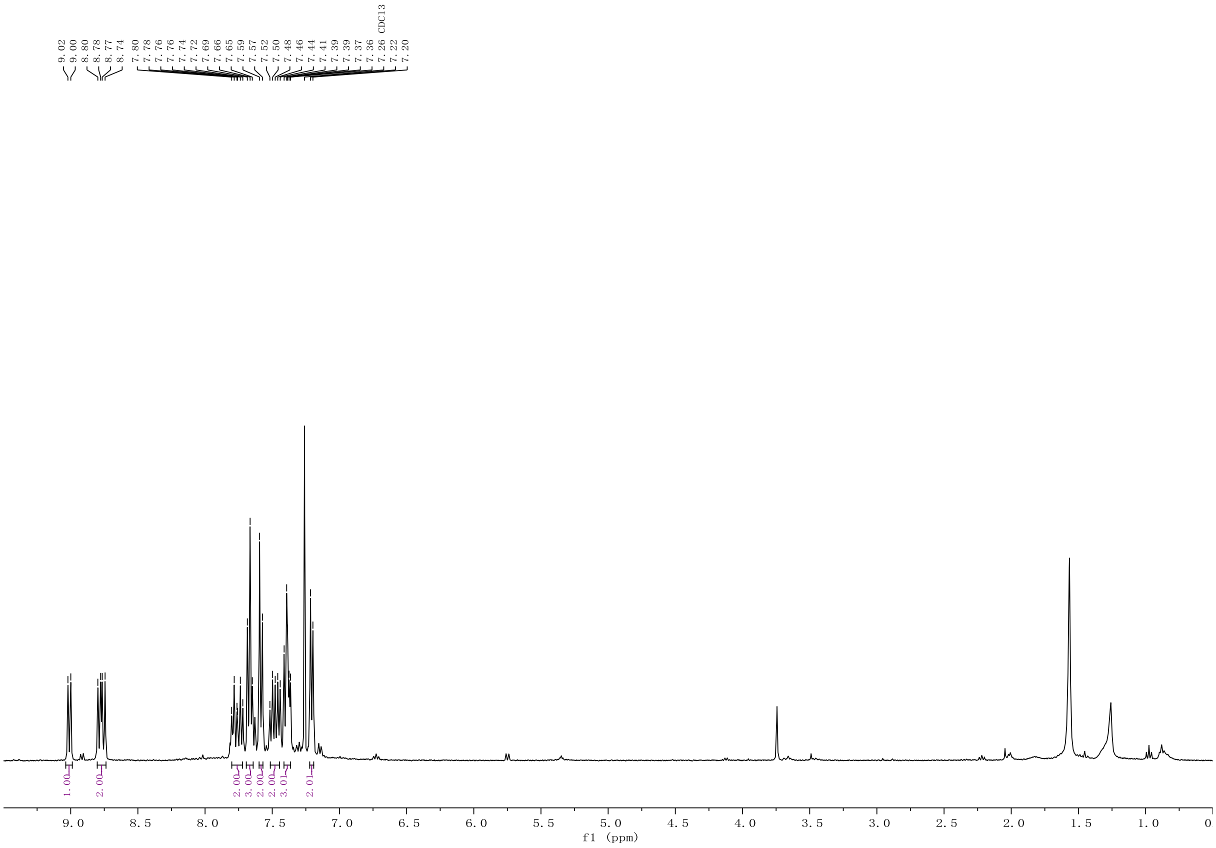

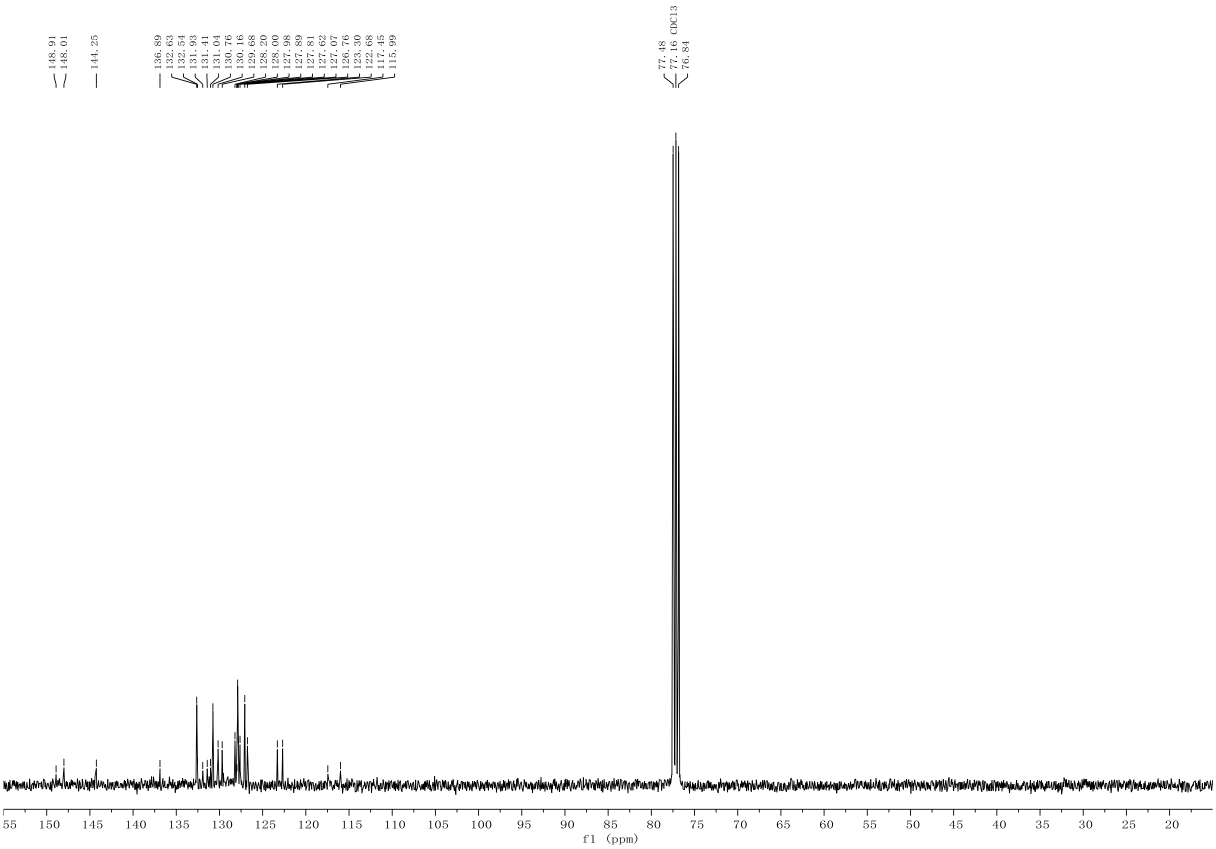

3al ^{1}H NMR (400MHz) and ^{13}C NMR (101MHz)

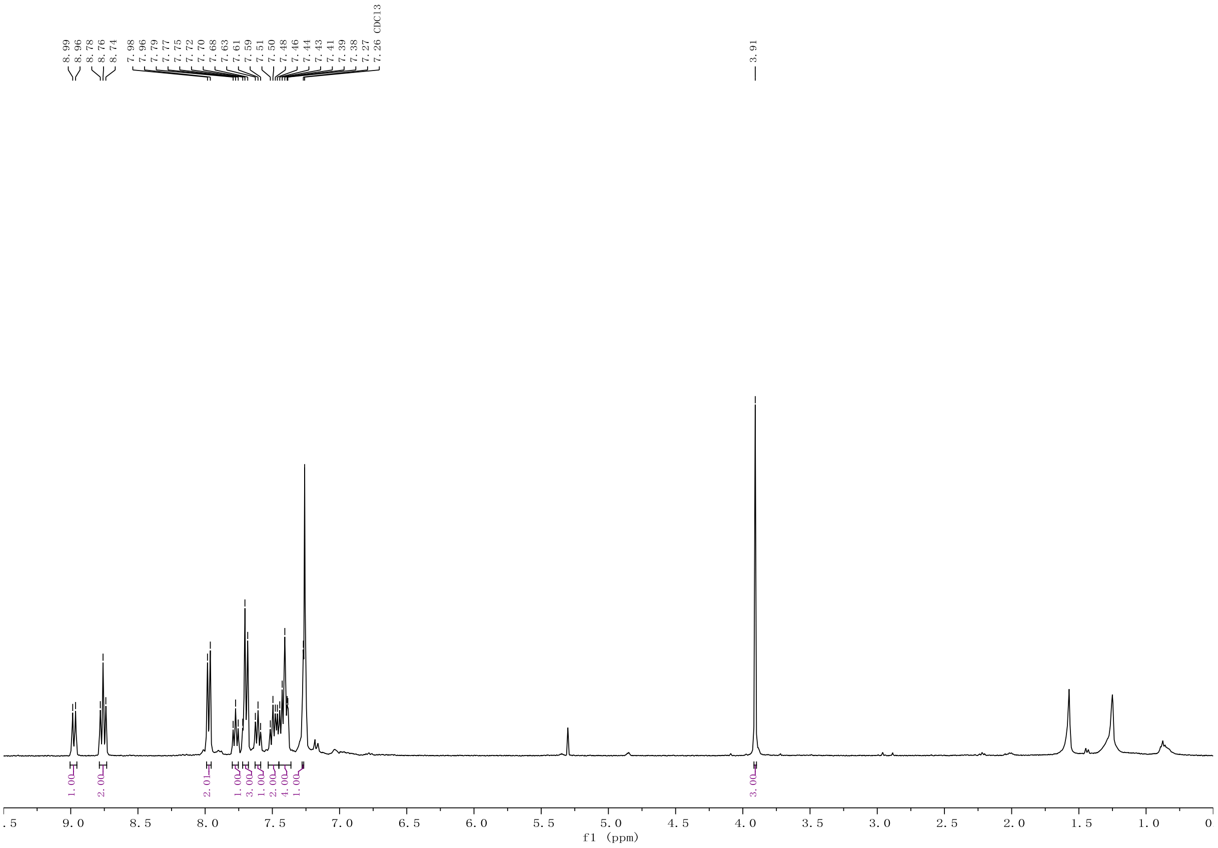

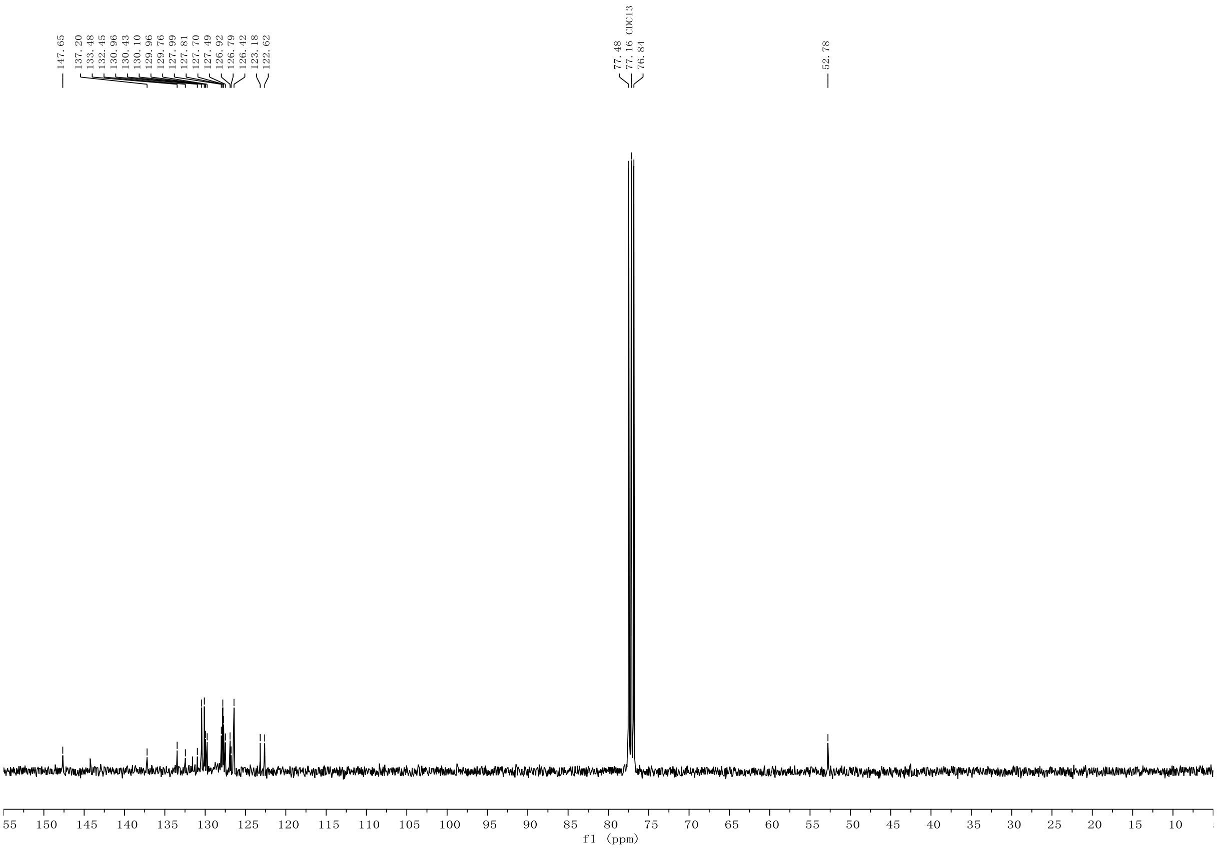

3am ^{1}H NMR (400MHz) and ^{13}C NMR (101MHz)

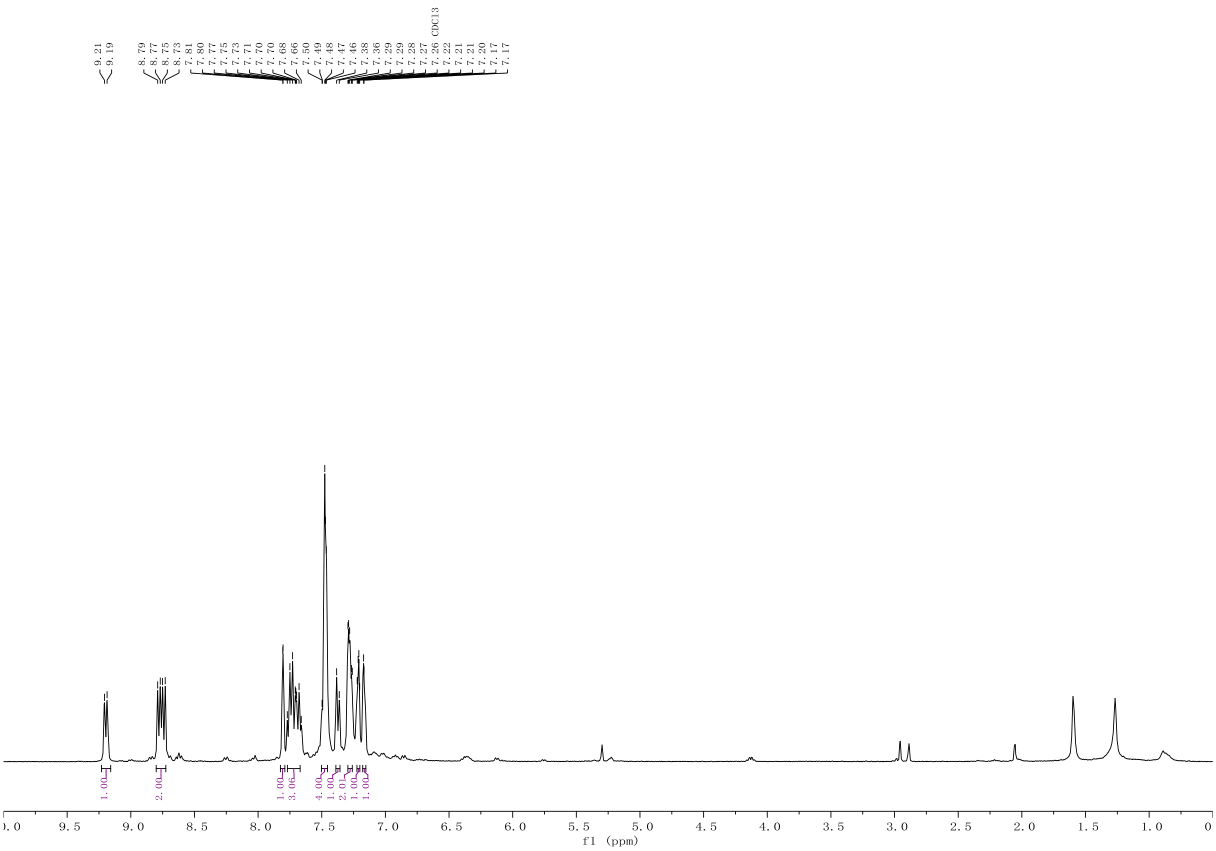

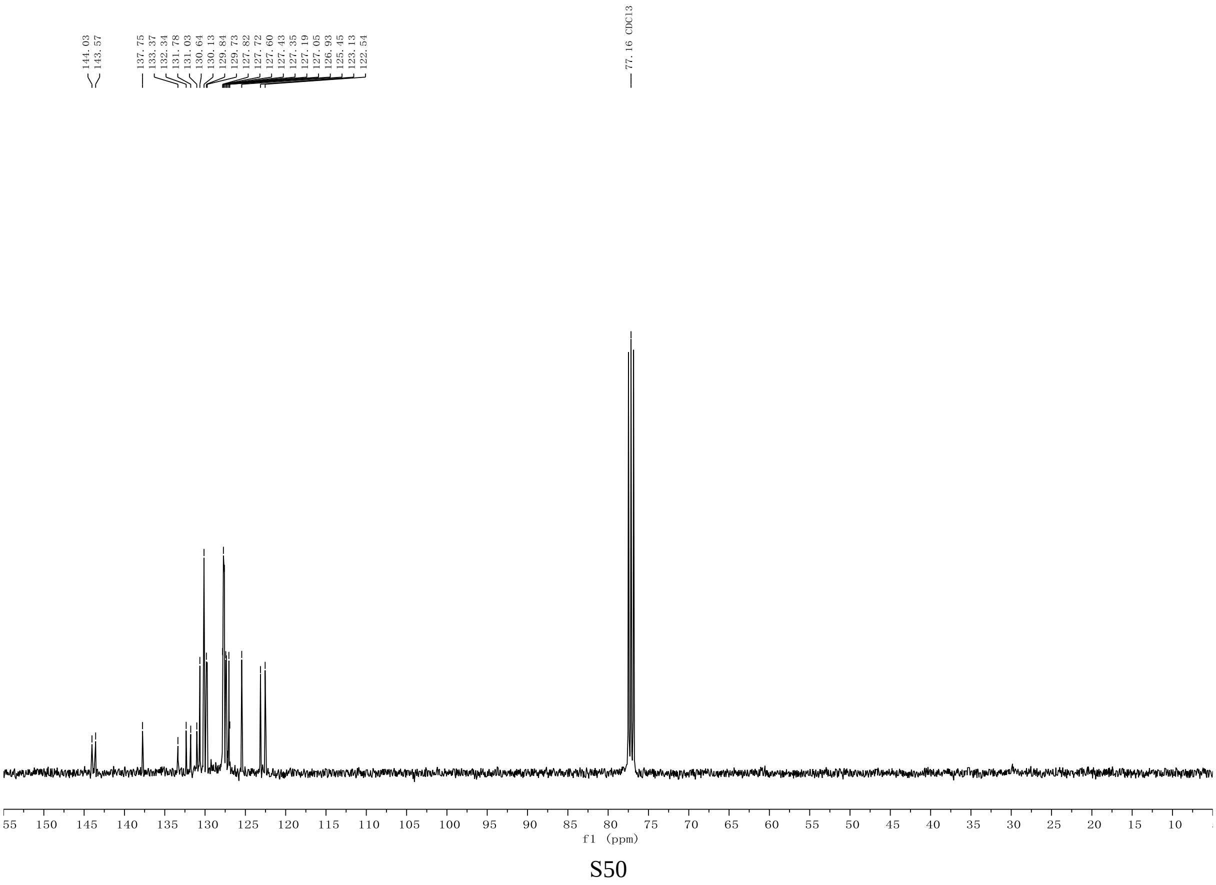

3an ^{1}H NMR (400MHz) and ^{13}C NMR (101MHz)

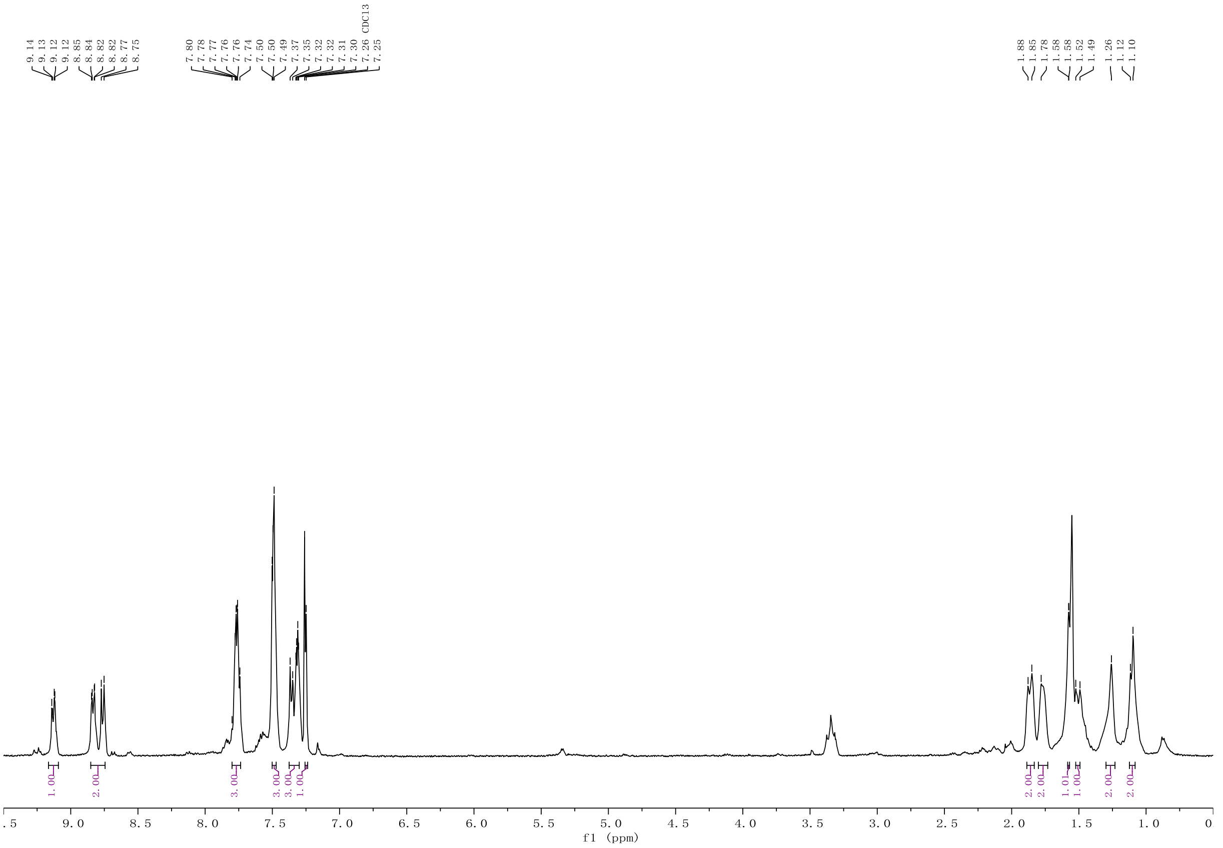

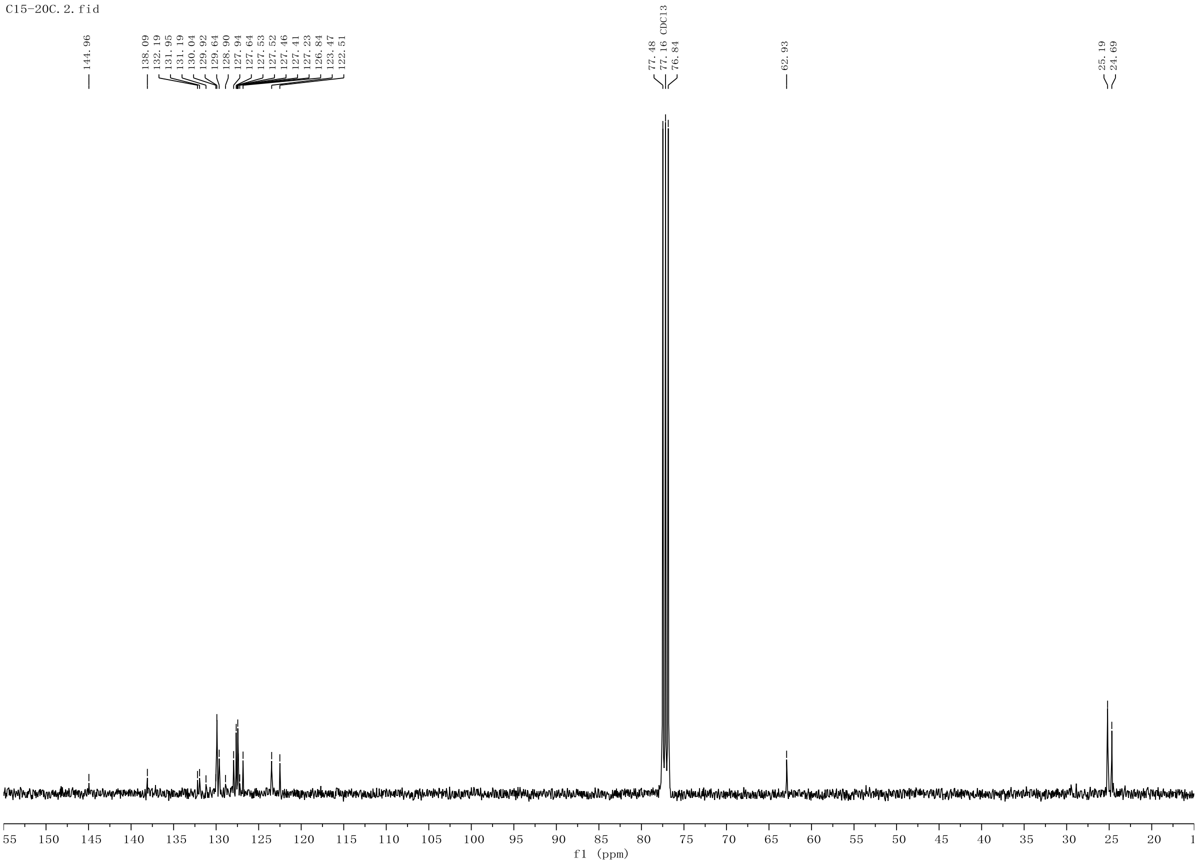

3ao ^{1}H NMR (400MHz) and ^{13}C NMR (101MHz**)**

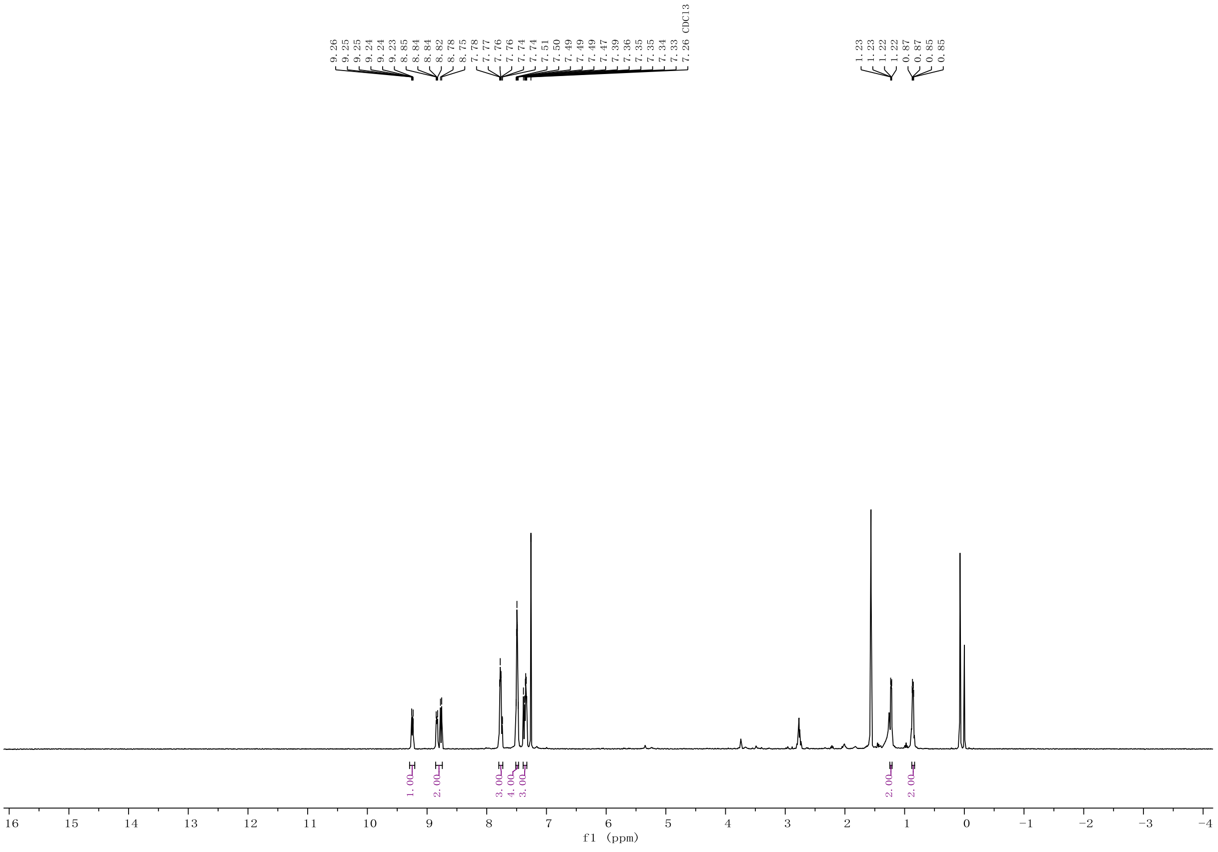

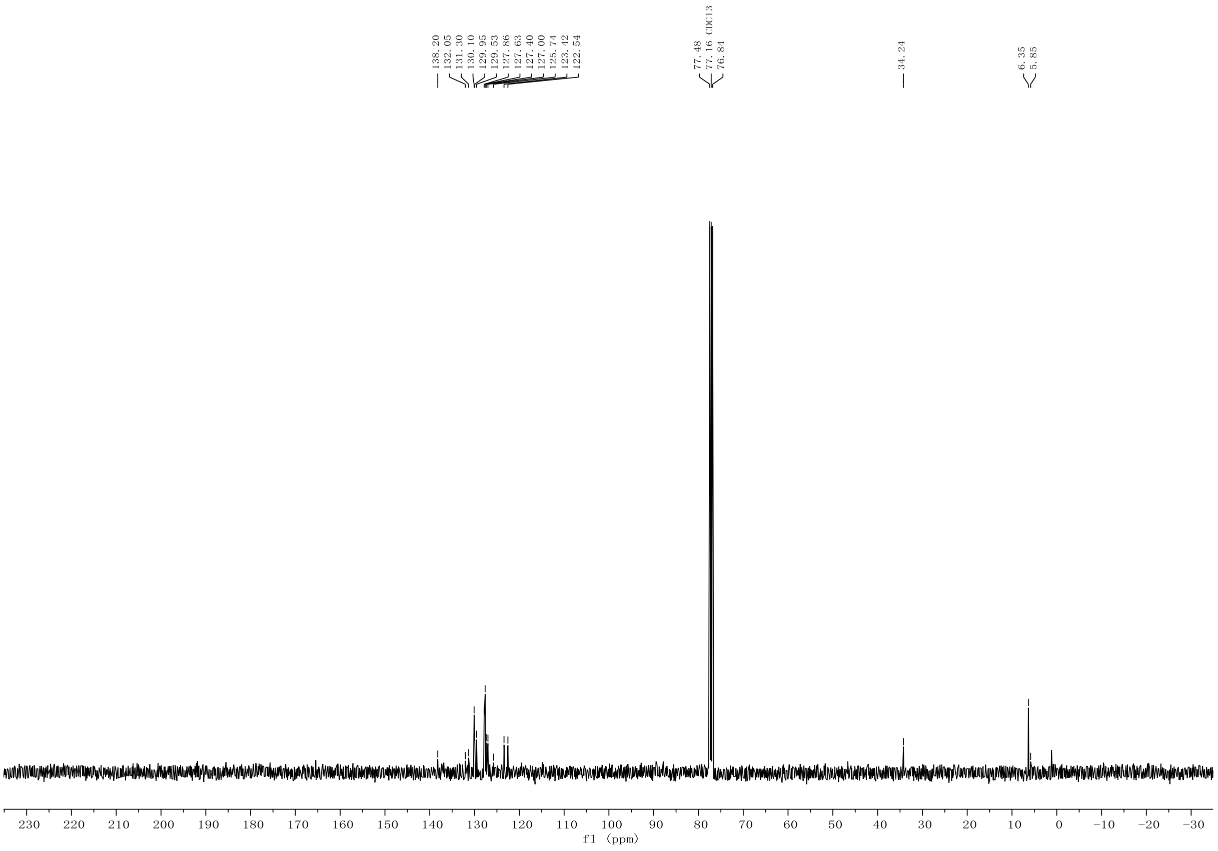

3ap ^{1}H NMR (400MHz) and ^{13}C NMR (101MHz)

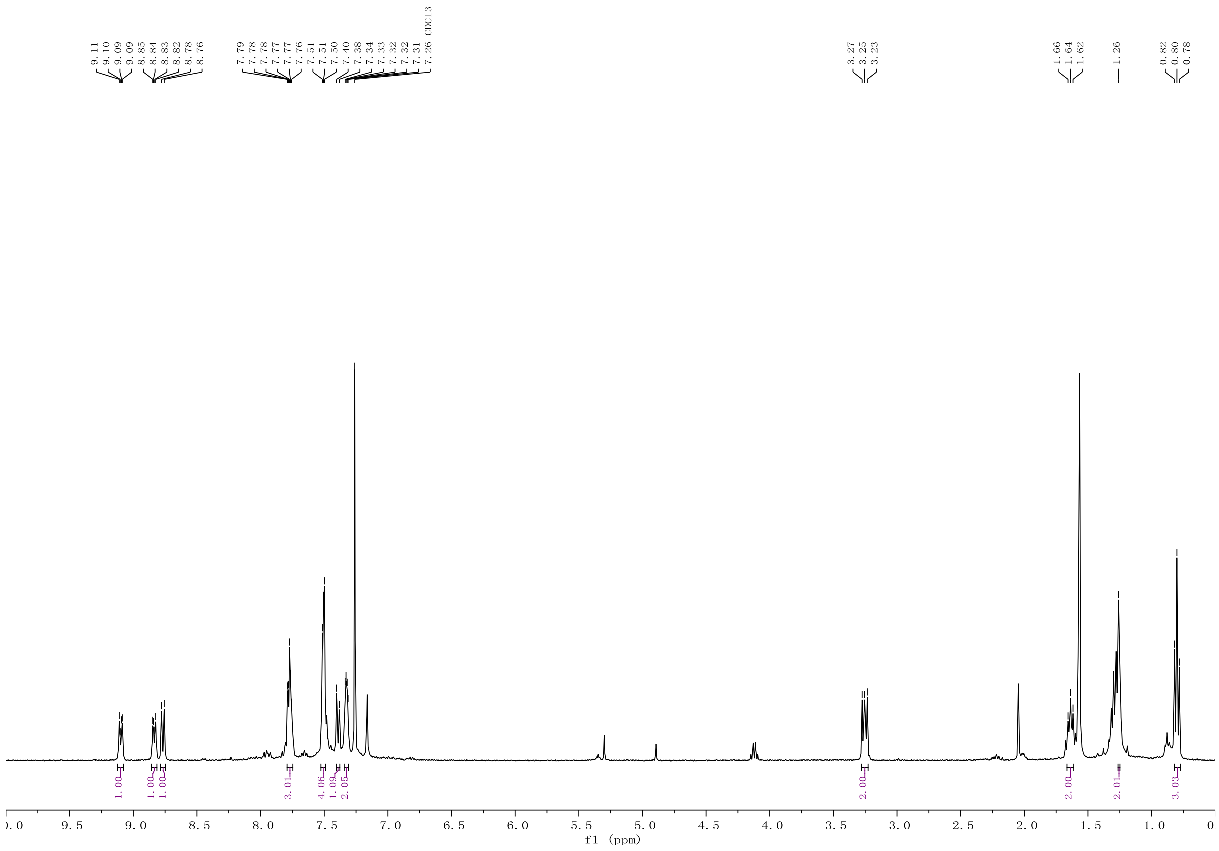

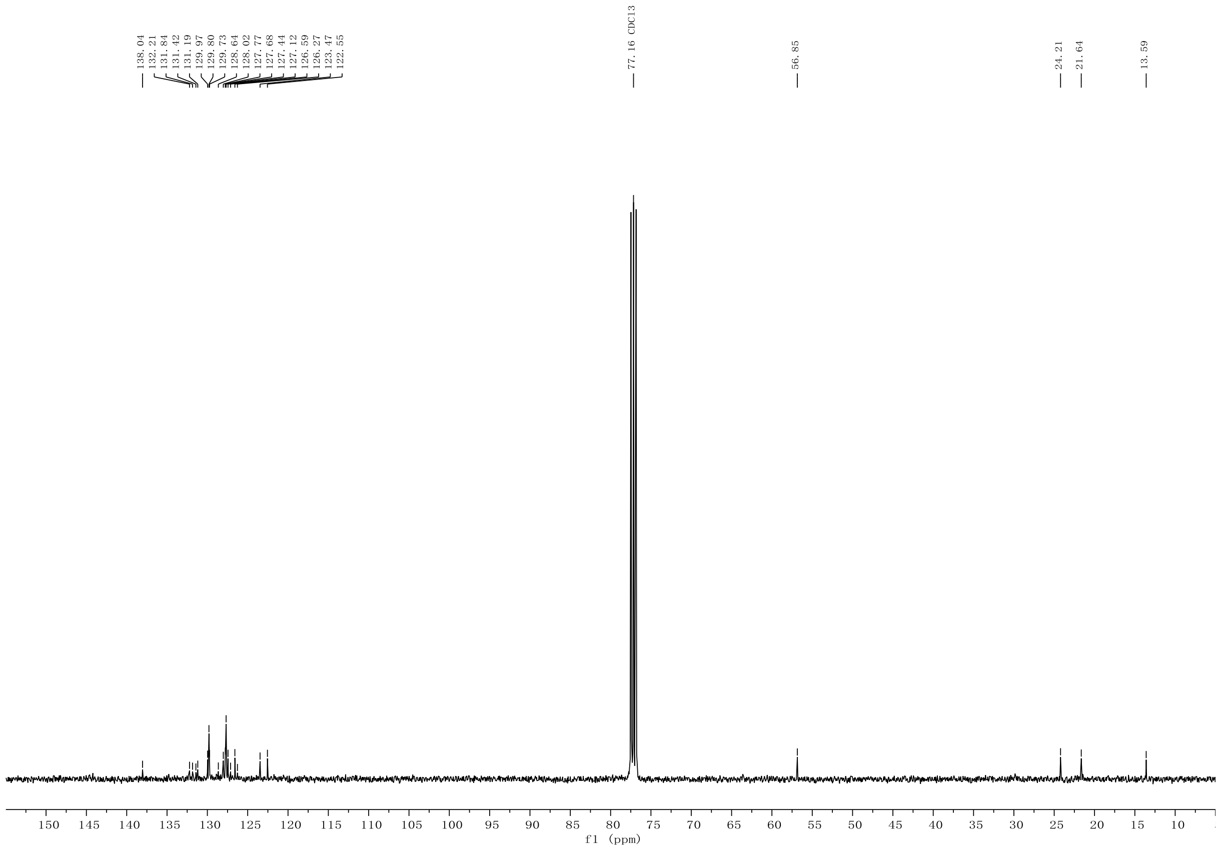

3ba ^{1}H NMR (500MHz) and ^{13}C NMR (126MHz)

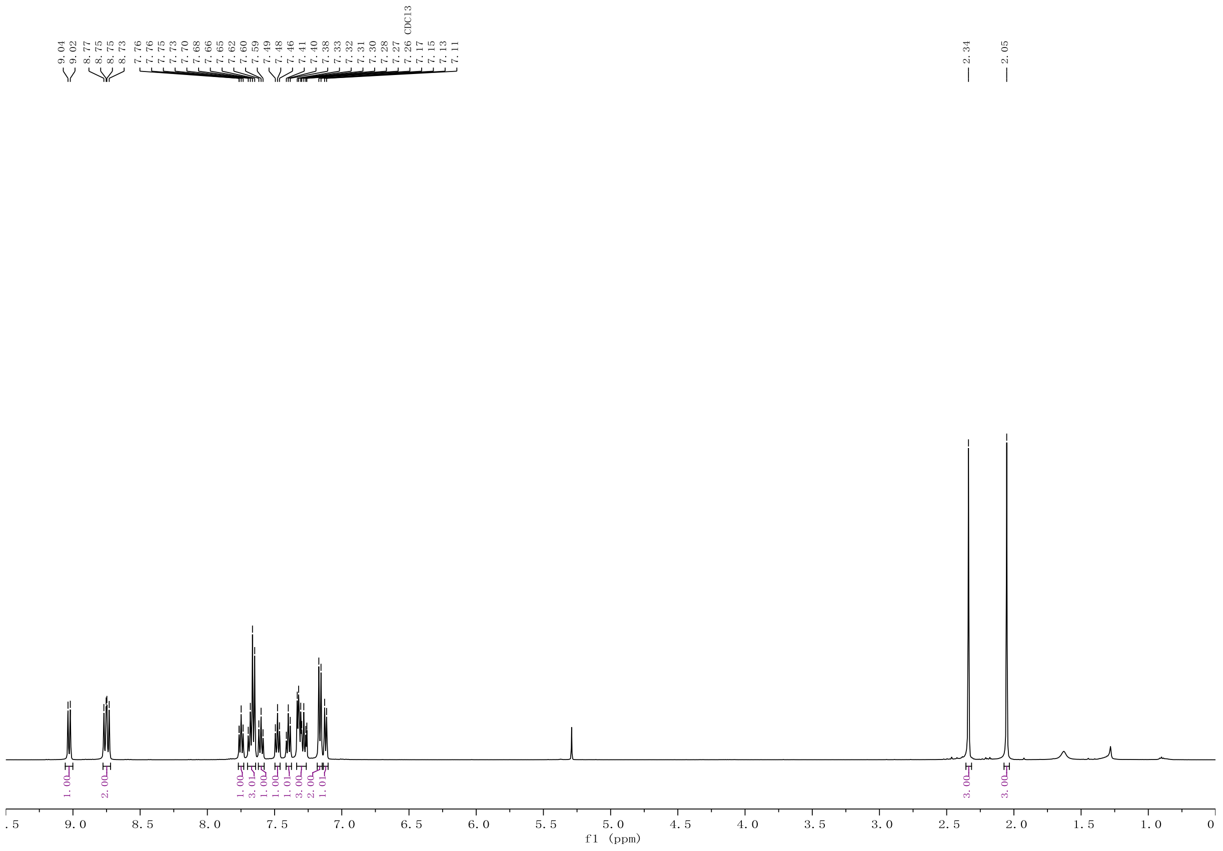

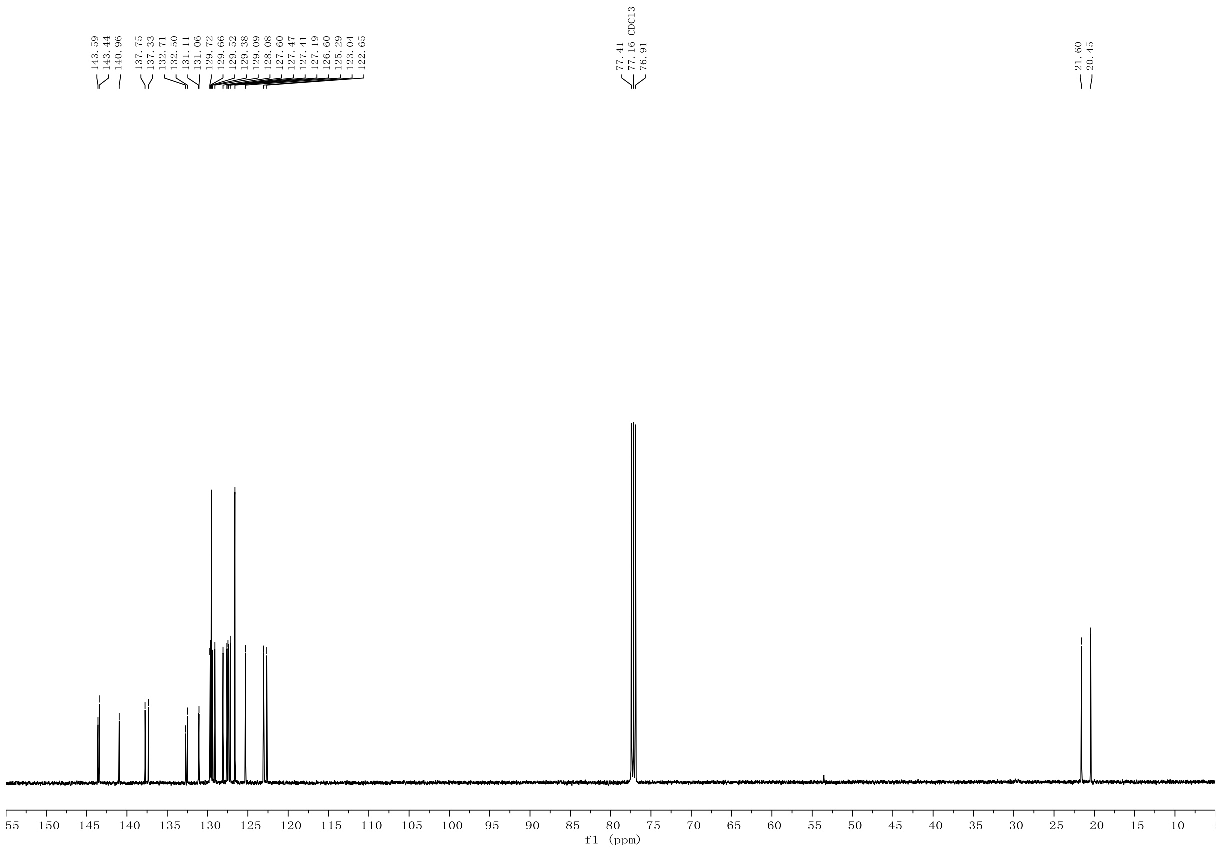

3ca ^{1}H NMR (500MHz) and ^{13}C NMR (126MHz)

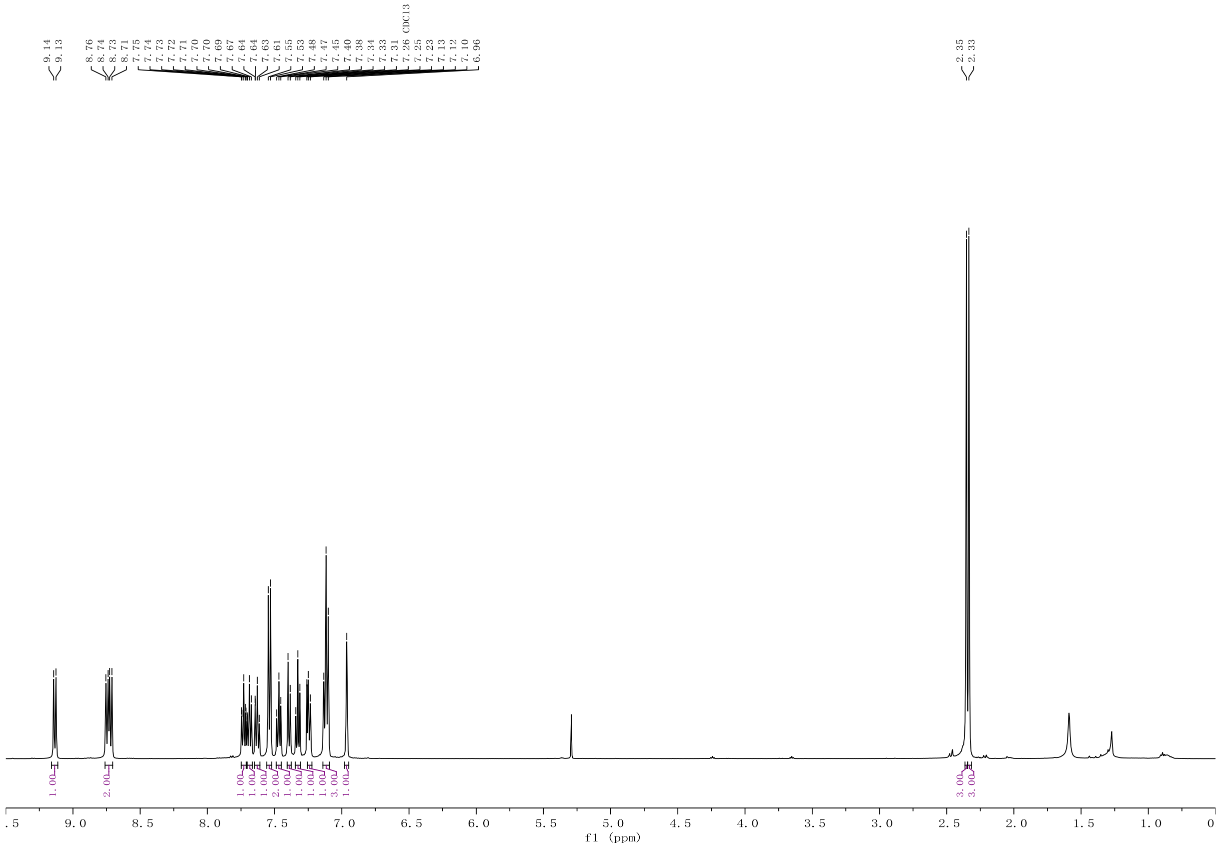

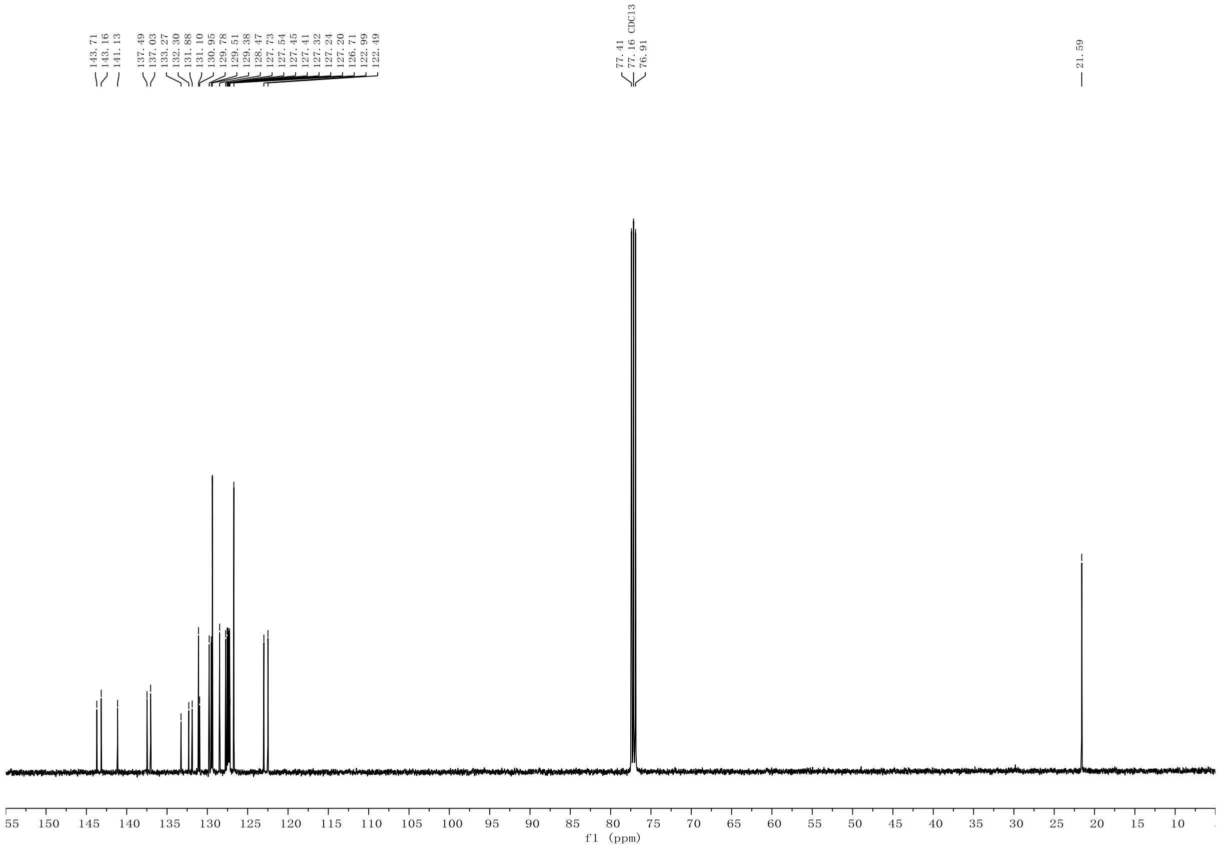

3da ^{1}H NMR (500MHz) and ^{13}C NMR (126MHz)

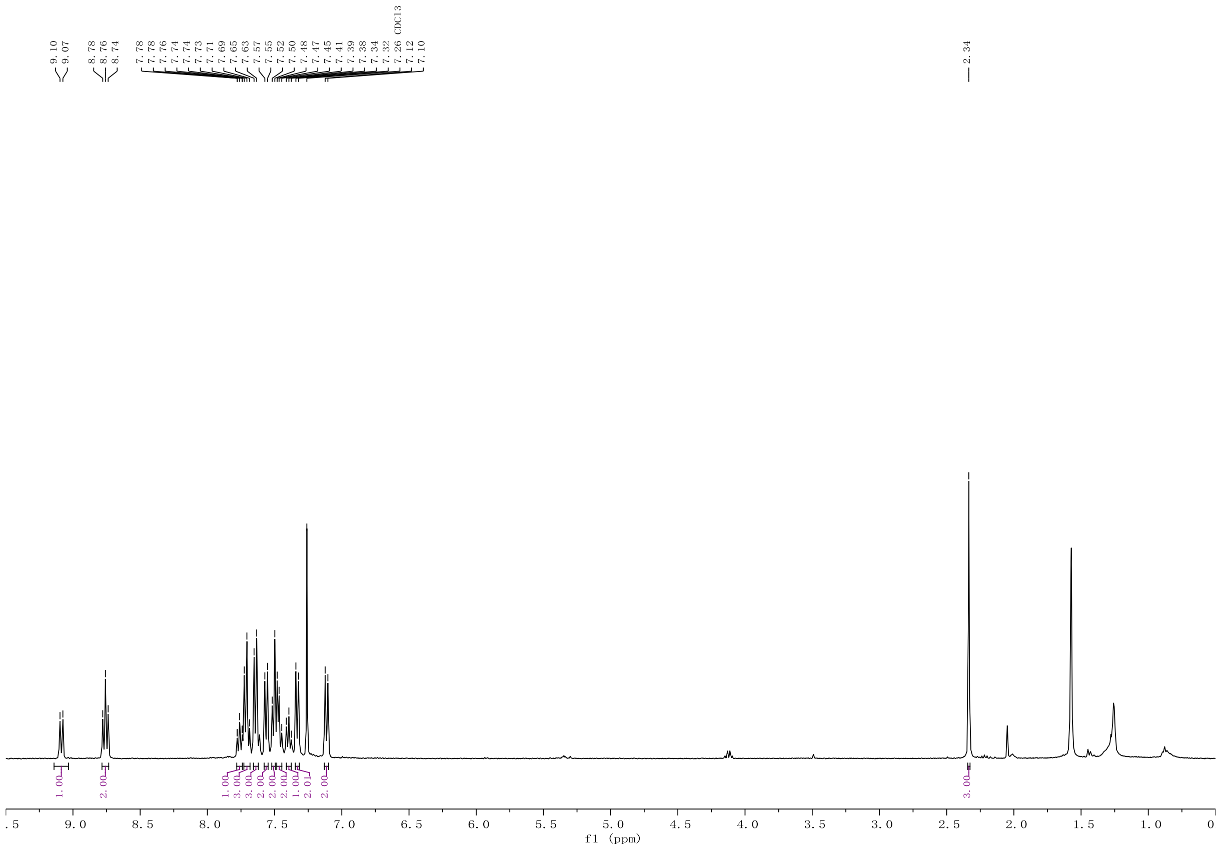

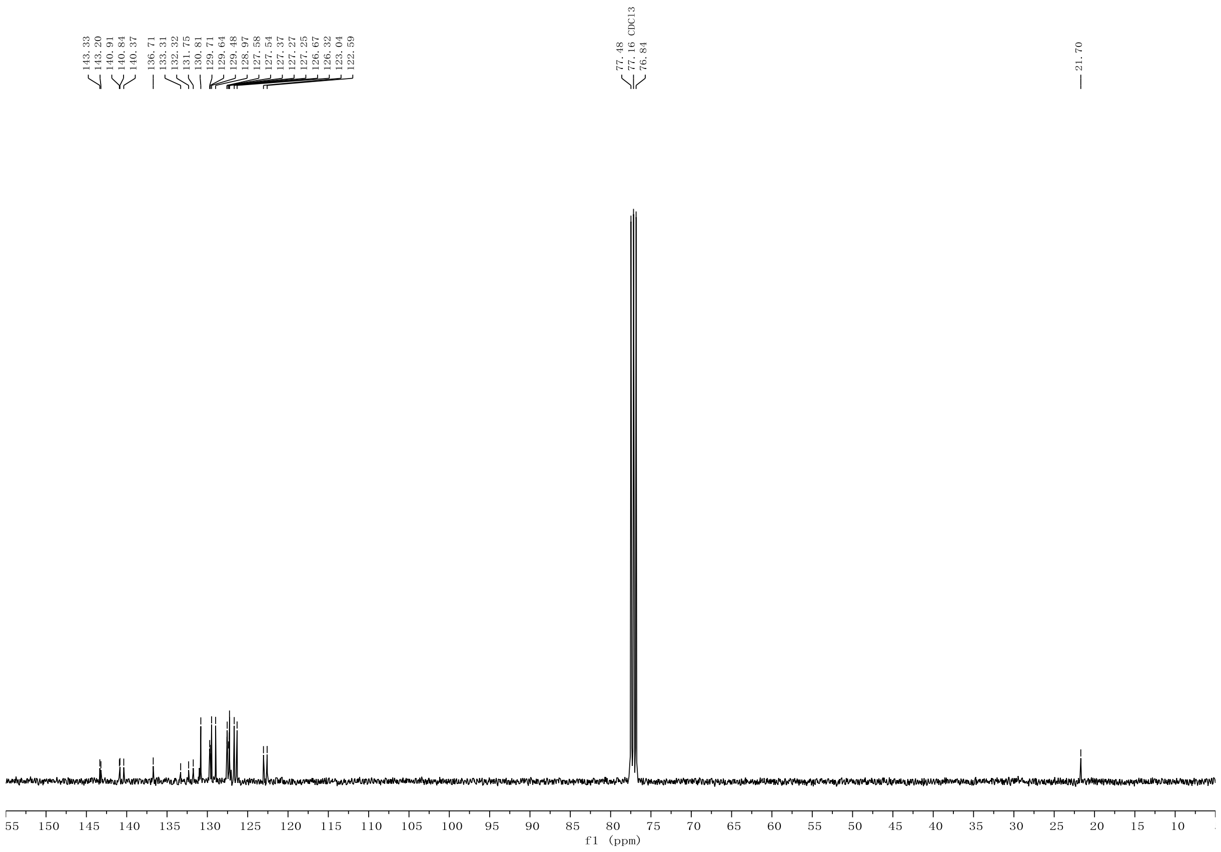

3ea ^{1}H NMR (500MHz) and ^{13}C NMR (126MHz)

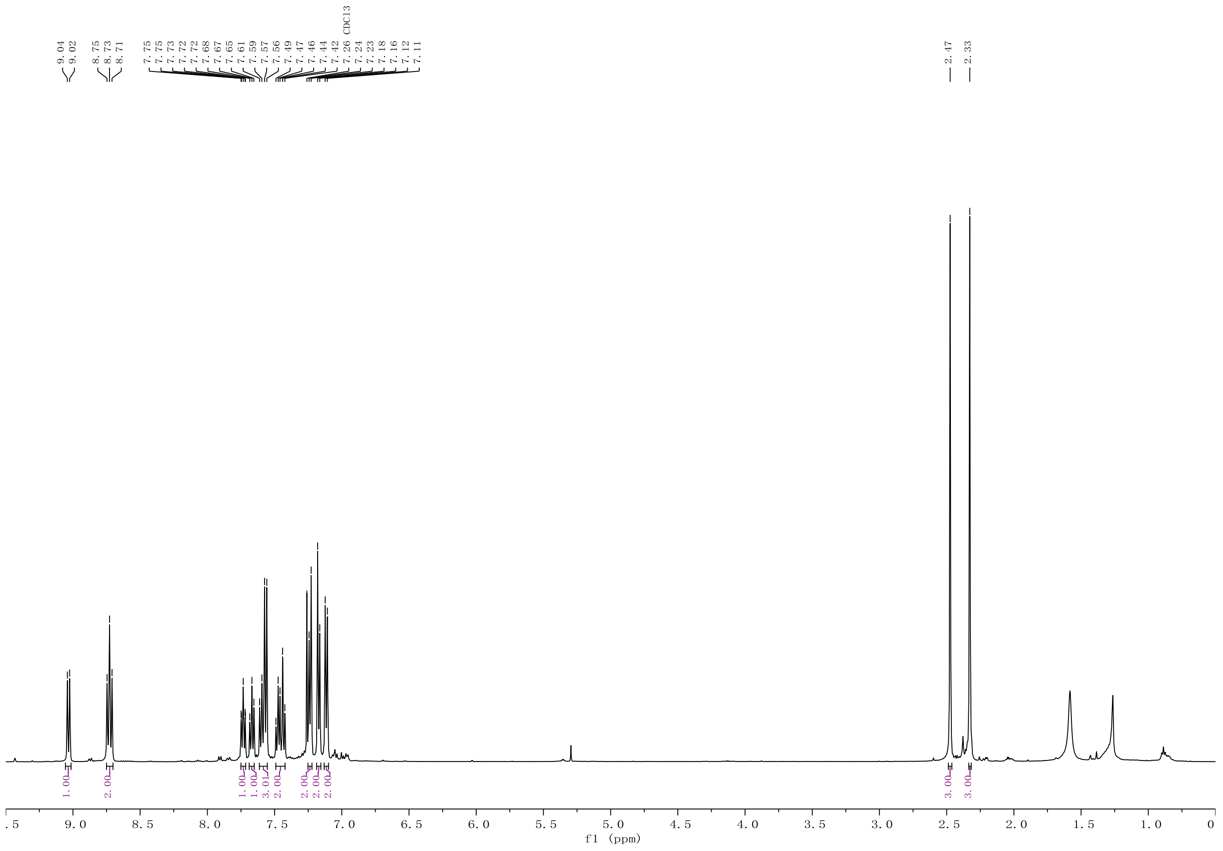

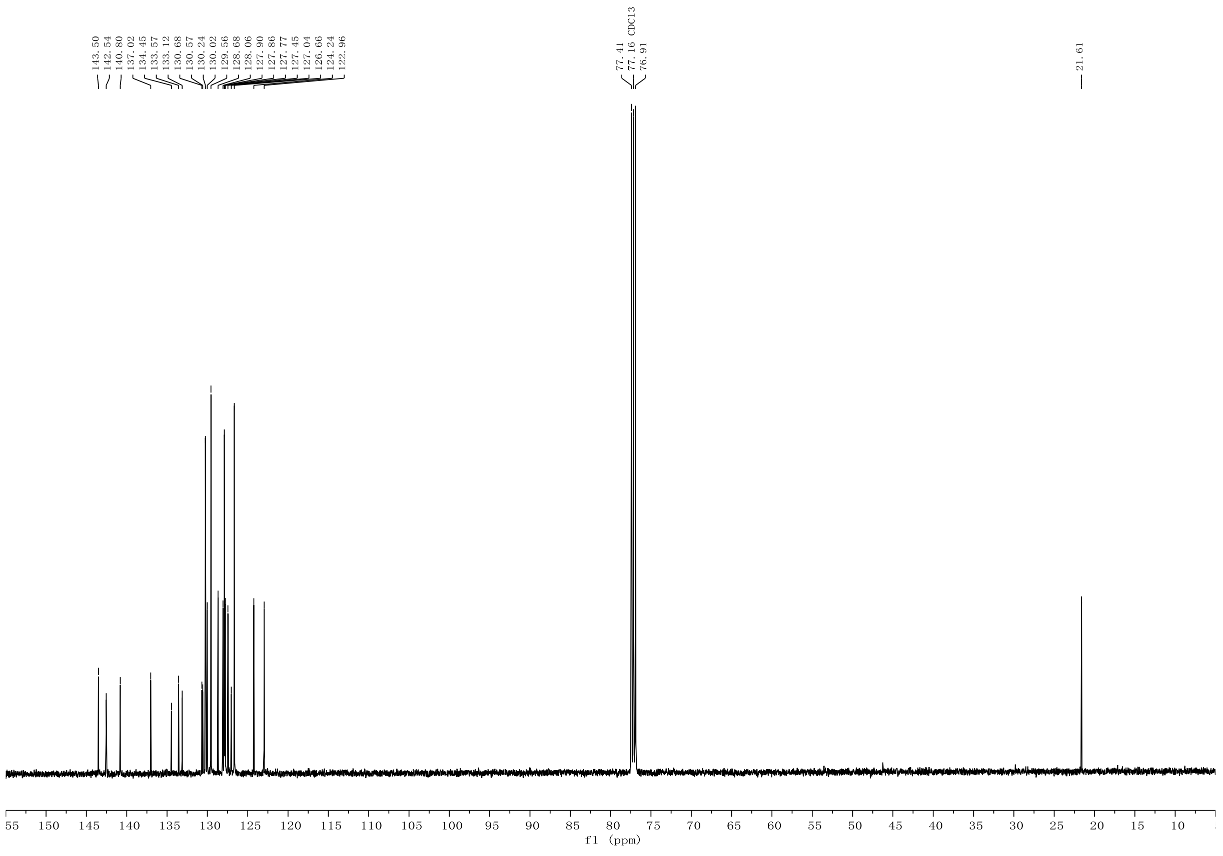

3fa ^{1}H NMR (500MHz) and ^{13}C NMR (126MHz)

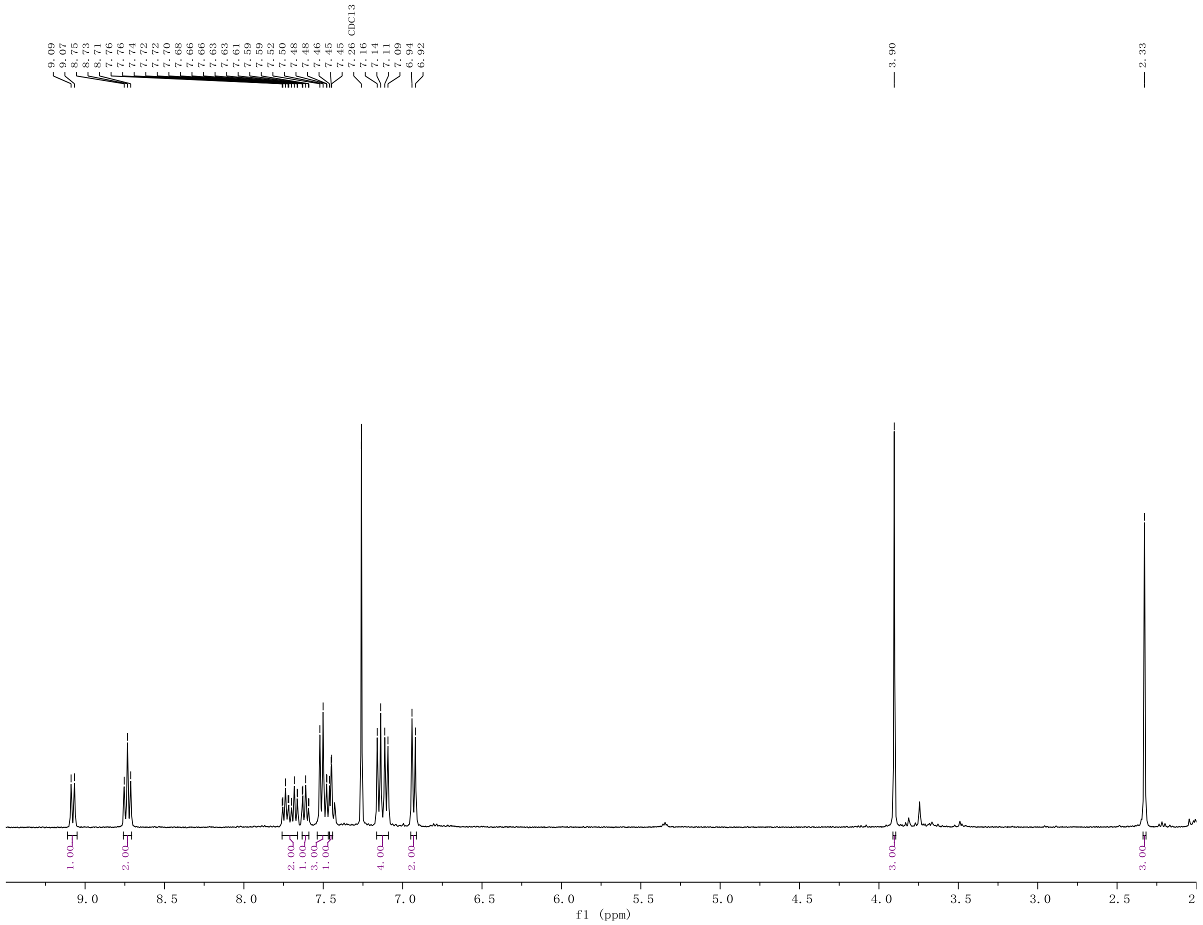

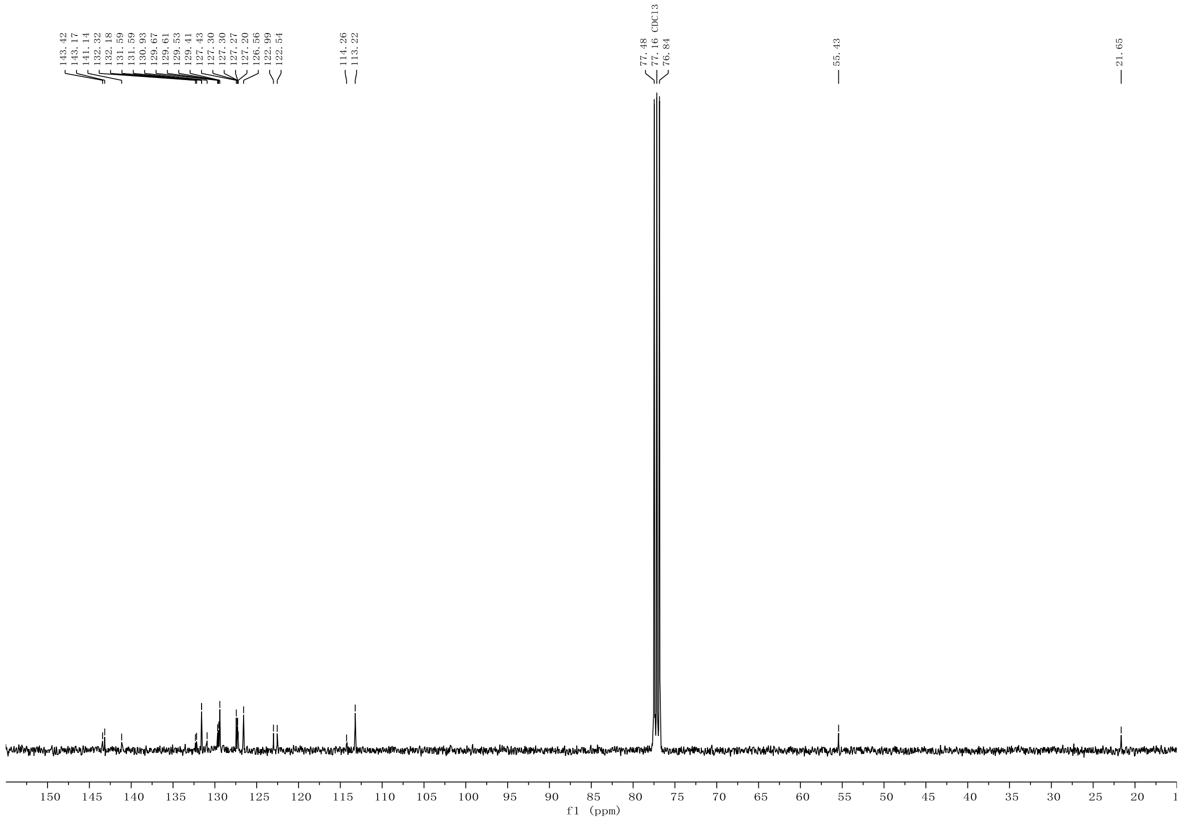

3ga ^{1}H NMR (500MHz), ^{13}C NMR (126MHz**)** and ^{19}F NMR (471 MHz)

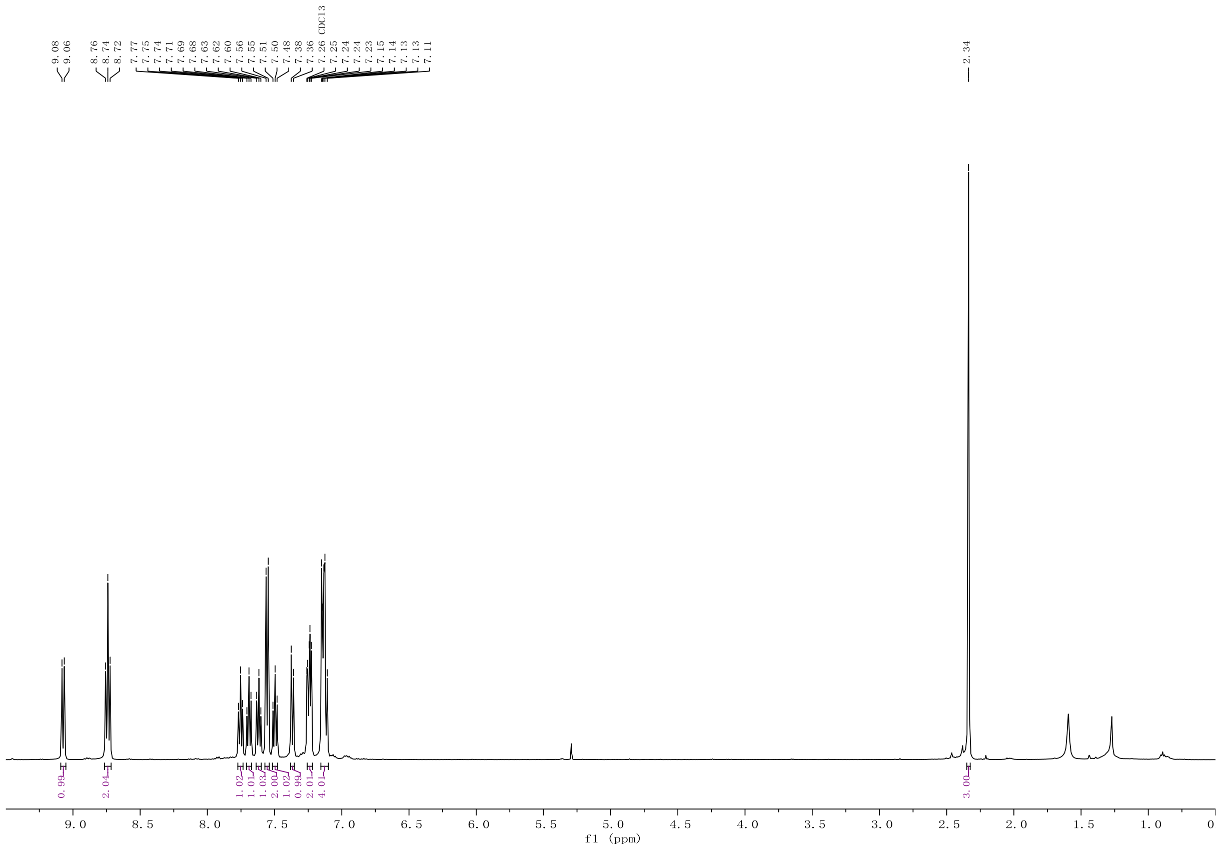

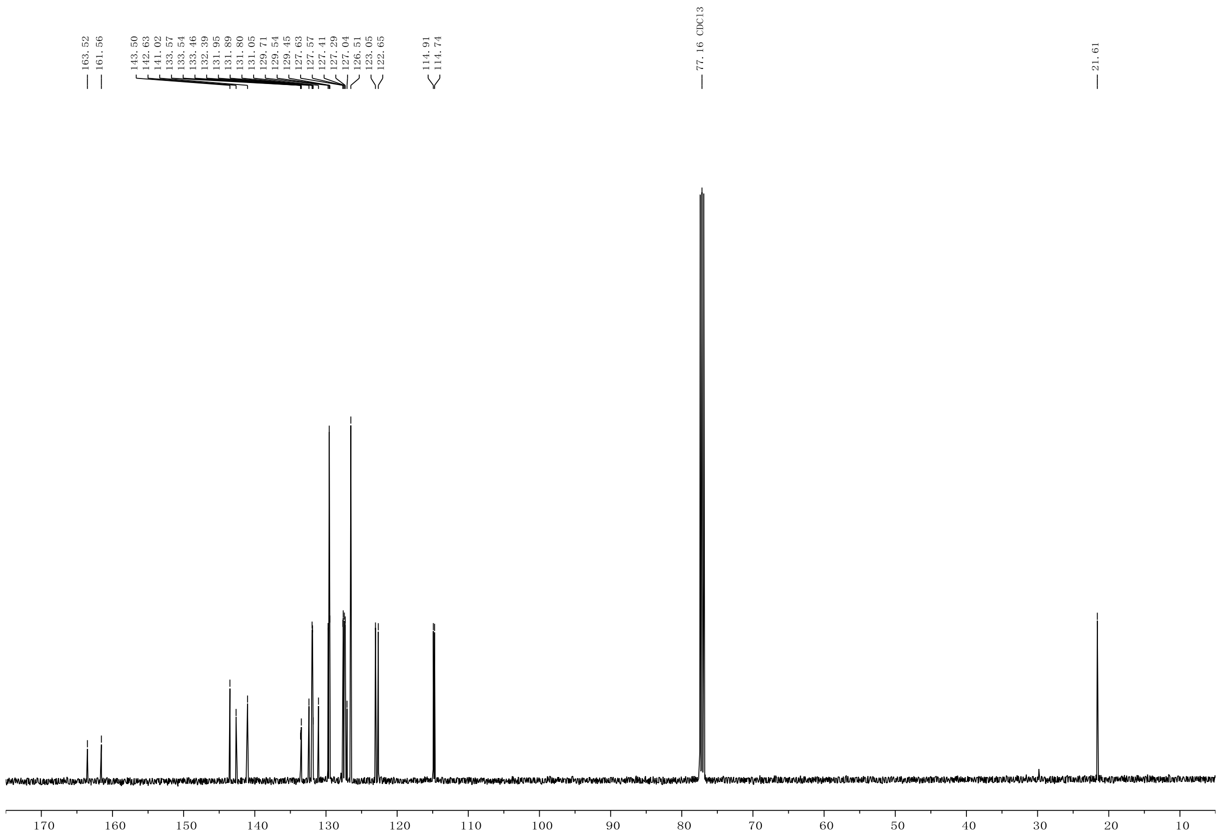

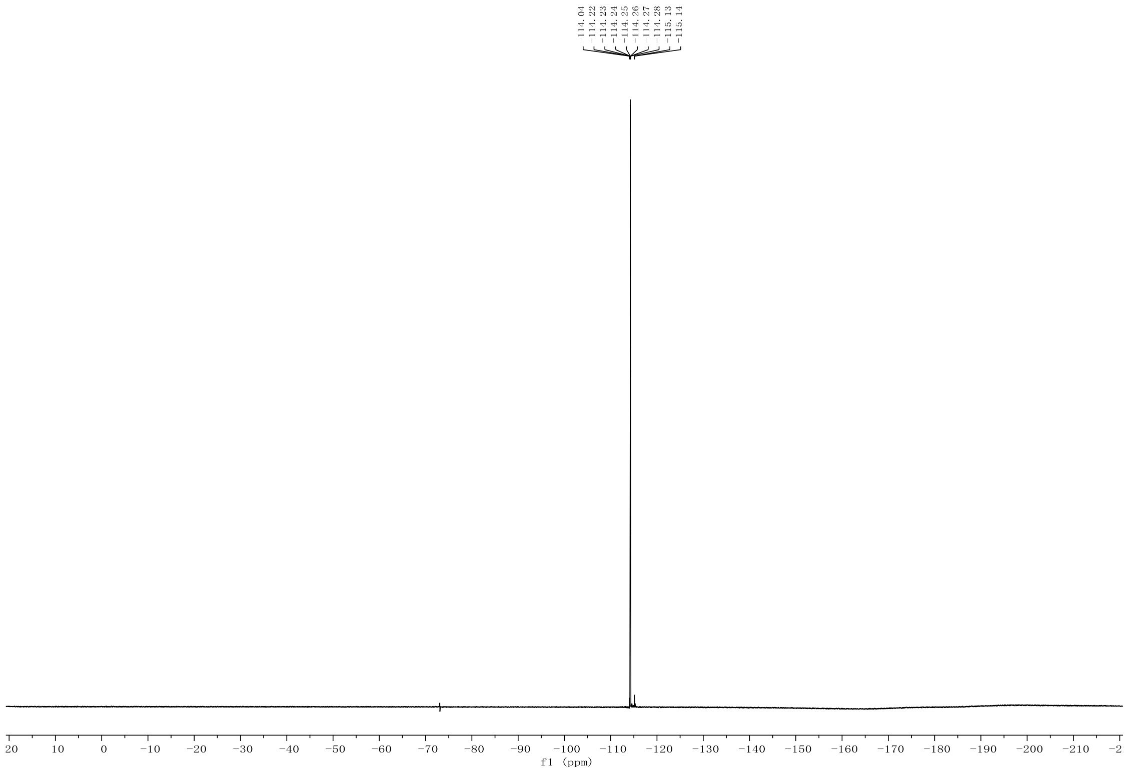

-114.04
-114.22
-114.23
-114.24
-114.25
-114.26
-114.27
-114.28
-115.13
-115.14
20 10 0 -10 -20 -30 -40 -50 -60 -70 -80 -90 -100 -110 -120 -130 -140 -150 -160 -170 -180 -190 -200 -210 -2
f1 (ppm)

3ha ^{1}H NMR (500MHz) and ^{13}C NMR (126MHz)

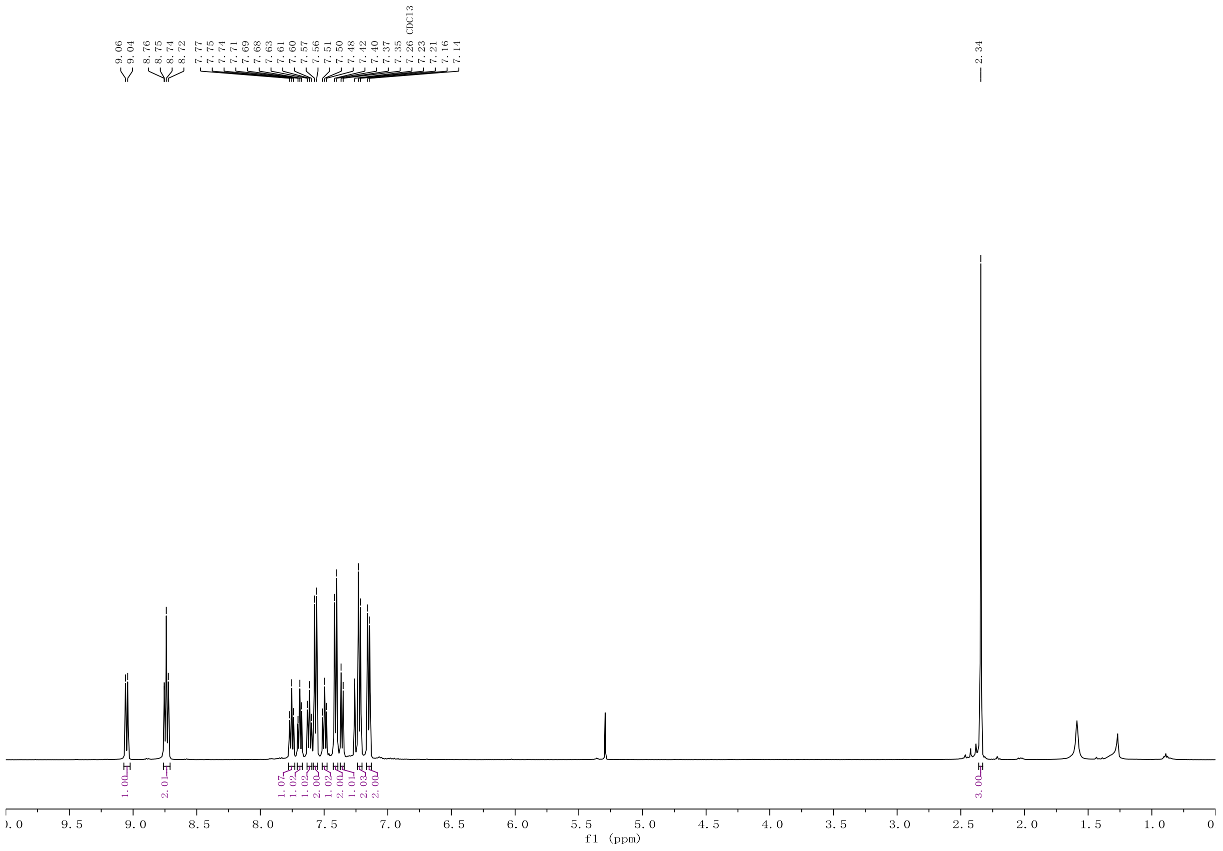

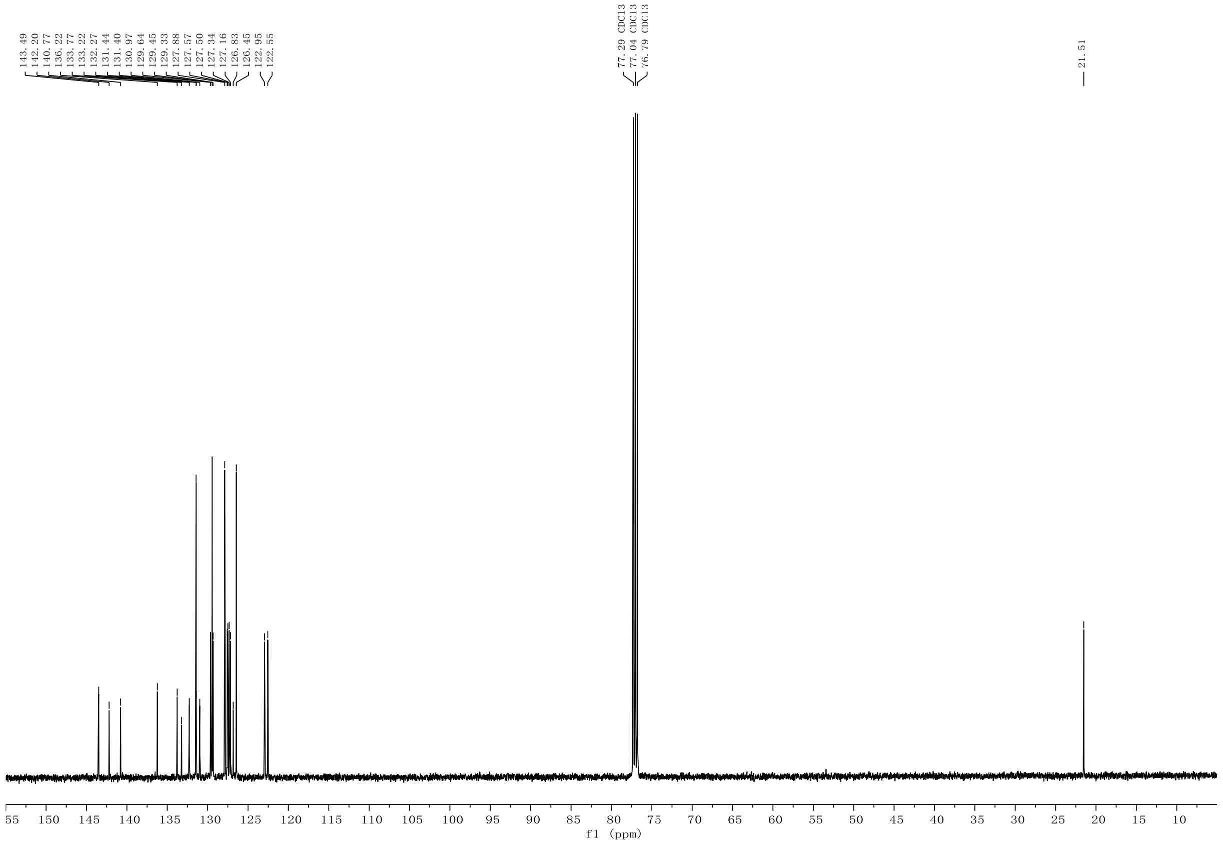

3ia ^{1}H NMR (500MHz) and ^{13}C NMR (126MHz)

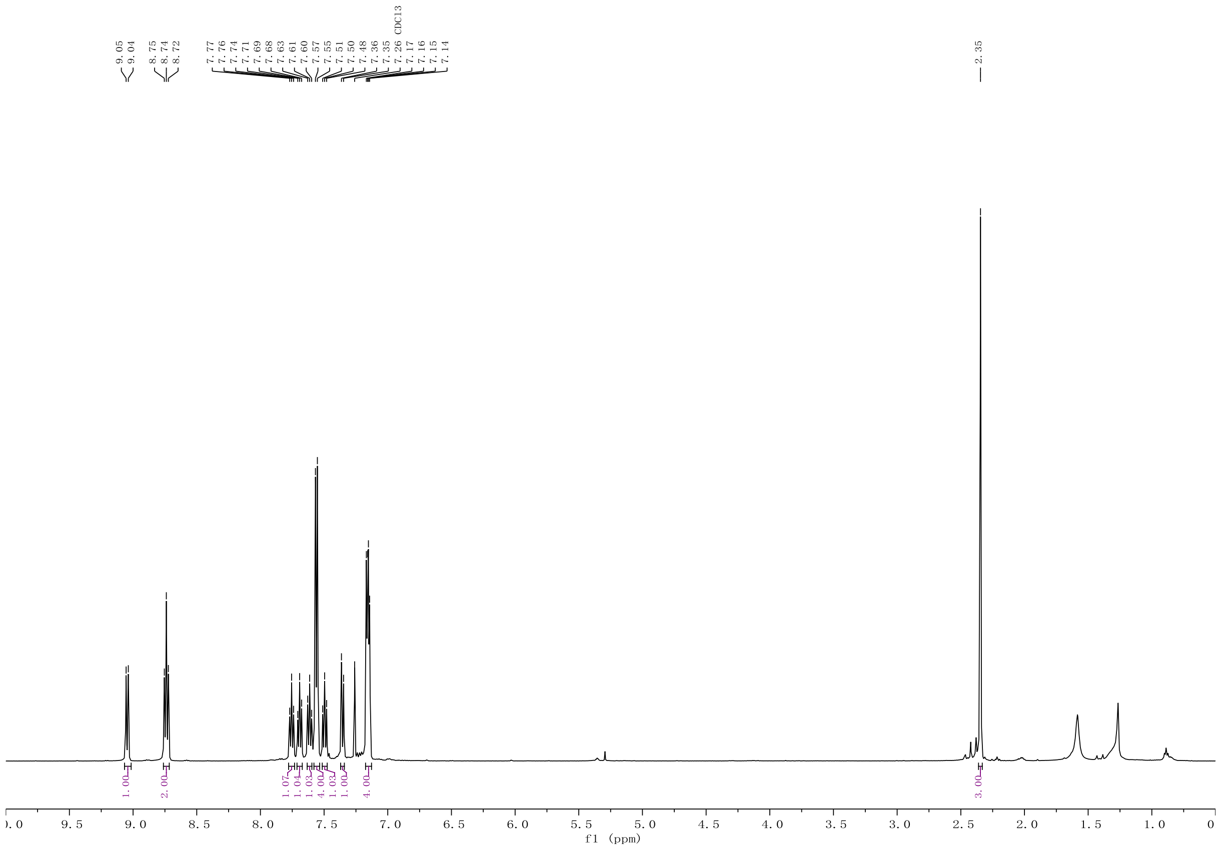

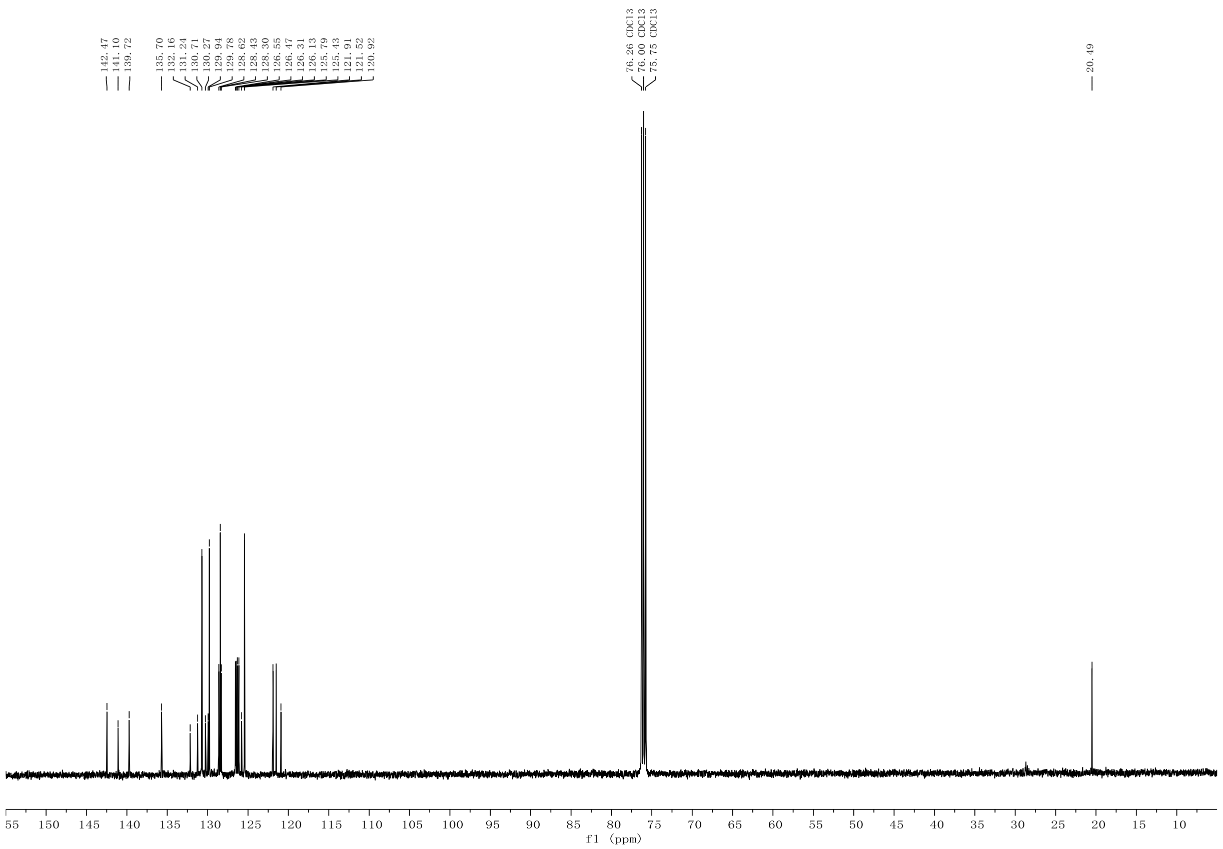

3ja ^{1}H NMR (500MHz) and ^{13}C NMR (126MHz)

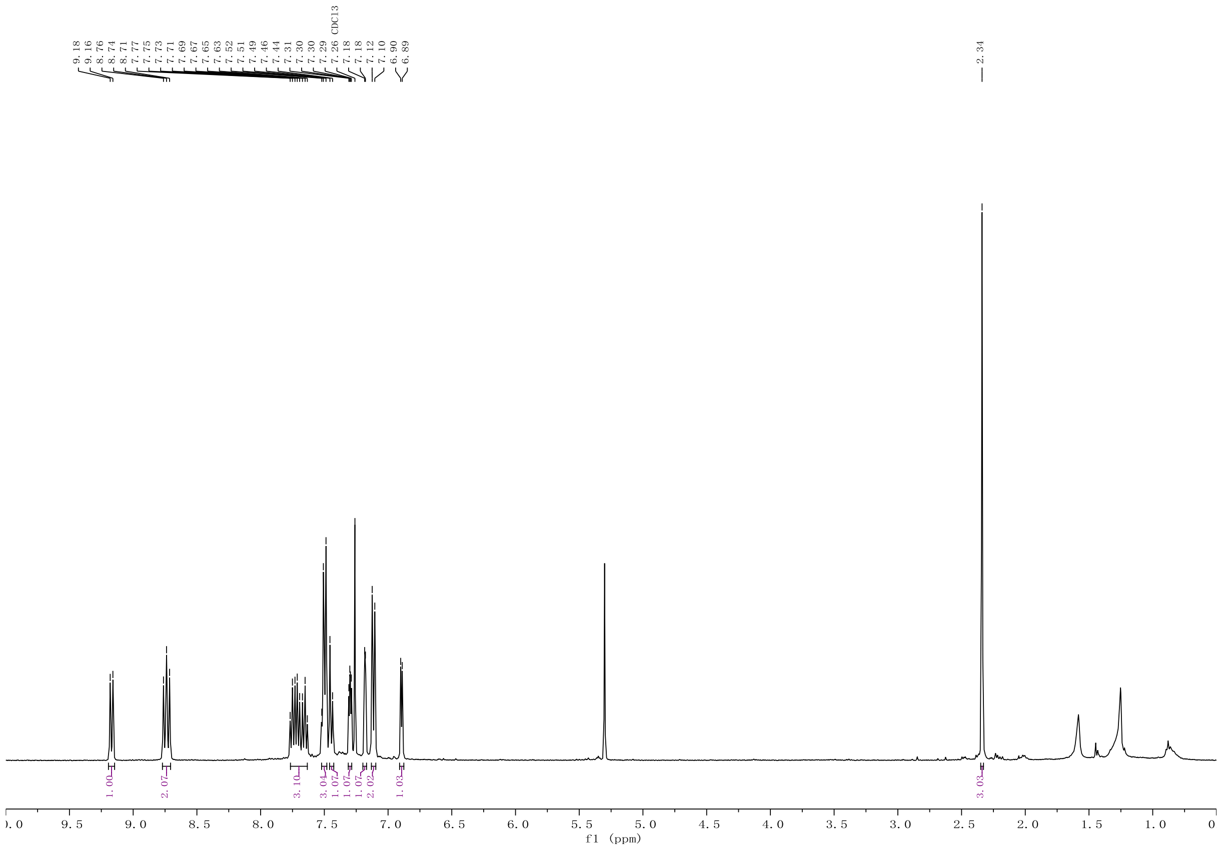

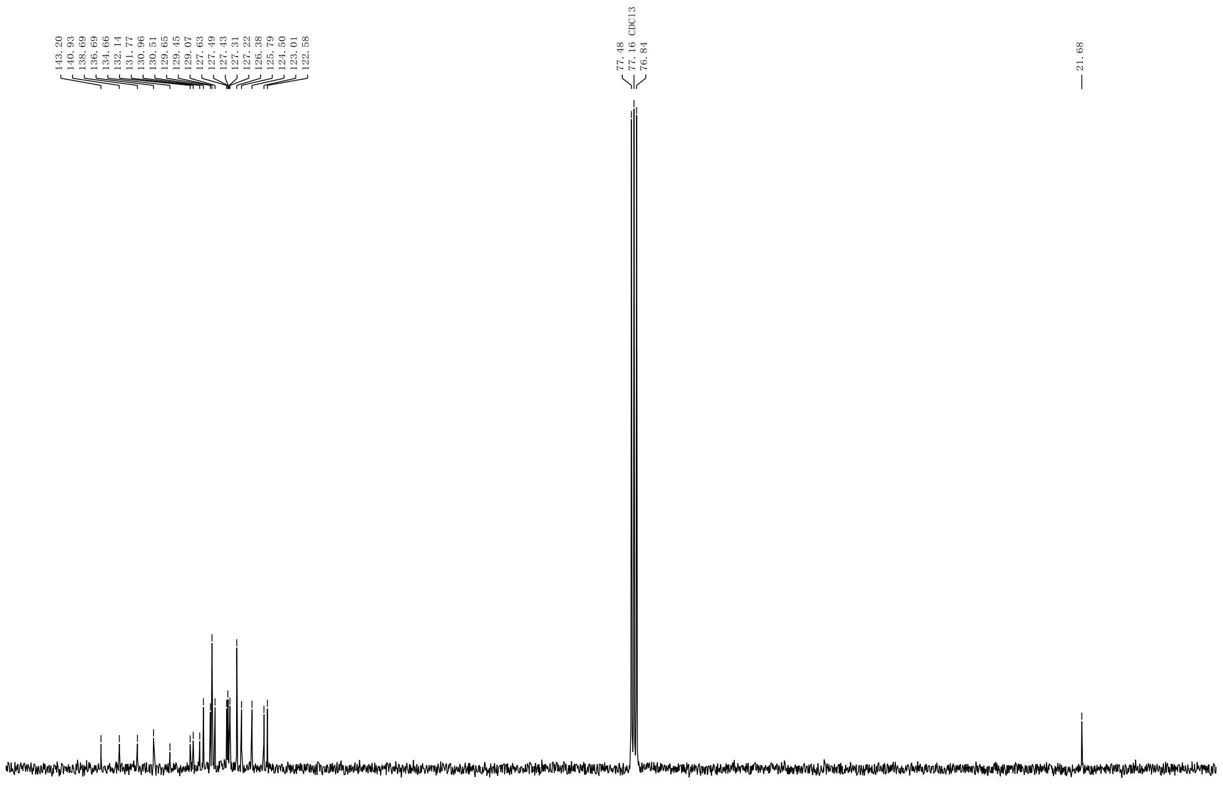

3ka ^{1}H NMR (500MHz) and ^{13}C NMR (126MHz)

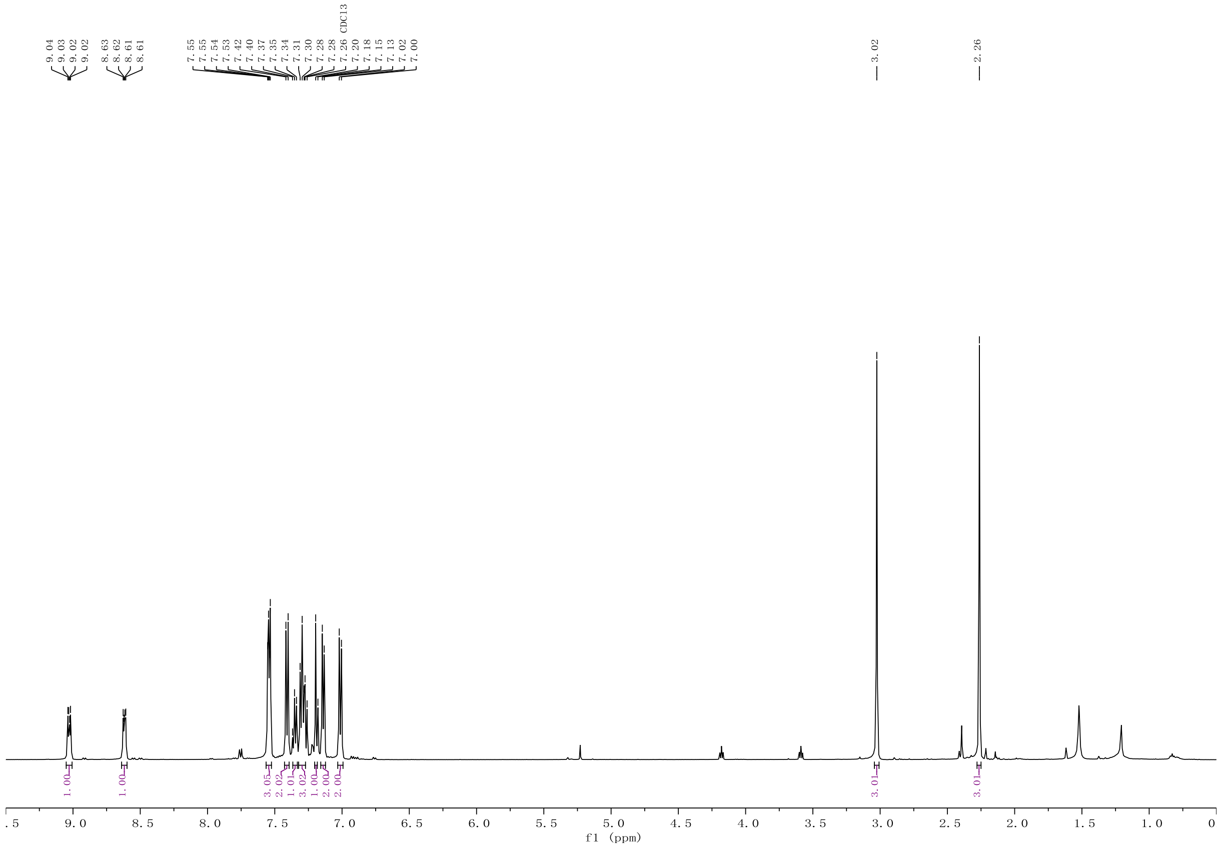

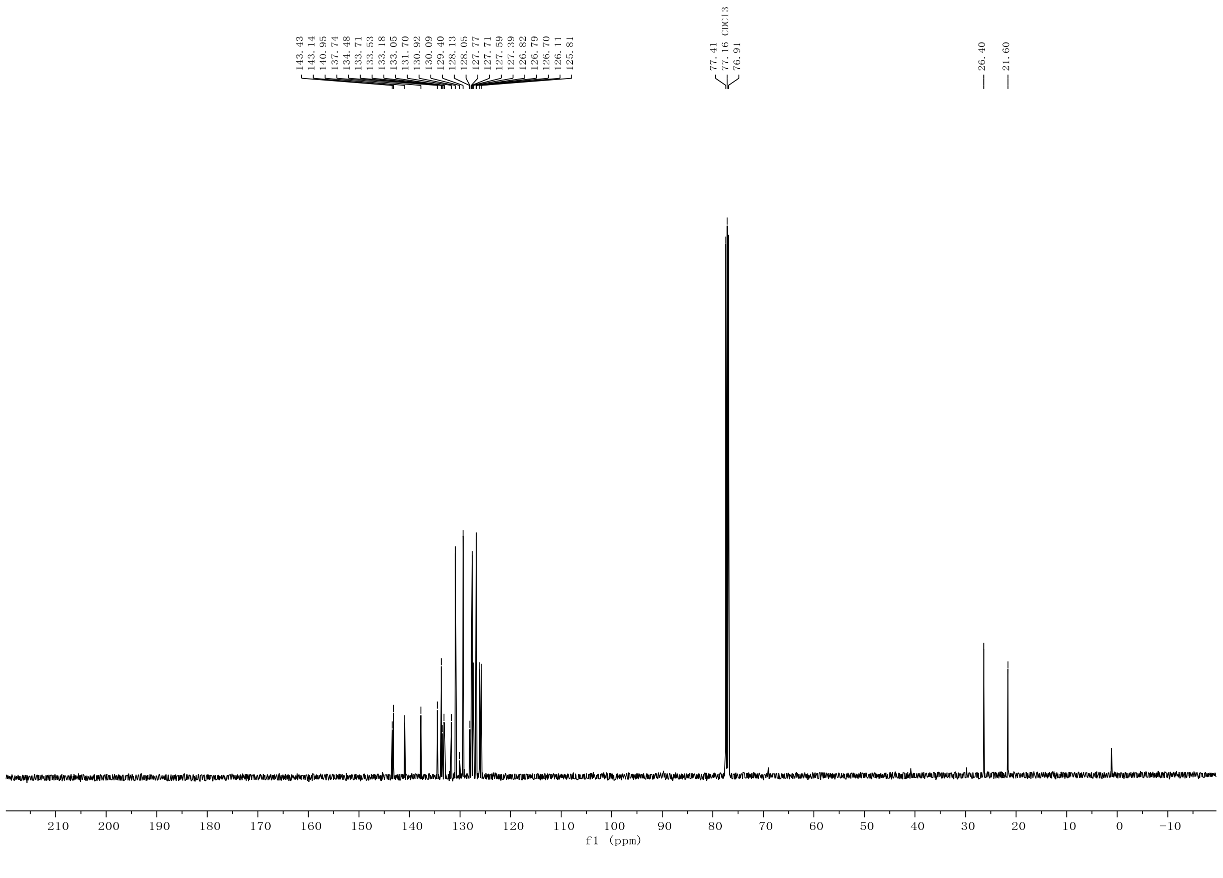

3la ^{1}H NMR (400MHz) and ^{13}C NMR (101MHz)

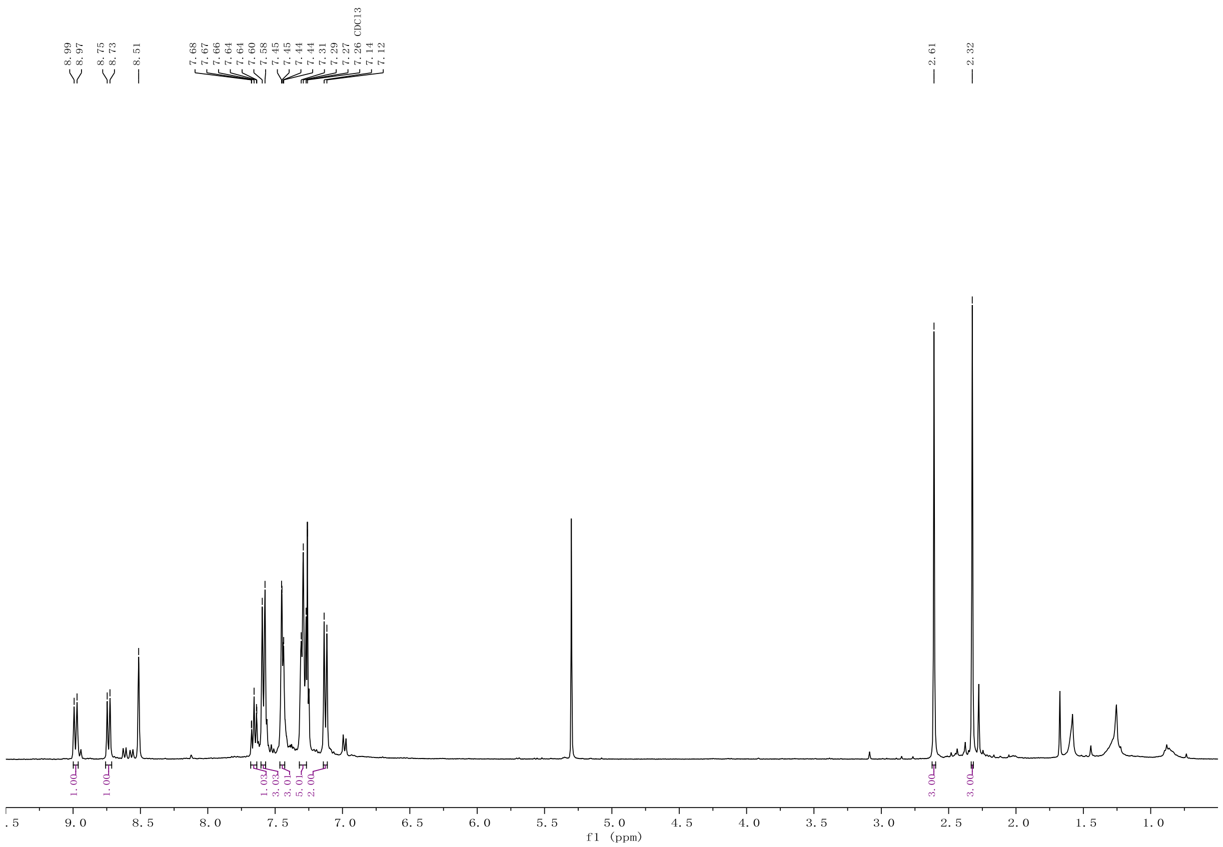

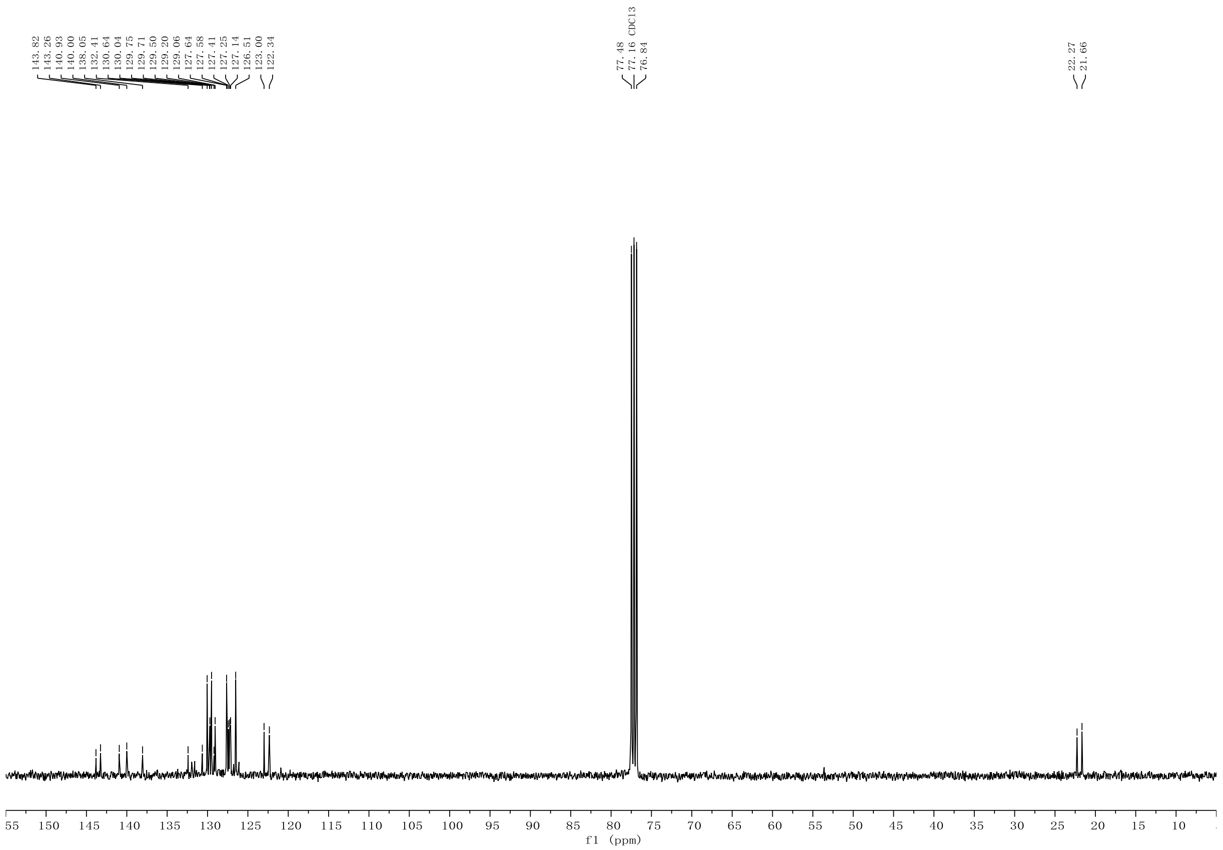

3ma ^{1}H NMR (400MHz) and ^{13}C NMR (101MHz)

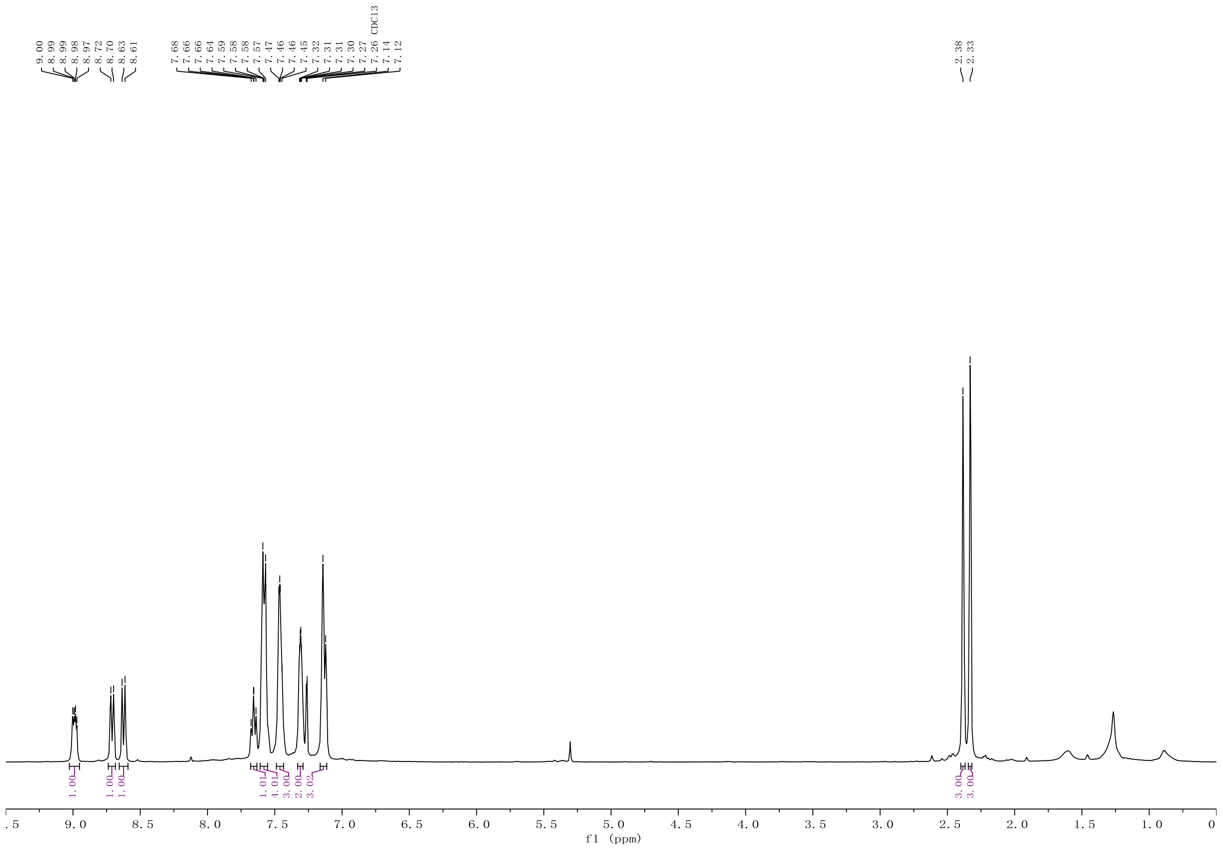

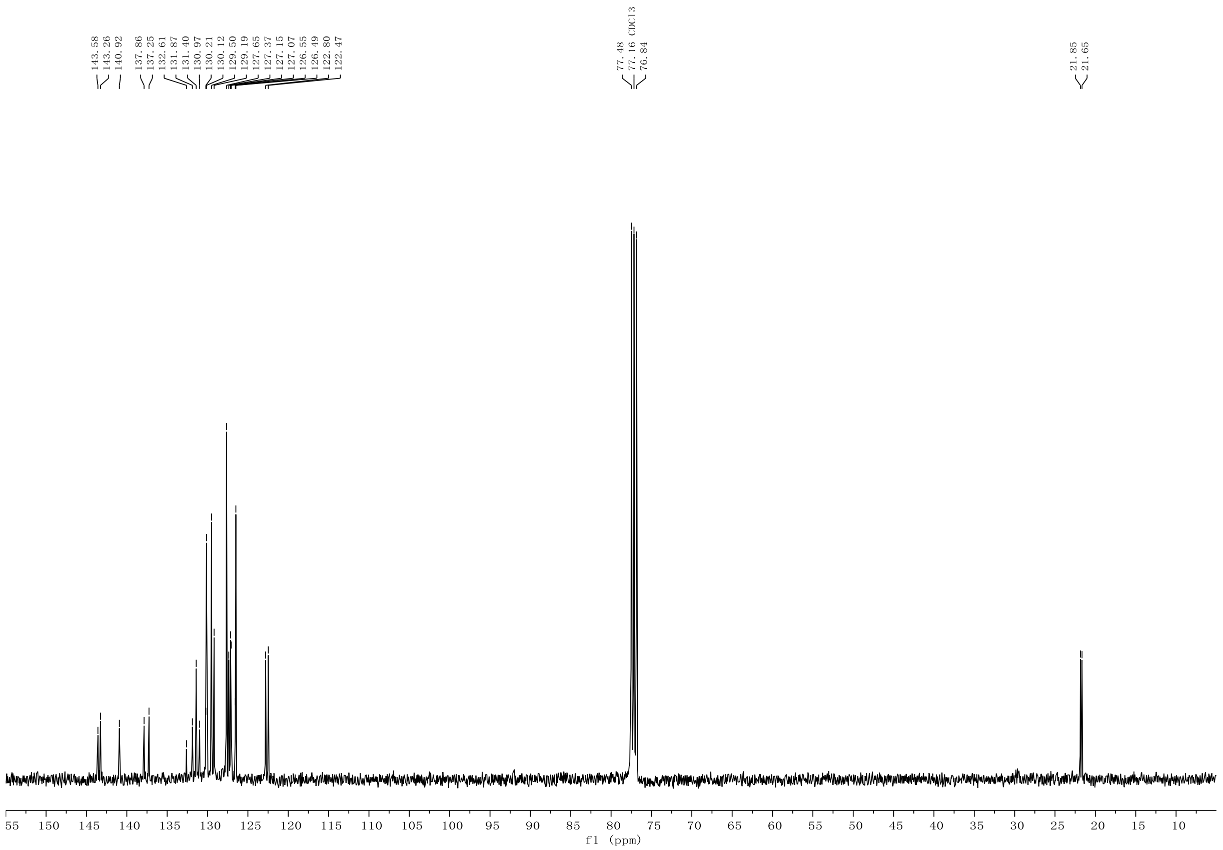

3na ^{1}H NMR (500MHz) and ^{13}C NMR (126MHz)

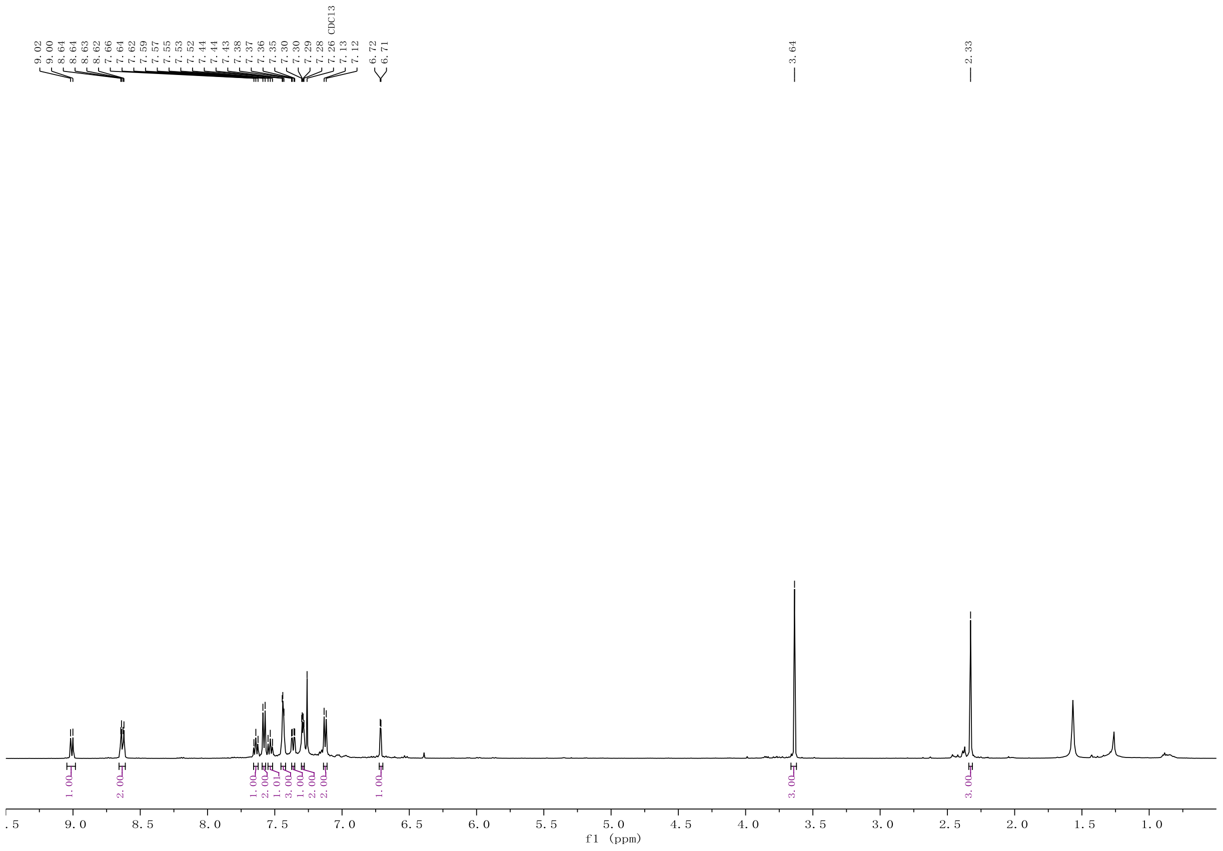

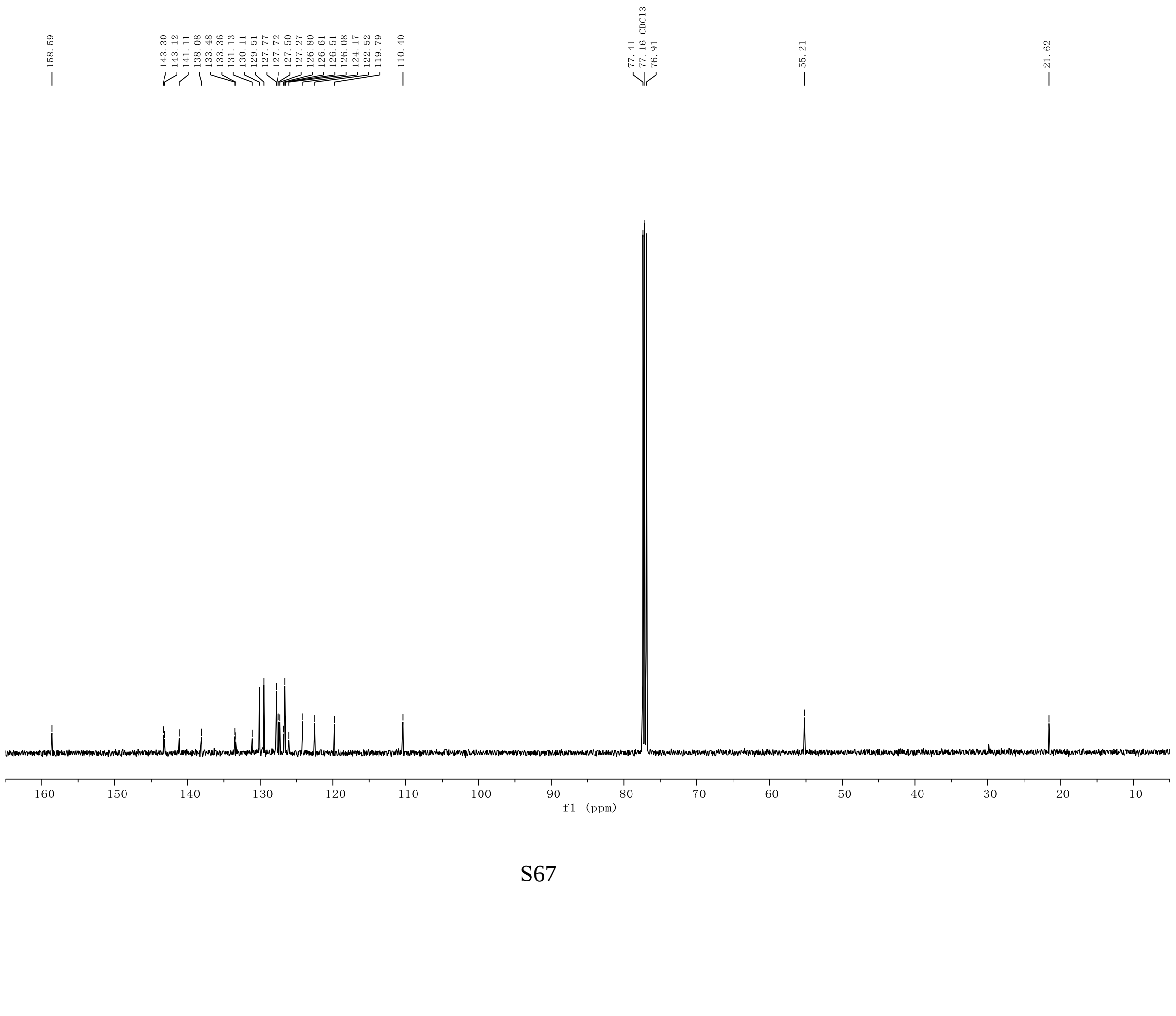

3oa ^{1}H NMR (500MHz), ^{13}C NMR (126MHz**)** and ^{19}F NMR (471 MHz)

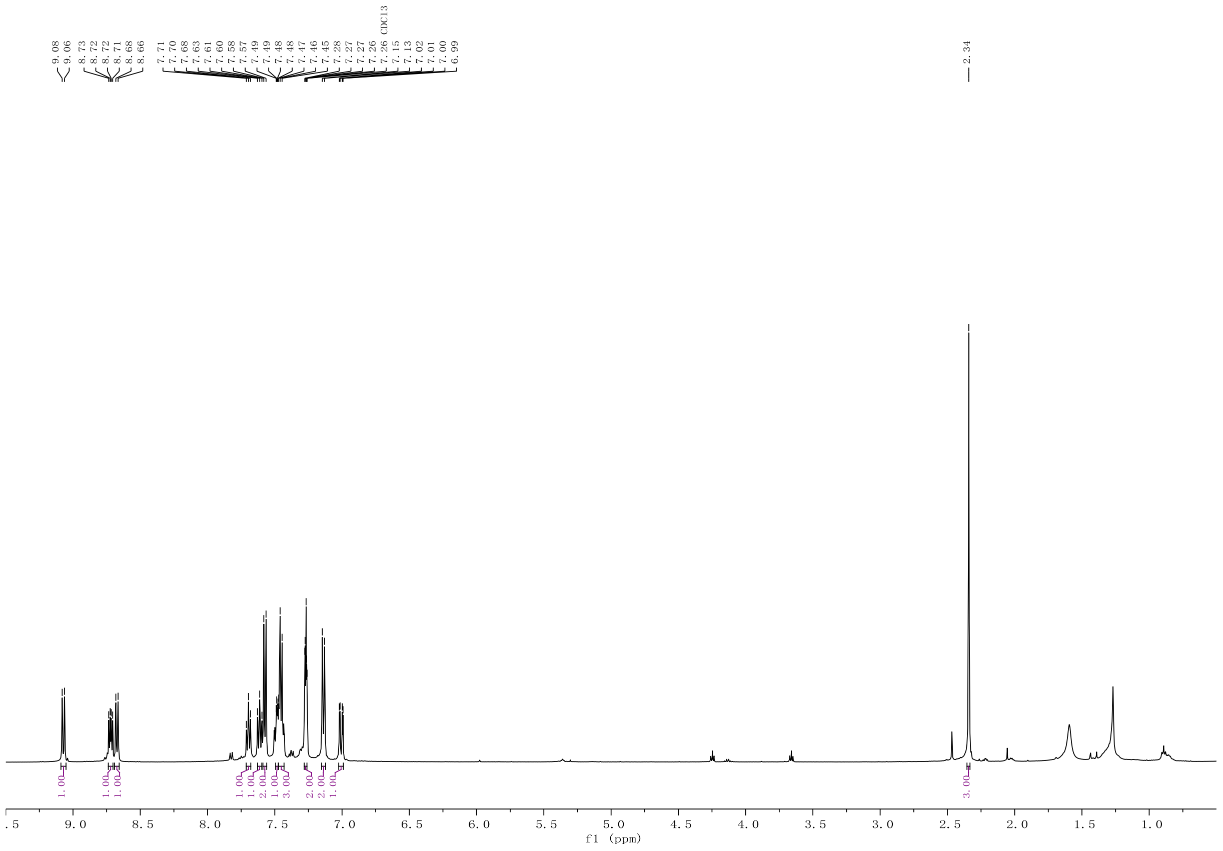

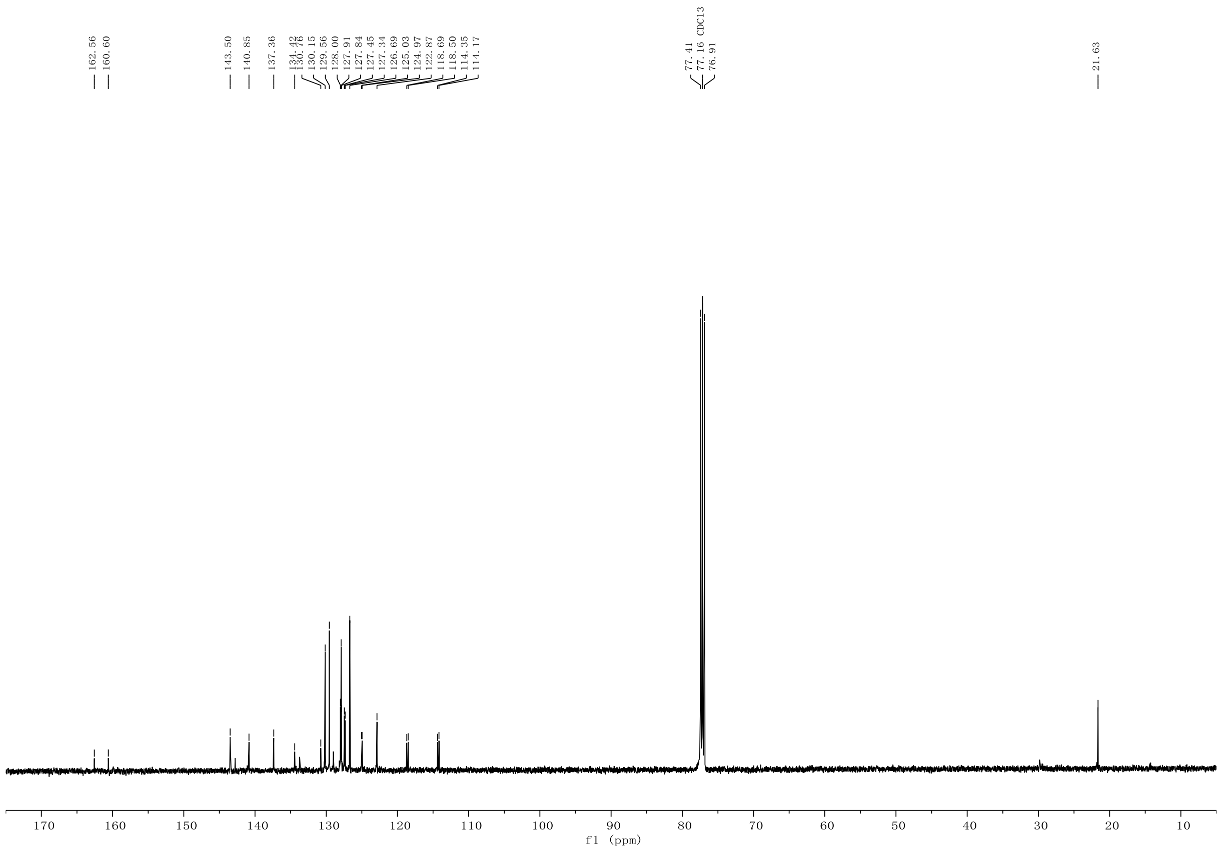

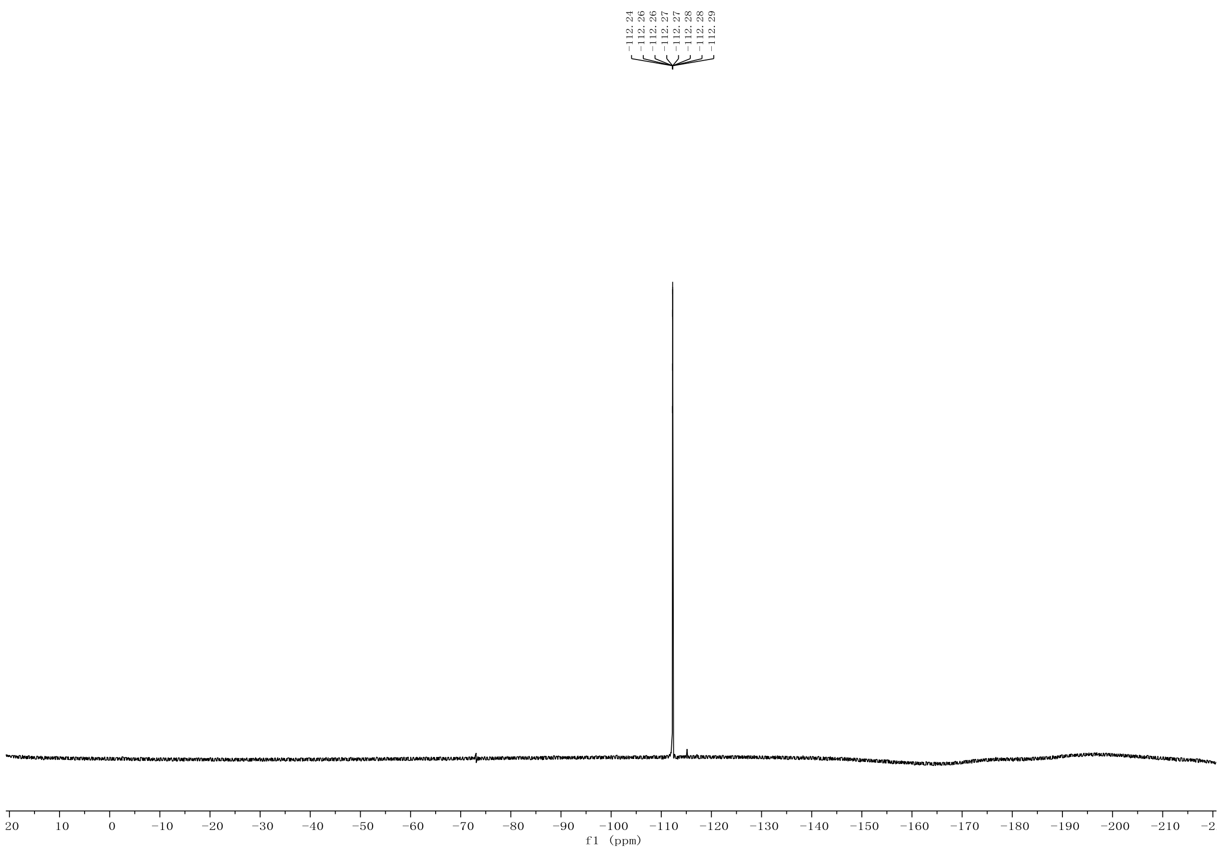
-112.24
-112.26
-112.26
-112.27
-112.27
-112.28
-112.28
-112.29
20 10 0 -10 -20 -30 -40 -50 -60 -70 -80 -90 -100 -110 -120 -130 -140 -150 -160 -170 -180 -190 -200 -210 -2
f1 (ppm)

3pa ^{1}H NMR (500MHz) and ^{13}C NMR (126MHz)

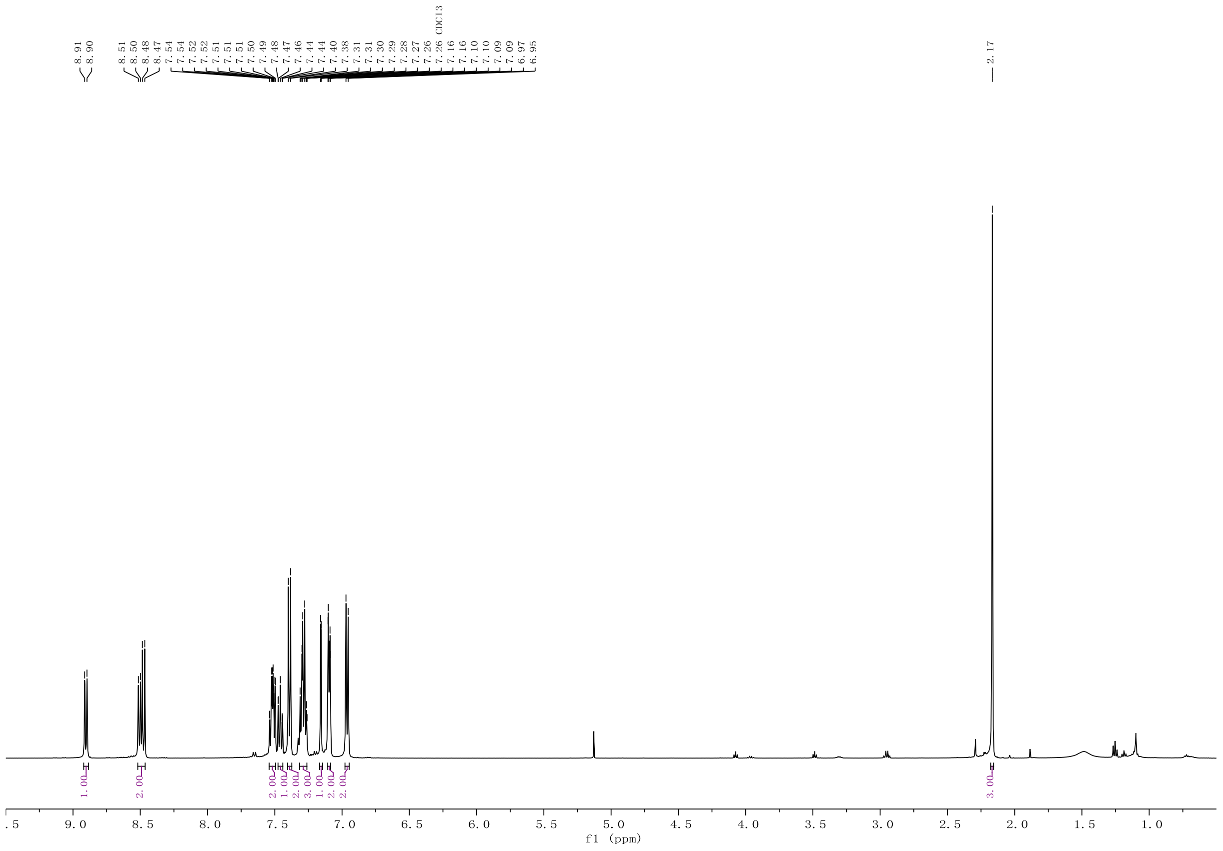

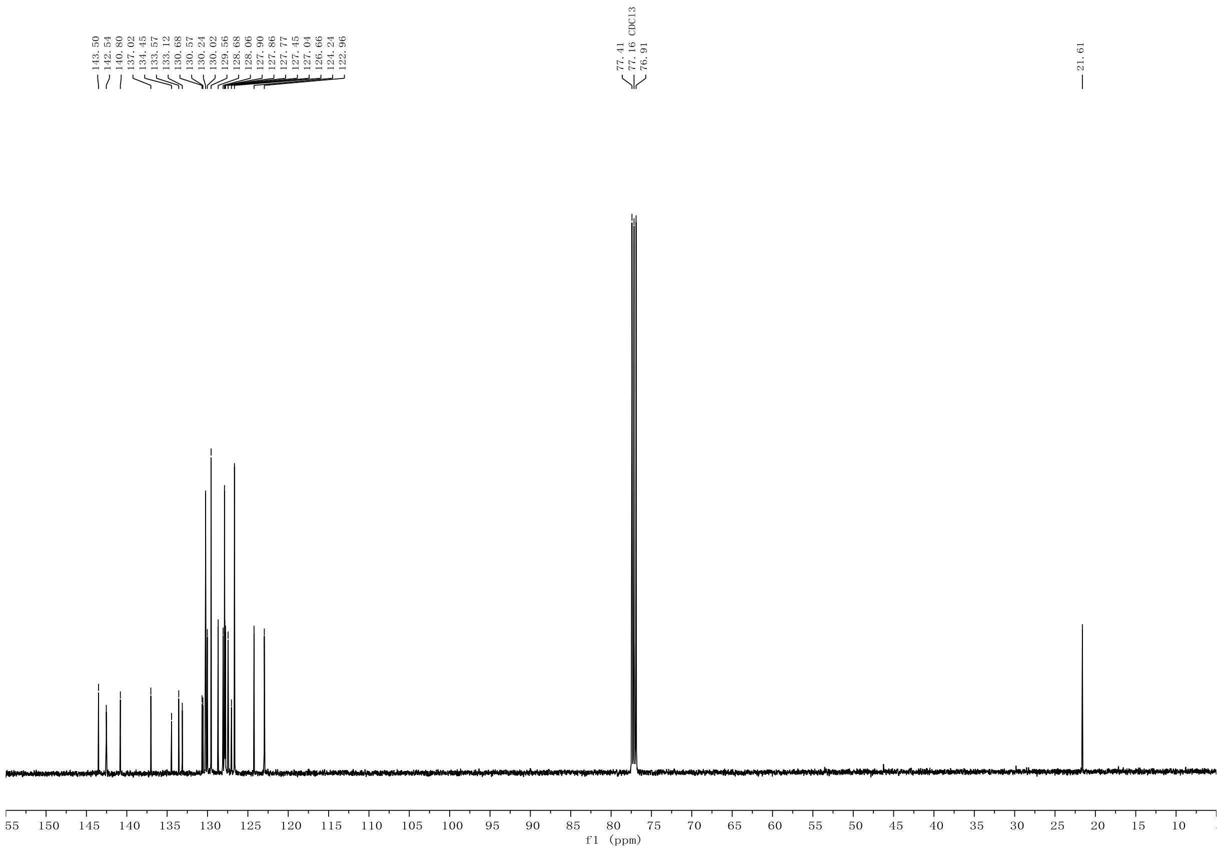

3qa ^{1}H NMR (500MHz) and ^{13}C NMR (126MHz**)**

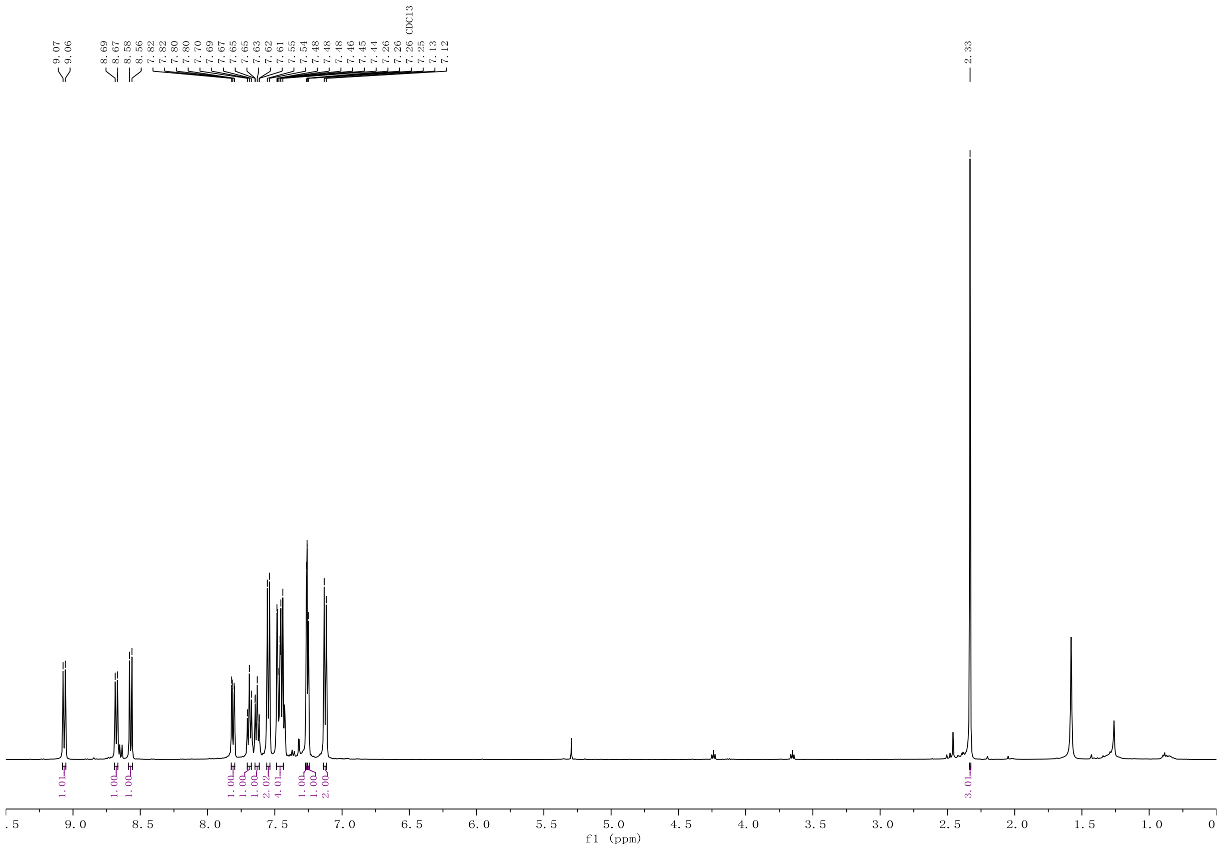

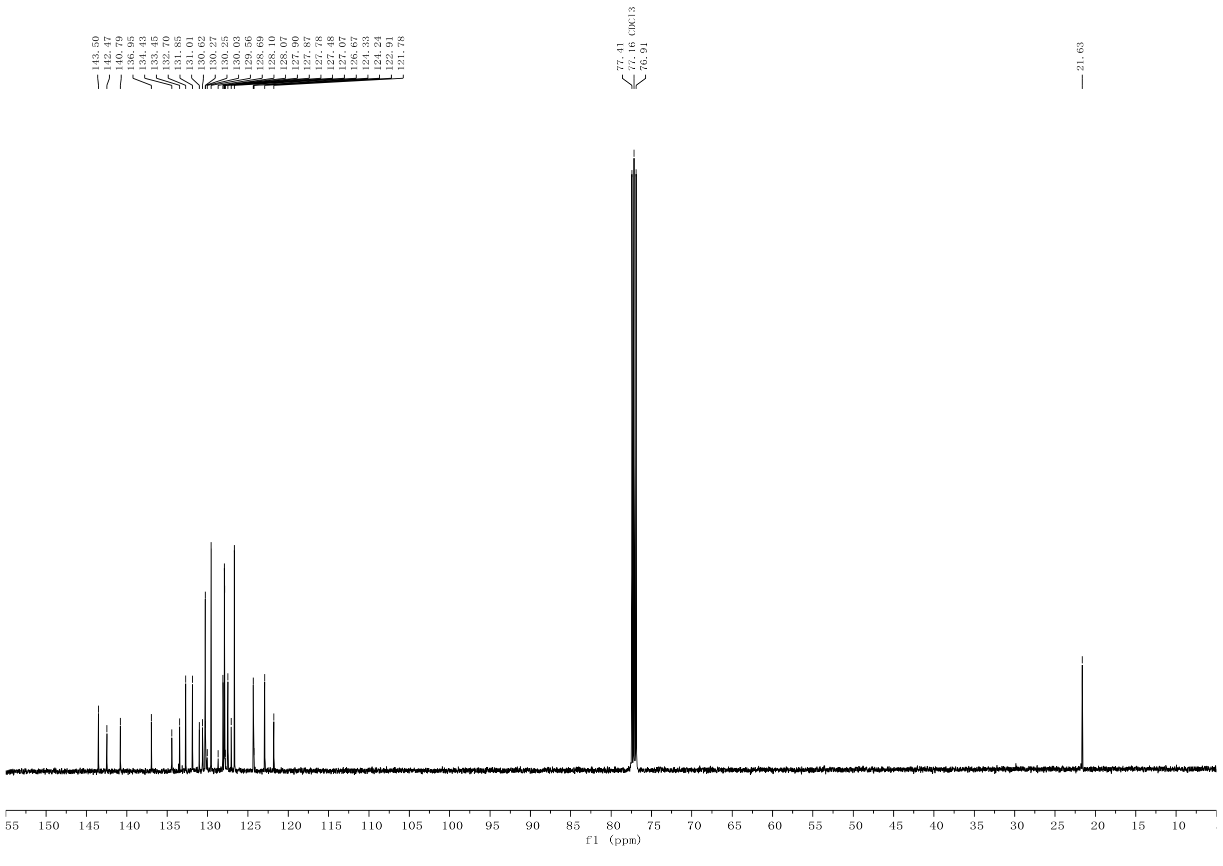

3ra ^{1}H NMR (400MHz) and ^{13}C NMR (101MHz)

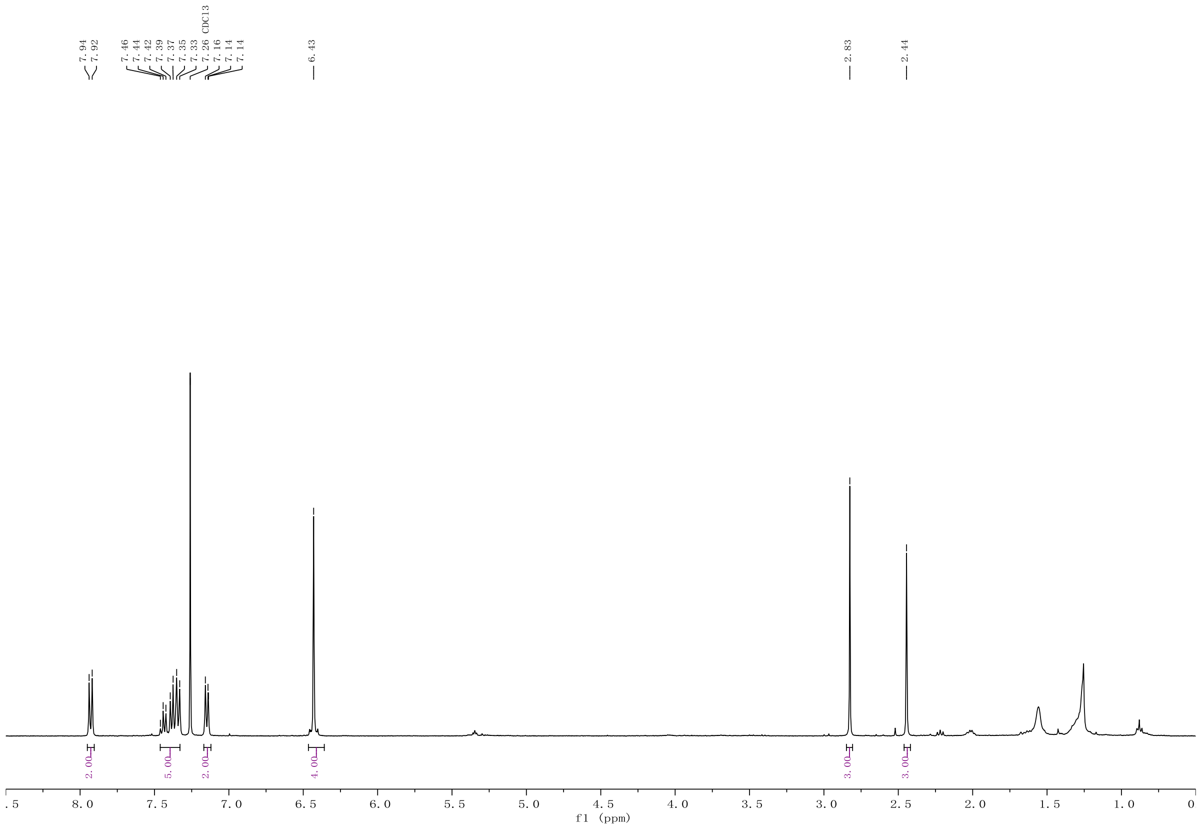

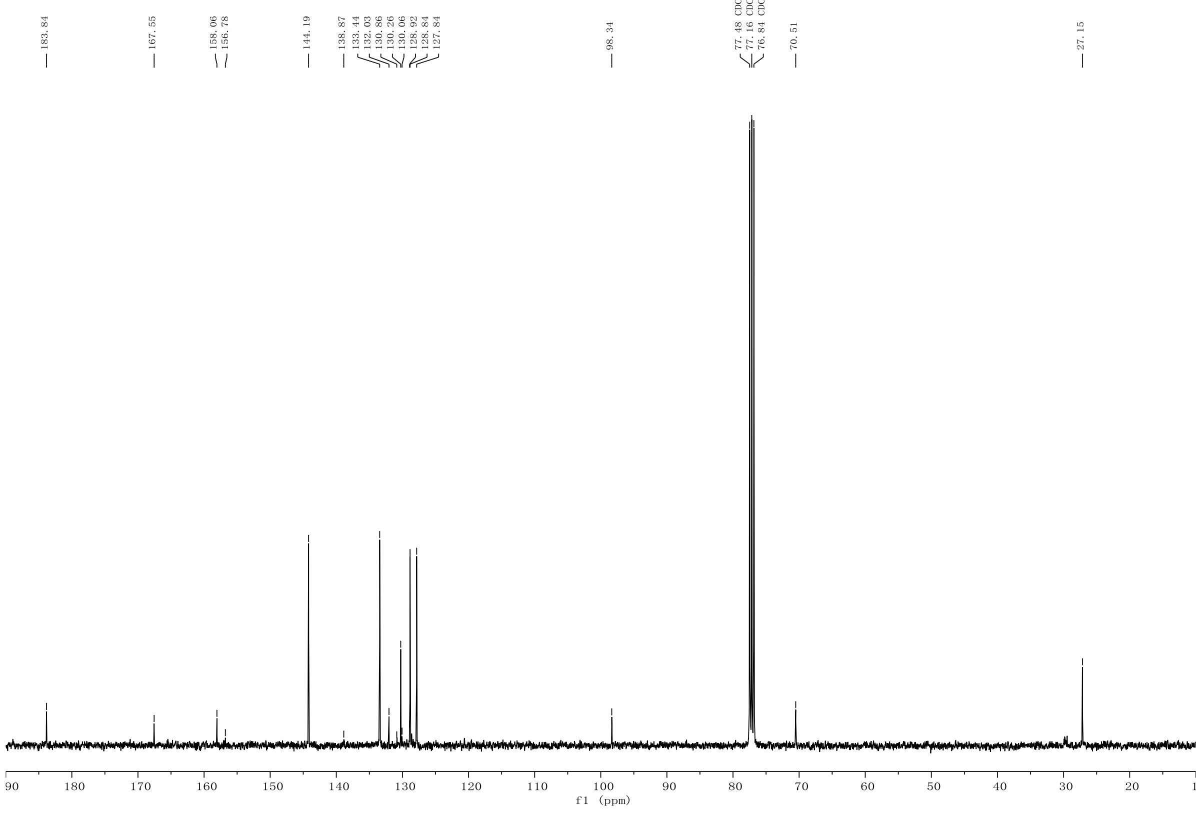

3sq ^1H NMR (400MHz) and ^{13}C NMR (101MHz)

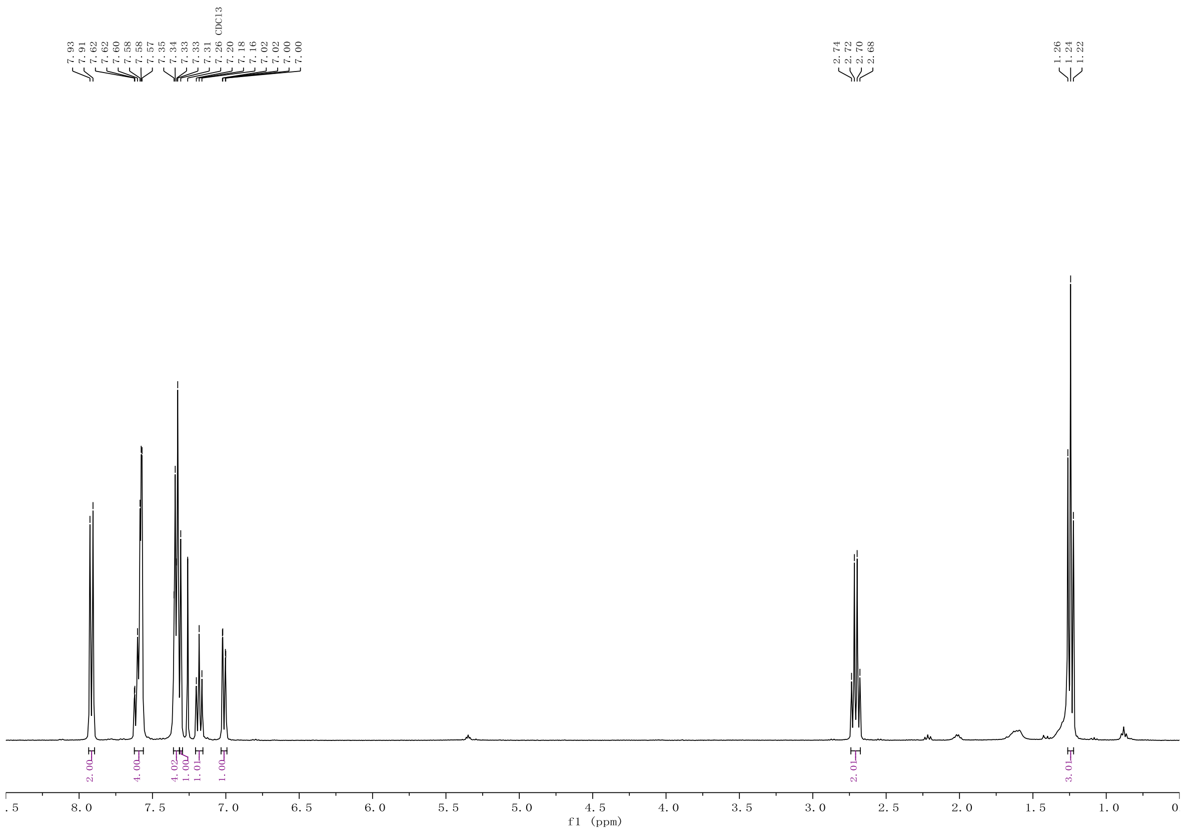

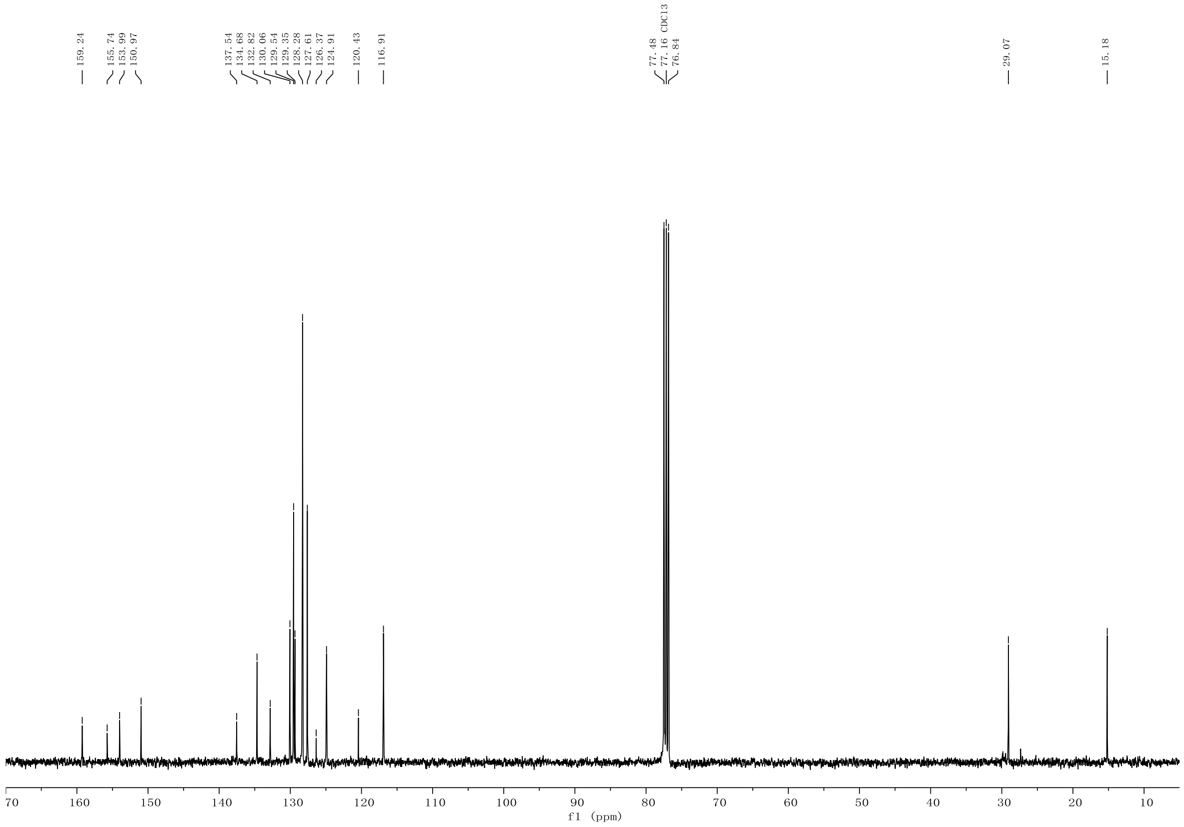

3ta ^{1}H NMR (400MHz) and ^{13}C NMR (101MHz)

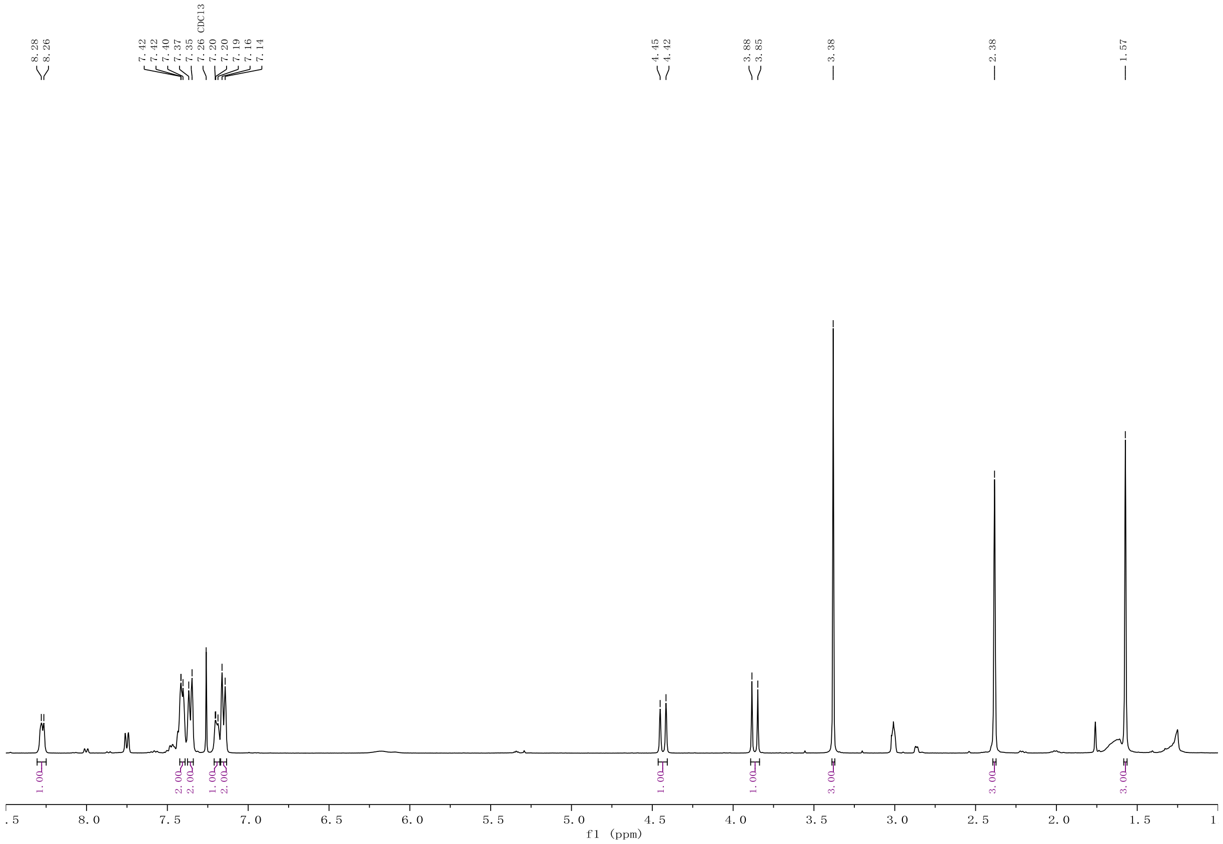

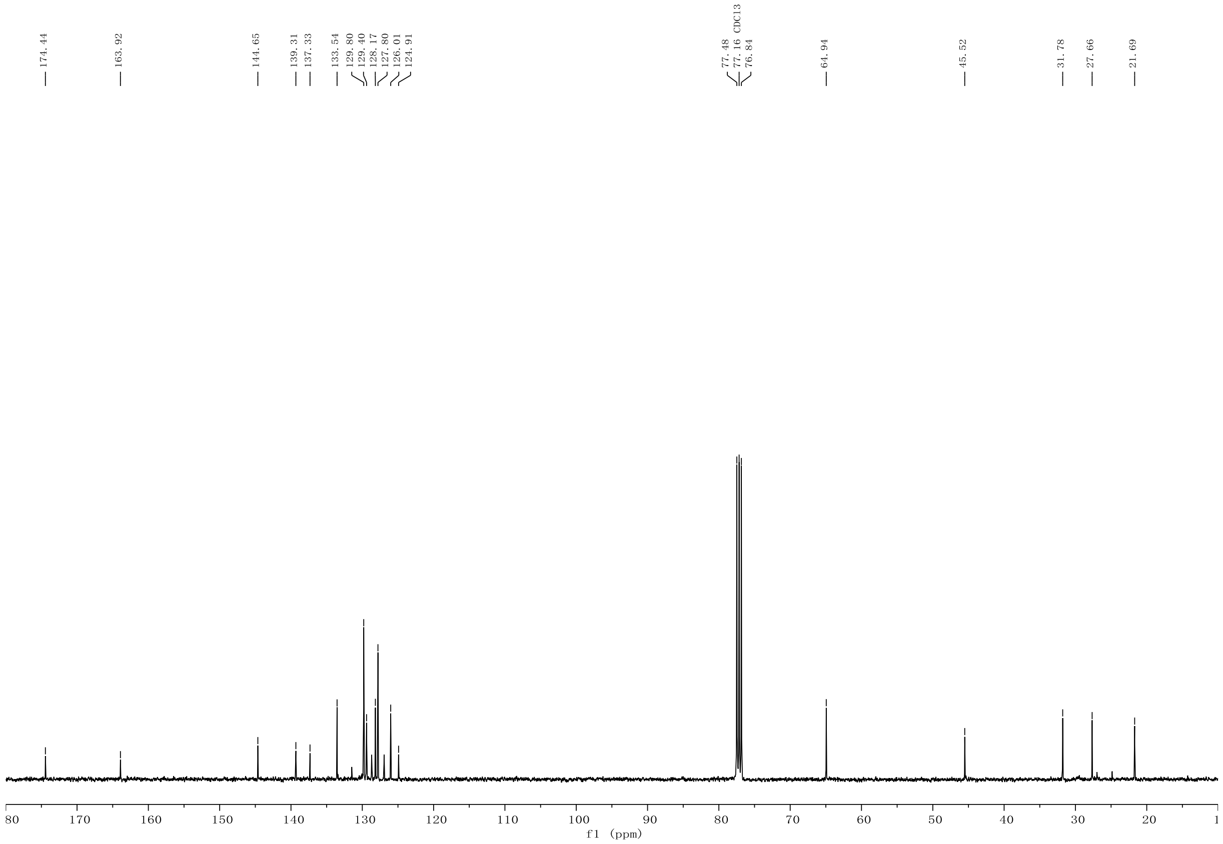

3ua ^{1}H NMR (400MHz) and ^{13}C NMR (101MHz)

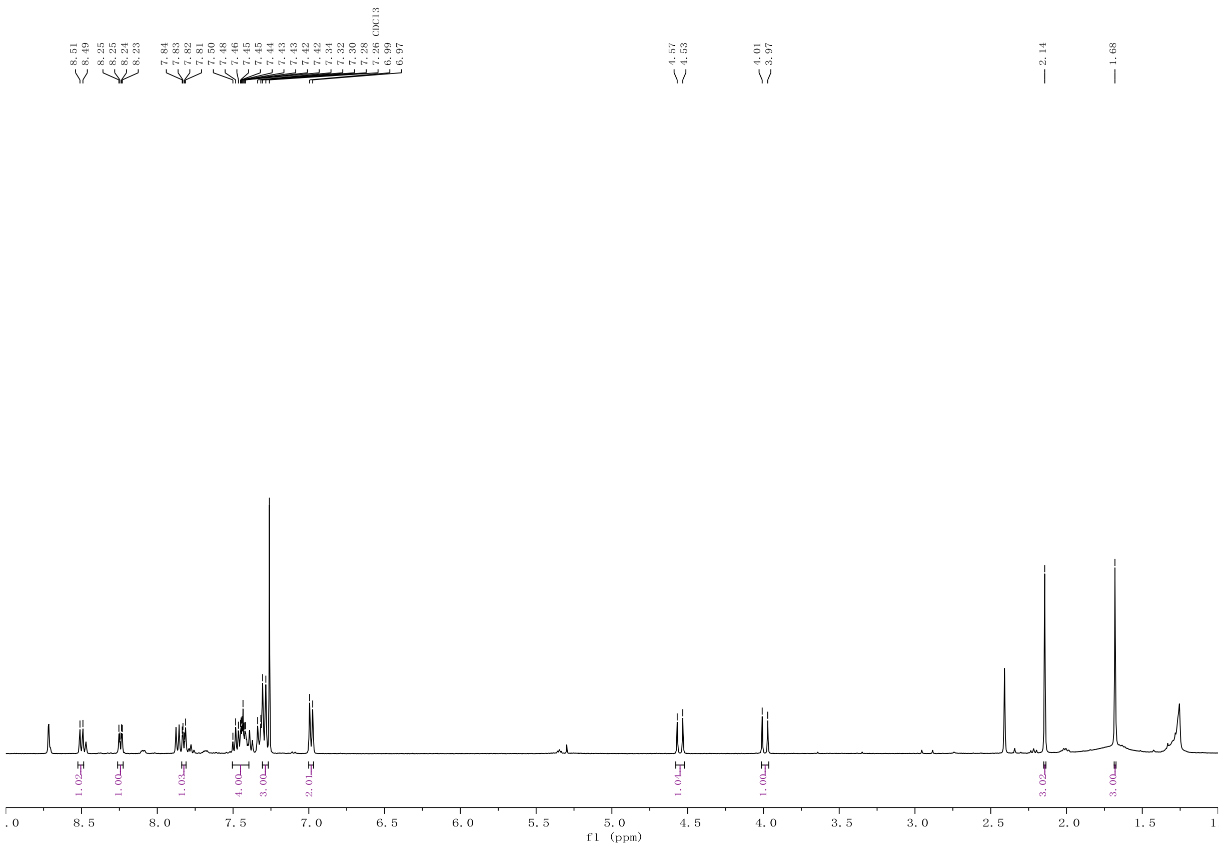

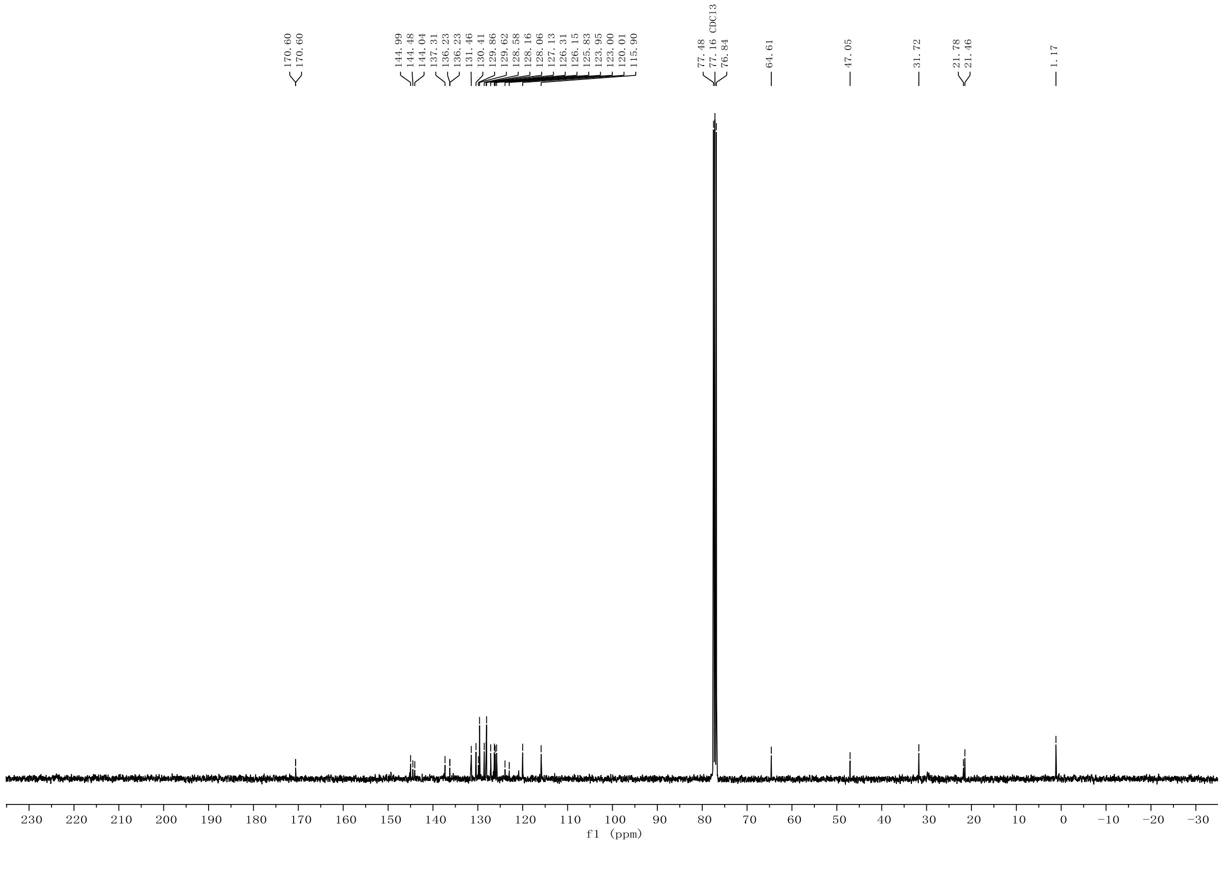

Supplement: Supplementary file 1 — Supporting Information [file ADVS-13-e15993-s001.pdf]
